# Integration of deep transcriptome and proteome analyses of salicylic acid regulation high temperature stress in *Ulva prolifera*

**DOI:** 10.1038/s41598-017-11449-w

**Published:** 2017-09-08

**Authors:** Meihua Fan, Xue Sun, Nianjun Xu, Zhi Liao, Yahe Li, Jianxin Wang, Yingping Fan, Dalian Cui, Peng Li, Zengliang Miao

**Affiliations:** 1 Key Laboratory of Marine Biotechnology of Zhejiang Province, School of Marine Sciences, Ningbo University, Ningbo, 315211 Zhejiang China; 2 Marine Science and Technology College, Zhejiang Ocean University, Zhoushan, 316000 Zhejiang China

## Abstract

To investigate changes in transcript and relative protein levels in response to salicylic acid regulation of the thermotolerance in *U. prolifera*, complementary transcriptome and proteome analyses were performed with *U. prolifera* grown at 35 °C (UpHT) and with the addition of SA at high temperature (UpSHT). At mRNA level,12,296 differentially expressed genes (DEGs) were obtained from the comparison of UpSHT with UpHT. iTRAQ-labeling proteome analysis showed that a total of 4,449 proteins were identified and reliably quantified. At mRNA level, the up-regulated genes involved in antioxidant activity were thioredoxin,peroxiredoxin,FeSOD, glutathione peroxidase, partion catalase and MnSOD. The down-regulated genes were ascorbate peroxidase, glutathione S-transferase, catalase and MnSOD. In addition, the DEGs involved in plant signal transduction pathway (such as auxin response factors, BRI1 and JAZ) were down-regulated. At protein level, the up-regulated proteins involved in carbon fixation and the down-regulated protein mainly were polyubiquitin, ascorbate peroxidase. The expression of Ca^2+^-binding protein, heat shock protein and photosynthesis-related proteins, EDS1 were also significantly regulated both at mRNA and protein level. The results indicated that SA alleviated the high-temperature stimulus through partion antioxidant related proteins up-regulated, JA signal pathway enchanced, Ca^2+^-binding proteins, photosynthesis-related proteins significantly changed, antioxidant enzyme activities increased and photosynthesis index changed.

## Introduction

*Ulva prolifera* (Chlorophyta) belongs to the Ulva genus, a genus with wide temperature and wide salinity tolerance, a large resistance to drying, and a high natural reproduction capacity. It is widely distributed and is the dominant species in the Yellow Sea and East China Sea. To adapt to these environmental changes, *U. prolifera* have developed mechanisms to adapt to the different types of stresses imposed by adverse environments including high temperatures, cold, hypersalinity, and ultraviolet radiation^1, 2^. The life history and nutrients of *U. prolifera* and different environmental effects on the physiology have been investigated. However, the transcriptome and proteome change of SA regulated high temperature in *U. prolifera* are little reported. High temperatures can adversely affect photosynthesis, respiration, water balance and membrane stability. They also modulate hormone levels, primary and secondary metabolites^3^. Recently, numerous reports have demonstrated that phytohormones played an important ﻿role﻿ in plant growth and development as well as biotic and abiotic stresses. Salicylic acid (SA), a small phenolic phytohormone compound, has been shown to play an importamt role in abiotic stresses. Salicylic acid is a plant hormone that can increase plant resistance to drought, cold damage, and resistance to heavy metals and salt damage^4^. Salicylic acid can also significantly improve *Gracilaria* in the survival of the state low-temperature environment^5^. Adding a certain concentration of salicylic acid can improve the high-temperature cultivation of *Gracilariopsis* ^6^. Meanwhile, high-temperature environments induce heat shock protein expression. Salicylic Acid (SA) functions largely by increasing antioxidant gene expression to alleviate lower salt-induced photosynthetic capacity and high temperature stress in *U. prolifera* ^7^. However, the effect of SA on *U. prolifera* is rarely reported. Global transcriptome analysis has revealed that SA may affect the activation of abiotic stress-responsive genes in *Capsicum annuum*, *Lilium lancifolium* and *Arabidopsis thaliana* ^8, 9^ and increase plant resistance to drought, heavy metals, cold and salt damage^10, 11^. There is a strong correlation between gene expression in hormonal regulation and abiotic stress. Despite the close relationship between abiotic stresses and hormone regulation in higher plants, the effects of exogenous hormones on transcriptional levels in algae have not been well studied.

Transcriptome sequencing is a useful method for identifying novel transcripts and analyzing gene expression. Transcriptome analyses of *Pyropia tenera*, *U. prolifera*, *Chondrus crispus*, and three South Pacific kelps have been performed under different stress conditions^12–14^. Most of the transcripts produced under stress conditions belong to the heat shock protein family, and the changed genes are typically involved in the antioxidant response. In addition, genes involved in the algal growth process, the biosynthesis of secondary metabolites, phytohormone biosynthesis and signal transduction, photosynthesis, and amino acid metabolism have been screened and identified^15, 16^. Therefore, macroalgae have developed a variety of strategies and mechanisms to respond to and survive in the face of these environmental stresses^17^. Proteomic sequencing is a key technique for exploring gene changes at the level of translation. The latest iTRAQ (isobaric tags for relative and absolute quantitation) protein quantitative analysis technology is widely used. Proteomics analysis has been used to identify the metabolic responses of *Synechocystis* PCC 6803 with biofuel butanol, ethanol and hexane treatment^18–20^. Integrative transcriptome and proteome analyses have been performed in *Aspergillus flavus* and rice under temperature stress^11, 21^. However, it is rarely reported in the algae.

To understand the mechanism of SA regulation high temperature during *U. prolifera* development at the genomic level, Illumina paired-end sequencing and iTRAQ protein quantitative analysis of SA regulated high temperature were investigated. This comprehensive analysis of the transcriptomes and proteome may substantially improve the global view of the potential molecular mechanisms involved in SA-regulated *U. prolifera* development under high temperature and pave the way for further analysis. The experiment aimed to provides important molecular data support for deep understanding of the physiological and ecological characteristics of *U. prolifera*, and also provides an important clue for further interpretation of the green tide and deep exploration of this species.

## Results and Discussion

### Transcriptome sequencing data generation and assembly

We constructed two cDNA libraries from *U. prolifera*: one library was from gametophyte thalli grown under high temperature (denoted UpHT as the control), and the other library was from gametophyte thalli under SA treatment at high temperature (denoted UpSHT). We obtained 61,573,878 and 59,687,384 raw reads by sequencing the UpHT and UpSHT transcriptomes, and 53,510,640 and 51,854,036 clean reads were obtained after removal of low-quality reads and adaptors from UpHT and UpSHT, respectively (Table 1). The total length of clean nucleotides from UpHT and UpSHT was 4,815,957,600 nt and 4,666,863,240 nt, respectively, and the GC percentage of the transcriptome was 56.64%. The reads were assembled into 126,417 and 114,436 contiguous sequences (contigs), and the contigs were assembled into unigenes after the removal of redundant sequences. This process generated 82,891 and 72,319 unigenes, and 45.9% and 45.01% of the sequences had a length of 200–300 nm in UpHT and UpSHT, respectively.

**Table 1:** Sequencing, assembly and annotation results of the transcriptome of *U. prolifera*.

Samples	Items	Values
UpHT	Total raw reads	61,573,878
	Total clean reads	53,510,640
	Total number of unigenes	82,891
	Total length of unigenes (nt)	72,981,301
	N50 length of assembly	2069
	Mean length of assembly (nt)	880
	Number of unigenes with ORFs	48,997
	Number of annotated unigenes	38,243
UpSHT	Total raw reads	59,687,384
	Total clean reads	51,854,036
	Total number of unigenes	72,319
	Total length of unigenes (nt)	71,099,236
	N50 length of assembly	2312
	Mean length of assembly (nt)	983
	Number of annotated unigenes	38,243

### Functional annotation of the all-unigenes

A total of 38,243 unigenes were annotated with the NCBI-NR, NCBI-NT, Swiss-Prot, KEGG, COG, and GO databases using the BLASTX program. Hits with an e-value of ≤1e^−5^ were accepted. Overall, of the annotated unigenes, 37,539 (98.3%), 7,170 (18.8%), 24,469 (63.98%), 26,857 (70.23%), 22,489 (58.81%) and 18,169 (47.51%) were annotated based on the NR, NT, Swiss-Prot, KEGG, COG and GO databases, respectively. A total of 37,539 unigenes shared homology with existing protein sequences in the Nr database; 19.1% had a perfect match with an e-value ≤e^−60^, and 18.1% had a similarity of greater than 60% (Supplementary Fig. 1A,B). The annotated species distributions associated with the most abundant species of algae were *Chlorella vulgaris* (13.4%), *Volvox carteri* f*. nagariensis* (11.5%), *Chlorella* sp. (10.6%), *Chlamydomonas reinhardtii* (9.9%), *Aureococcus anophagefferens* (6.4%), *Guillardia theta* (4.4%); another 402 species accounted for 40.3% of the total species annotated (Supplementary Fig. 1C).

### DEGs identified and GO analysis

**Figure 1:**
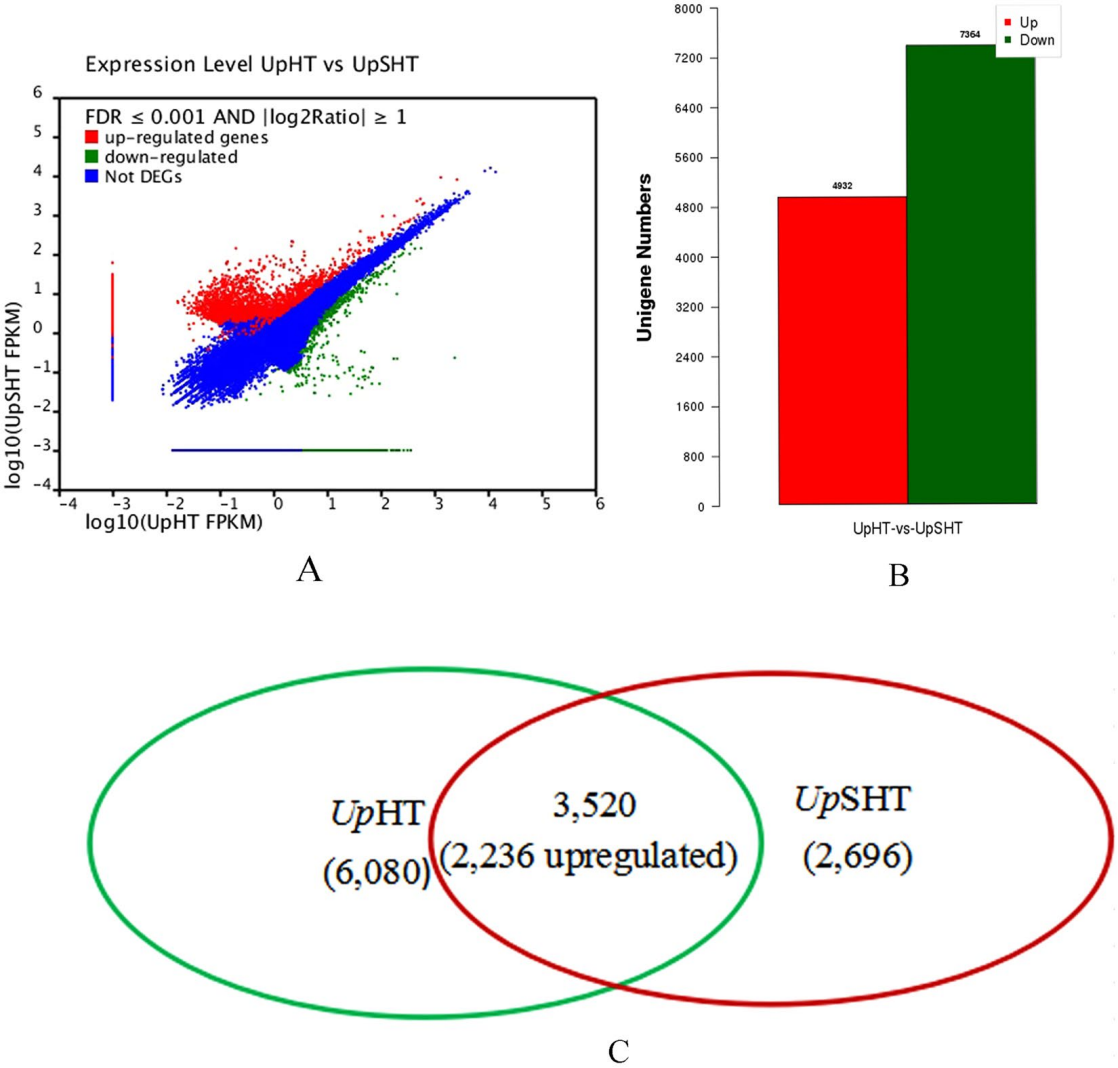
DEGs expression levels of UpHT (control) vs UpSHT (treatment). (**A**) The volcano figure (**B**) column diagram (**C**) Venn diagram. The genes were classified into three classes. Red genes are up-regulated, green genes are down-regulated. Blue genes are not differentially expressed genes. The horizontal coordinate is the expression level of UpHT sample, and the vertical coordinates is the expression level of the UpSHT sample.

To identify genes involved in SA regulated temperature stress changes in *U. prolifera* differentially expressed genes (DEG) were detected using the DEGseq tool. We obtained 12,296 DEGs by FDR ≤ 0.001 and llog2(UpSHT/UpHT)l ≥ 1 in the UpSHT compared to UpHT condition. (Fig. 1A). In the UpSHT treatment, 4,932 responsive genes were up-regulated; 2,696 genes were only detected under the UpSHT condition, and 7,364 genes were down-regulated compared to the UpHT condition (Fig. 1B). In addition, among the down-regulated genes, 6,080 genes were only detected under the UpHT condition (Fig. 1C). These data demonstrated that approximately 6.5% of the transcripts were up-regulated and 9.7% were down-regulated by at least 2-fold by SA induced under high temperatures in the gametophyte thalli of *U. prolifera*.

**Figure 2:**
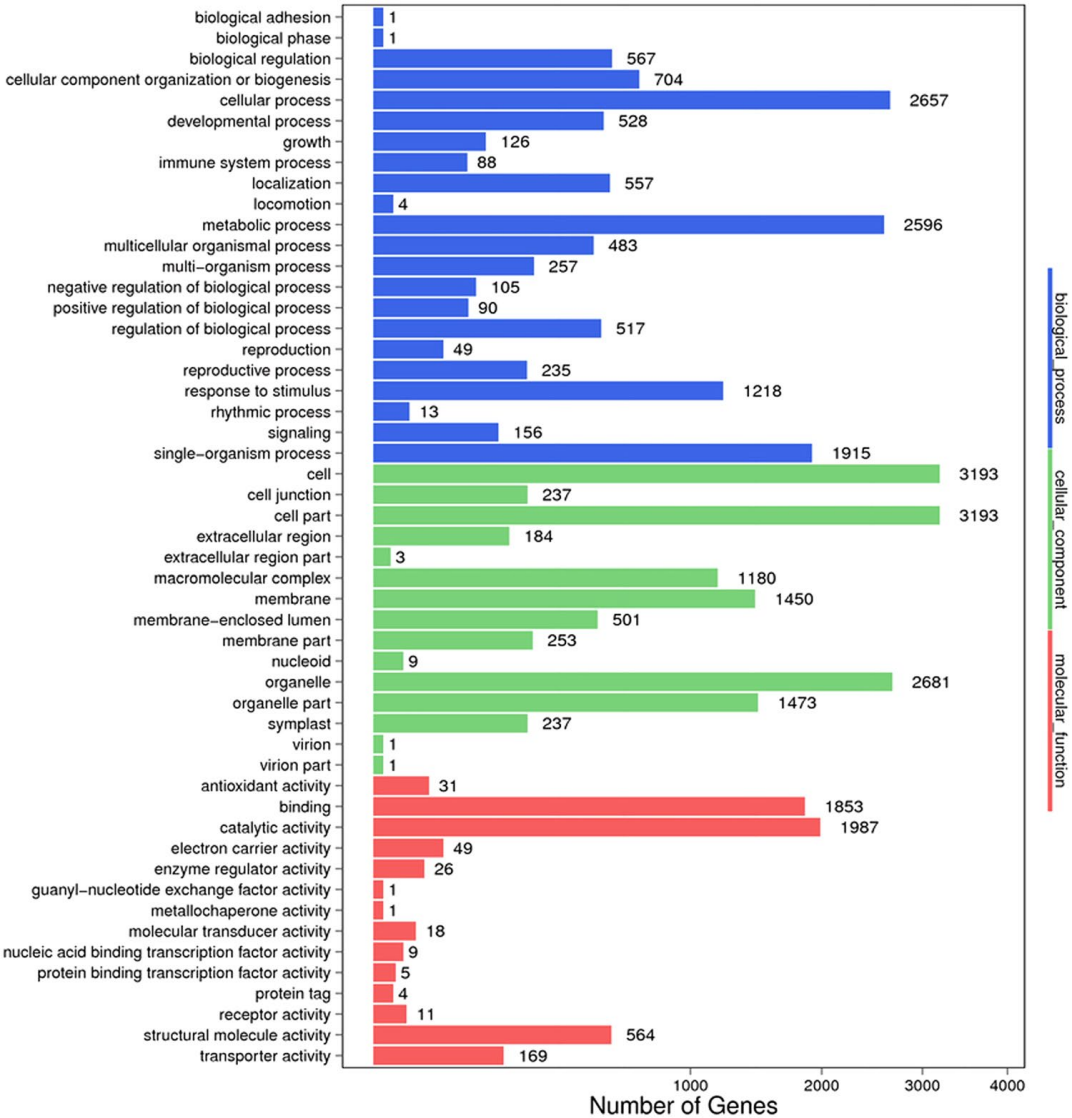
GO functional annotation histogram of the DEGs. The ordinate coordinates represent three GO categories under the level of the GO term; the abscissa is the annotation of the term, the gene number, and the number of genes accounted for by differences in the proportion of the total. Three different classifications of GO annotations of three basic categories are included (from top to bottom: biological processes, cellular component, and molecular function).

Using the Blast2GO platform, we classified 4,212 differentially expressed genes with GO terms according to their functions and found that most abundant GO terms included “biological processes”, “molecular functions” and “cellular components” (Fig. 2). Many genes were associated with cellular process, cell, organelle, and metabolic process. The most abundant categories were cellular process (2,657, 63.08%), metabolic process (2,596, 61.63%) and single organism process (1,915, 45.46%), as well as response to stimulus (1,218, 28.92%) in the biological processes category. In addition, genes involved in growth, immune system process, and signaling accounted for 2.99%, 2.08% and 3.70% of the total genes, respectively. The highest abundances for the cellular component category were for cells (3,193, 75.80%), cell part (3,193, 75.80%) and organelle (2,681, 63.65%). In the molecular function category, most of the unigenes were classified into the binding (1,853, 43.99%) and catalytic activity (1,987, 47.17%) functions, whereas the antioxidant activity function only accounted for 0.45% of the total unigenes. In addition, the stress stimuli were differentially expressed, with only 455 genes up-regulated and 763 genes down-regulated in the total group of 1,218 genes.

### DEGs metabolic pathways

**Figure 3:**
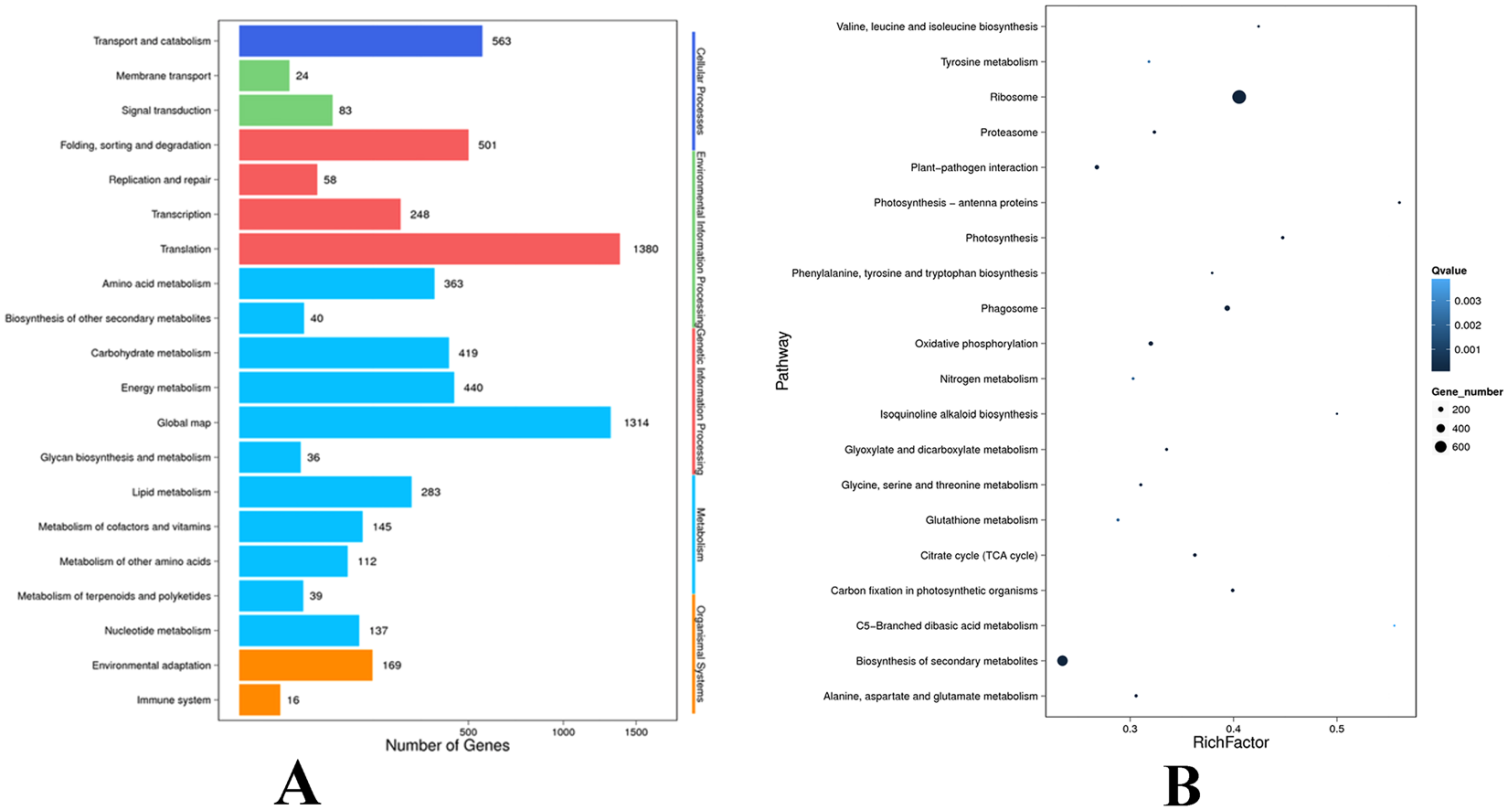
KEGG analysis of DEGs (**A**)﻿ and Pathway enrichment statistical scatter plot of DEGs (**B**). (**﻿A**) The vertical axis represents the name of the pathway; the horizontal axis represents the number of genes. (**B﻿**) The vertical axis represents the name of the pathway; the horizontal axis represents the pathway corresponding rich factor. The rich factor refers to the ratio of the number of differentially expressed genes in the pathway and the number of all annotated genes in the pathway. Higher rich factors indicate greater degrees of enrichment. Q values are often completed after multiple hypothesis testing with corrected *P* value values ranging from 0 to 1. The closer they are to zero, the more significant the enrichment. The figure only shows the enrichment degree of the top 20 entries in the pathway.

To survey genes involved in important metabolic pathways, we mapped the annotated *U. prolifera* transcripts to KEGG pathways. Using the Blast2GO program, 5,163 DEGs were mapped to the KEGG database, and DEGs were enriched at the translation level (1380, 26.73%) and global map (1314, 25.45%). In addition, 83 DEGs were enriched at the signal transduction level, 169 DEGs were enriched in environmental adaptation, and 16 DEGs were enriched at the immune system level (Fig. 3A). The 5,163 DEGs were mapped to the 120 KEGG pathways using the Blast2GO platform. To identify differentially regulated biological processes, we performed functional pathway enrichment analyses for differentially expressed proteins. The pathways enriched were represented “ribosome” (729, 14.12%), “phagosome” (208, 4.03%), “photosynthesis” (81, 1.57%), photosynthesis - antenna proteins (51, 0.99%), carbon fixation in photosynthetic organisms (95, 1.84%), oxidative phosphorylation (152, 2.94%), citrate cycle (TCA cycle) (95, 1.84%), proteasome (87, 1.69%), glyoxylate and dicarboxylate metabolism(58,1.12%), “biosynthesis of secondary metabolites” (526, 10.19%), “protein processing in endoplasmic reticulum” (229, 4.44%), “plant-pathogen interaction” (143, 2.77%), glutathione metabolism (66, 1.28%), Nitrogen metabolism (53, 1.03%), tyrosine metabolism (42, 0.81%), *et al*. (Fig. 3B). The pathway enrichment analysis showed that the C5 branched dibasic acid and photoprotein antenna protein pathways were the most enriched pathways in the DEGs (Fig. 3B).

### Annotation of proteome data

This experiment generated 283,344 spectra, of which, 90,247 spectra matched known peptides and 78,143 spectra matched unique peptides. Ultimately, 15,546 peptides, 14,885 unique peptides, and 4449 proteins were identified. 2230 (50.12%) and 3511(78.9%) of 4449 identified proteins mapped to the GO annotation and COG annotation (Supplementary, Fig. 2). In the COG function analysis, the largest category was “general function prediction only” (555, 15.8%), followed by “translation, ribosomal structure and biogenesis” (390, 11.11%), “posttranslational modification, protein turnover, chaperones” (336, 9.56%). In the GO function analysis, the most abundant groups were cell, cell part and metabolic process.

**Figure 4:**
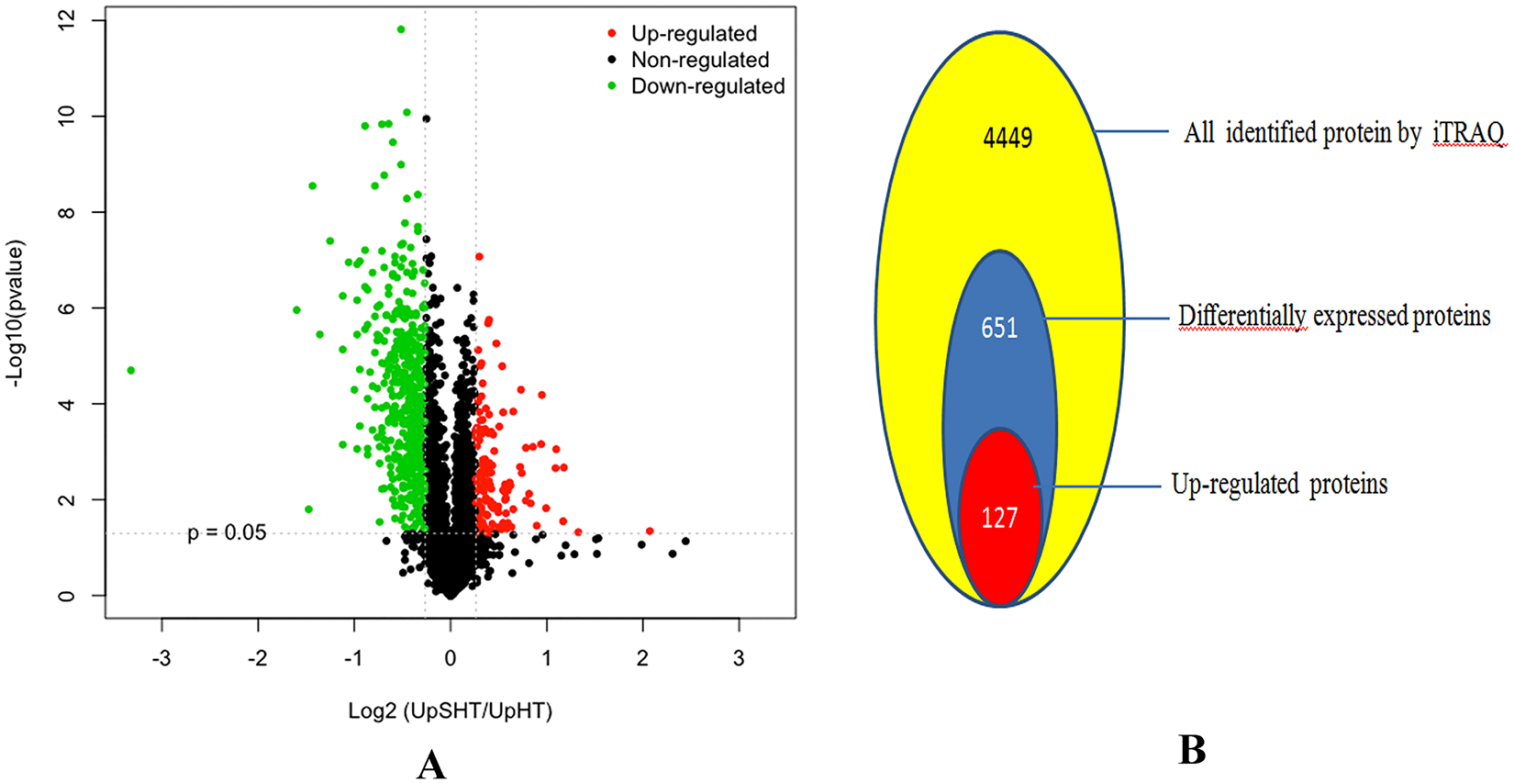
Identification of DEPs. A Volcano plots of DEPs under UpSHT compare with UpHT. The green indicated down-regulated proteins, red indicated up-regulated proteins, black indicated no significantly change. B the number of proteins identifed and DEPs (UpSHT VS UpHT).

Using volcano plot, a scatter-plot that allows evaluation of quantitative data from two parameters, such as fold change and the correspondent p values, we recognized that proteins with significant quantitative ratios between the two treatments (*p* < 0.05) and with fold changes >1.2 or <0.83 were considered differentially expressed responded to the SA regulation at high temperature. In brief, a total of 651 proteins were found to be significantly changed in *U. prolifera* between UpSHT and UpHT indicating that almost 14.63% of the *U. prolifera* proteins changed their abundance significantly in response to the SA regulation (Fig. 4). Subsequently, 127 up-regulated and 524 down-regulated proteins were identified, the list of protein were showed in Supplementary File 1 or Table 2.

**Table 2:** UpSHT VS UpHT differently expression protein list (portion).

No	Protein_ID	Description	Accession no	*P* value	GO annotation or KEGG pathway
Up-regulated					
490	gi|807046156|gb|AKC35214_1|	photosystem I reaction center subunit IX (chloroplast) [*Ulv*a *fasciata*]	gi|304322949|ref|YP_003795489.1|	0.006013	photosynthesis
960	gi|960515081|gb|ALR86928_1|	photosystem II D2 protein (chloroplast) [*Ulva fasciata*]	gi|108796979|ref|YP_636221.1|	0.01478	photosynthesis
311	gi|145568329|gb|ABP82521_1|	ribulose-1,5-bisphosphate carboxylase/oxygenase large subunit, partial (chloroplast) [Ulva sp. A027747]	gi|145568329|gb|ABP82521.1|	0.0001664	Carbon fixation in photosynthetic organisms
1073	gi|145567817|gb|ABP82265_1|	ribulose-1,5-bisphosphate carboxylase/oxygenase large subunit, partial (chloroplast) [*Ulva* sp. A027367]	gi|404249490|gb|AFR53903.1|	0.0008819	Carbon fixation in photosynthetic organisms
1230	gi|125577|sp|P19824_1|KPPR_CHLRE	phosphoribulokinase precursor [*Chlamydomonas reinhardtii*]	gi|159471788|ref|XP_001694038.1|	0.03031	Carbon fixation in photosynthetic organisms
1767	gi|145567588|gb|ABP82151_1|	ribulose-1,5-bisphosphate carboxylase/oxygenase large subunit, partial (chloroplast) [Ulva sp. A027397]	gi|145568577|gb|ABP82645.1|	0.005011	Carbon fixation in photosynthetic organisms
2641	Unigene42677_All	aspartate aminotransferase [*Volvox carteri f nagariensis*]	gi|302846355|ref|XP_002954714.1|	0.004925	Carbon fixation in photosynthetic organisms
2674	gi|145567931|gb|ABP82322_1|	ribulose-1,5-bisphosphate carboxylase/oxygenase large subunit, partial (chloroplast) [*Ulva* sp. A027743]	gi|145568333|gb|ABP82523.1|	0.00169	Carbon fixation in photosynthetic organisms
2781	gi|1172455|sp|P41758_1|PGKH_CHLRE	phosphoglycerate kinase precursor [*Chlamydomonas reinhardt*ii]	gi|1172455|sp|P41758.1|PGKH_CHLRE	0.002125	Carbon fixation in photosynthetic organisms
3019	gi|158273526|gb|EDO99315_1|	cytochrome P450, CYP85 clan, partial [*Chlamydomonas reinhardtii*]	gi|159481496|ref|XP_001698815.1|	0.0001501	Brassinosteroid biosynthesis
268	CL7120_Contig2_All	soluble carbonic anhydrase precursor [*Chlorella sorokiniana*]	gi|3133261|dbj|BAA28217.1|	0.0001231	Photosynthesis
2111	CL2355_Contig1_All	rieske [2Fe-2S] protein [*Chlamydomonas reinhardtii*]	gi|159477387|ref|XP_001696792.1	0.0003328	Photosynthesis
2831	gi|1001185378|gb|AML80578_1|	cytochrome c oxidase subunit 1 (mitochondrion) [*Ulva linza*]	gi|1001185378|gb|AML80578_1|	0.01879	Photosynthesis
4324	gi|452119419|gb|AGG09538_1|	elongation factor Tu, partial (chloroplast) [*Ulva prolifera*]	gi|452119405|gb|AGG09531.1|	0.006994	Photosynthesis
Down-regulated					
2634	Unigene12134_All	hypothetical protein OsI_20631 [*Oryza sativa Indica Group*]	gi|218197104|gb|EEC79531.1|	0.0003654	response to stimulus
3118	Unigene14048_All	Mov34-domain-containing protein [*Coccomyxa subellipsoidea* C-169]	gi|384247775|gb|EIE21261.1|	4.30E-09	response to stimulus
695	Unigene6725_All	polyubiquitin [*Aureococcus anophagefferens*]	gi|323454622|gb|EGB10492.1|	0.01657	antioxidantactivity binding catalytic activity
1321	Unigene13607_All	thioredoxin peroxidase (ISS) [*Ostreococcus tauri*]	gi|308807377|ref|XP_003080999.1|	0.0004694	antioxidant activity catalytic activity
2697	gi|300256840|gb|EFJ41098_1|	L-ascorbate peroxidase [*Volvox carteri f_ nagariensis*]	gi|302852684|ref|XP_002957861.1|	9.63E-05	antioxidant activity binding catalytic activity
3282	CL2017_Contig1_All	GPX1b [*Chlorella* sp..]	gi|385258209|gb|AFI55002.1|	3.70E-05	antioxidant activity catalytic activity
848	Unigene1814_All	17_8 kDa class I heat shock protein [*Arabidopsis thaliana*]	-	0.0002223	heat shock protein
622	gi|315319013|gb|ADU04518_1|	LhcSR [*Ulva prolifera*]	gi|315319013|gb|ADU04518.1|	0.03226	photosynthesis
168	Unigene43365_All	10 KDa phosphoprotein of photosystem II *[Pseudendoclonium akinetum*]	gi|108796941|ref|YP_636241.1|	0.001919	photosynthesis
462	gi|807046094|gb|AKC35152_1|	ATP synthase subunit b (chloroplast) [*Ulva* sp..]	gi|108796969|ref|YP_636269.1|	0.0001094	photosynthesis
810	CL646_Contig1_All	chloroplast oxygen-evolving protein 3 [*Chlamydomonas incerta*]	gi|74272689|gb|ABA01140.1|	3.58E-06	photosynthesis
839	CL6565_Contig2_All	photosystem I reaction center subunit II, 20 kDa [*Chlamydomonas reinhardtii*]	gi|412988213|emb|CCO17549.1|	0.0005726	photosynthesis
1410	gi|170293977|gb|ACB13082_1|	ATP synthase beta subunit, partial (plastid) [*Ulva lactuca*]	gi|170293977|gb|ACB13082.1|	0.00137	photosynthesis
1710	Unigene24798_All	predicted protein *[Chlamydomonas reinhardtii*]	gi|159477839|ref|XP_001697016.1|	0.002339	photosynthesis
2256	CL1858_Contig1_All	rieske iron-sulfur subunit of the cytochrome b6f complex, chloroplast precursor [*Chlamydomonas reinhardti*i]	gi|159481438|ref|XP_001698786.1|	8.39E-08	photosynthesis
2965	CL5639_Contig2_All	chloroplast ATP synthase subunit delta precursor [*Coccomyxa subellipsoidea C-169*]	gi|384246118|gb|EIE19609.1|	1.97E-05	photosynthesis
4015	CL2952_Contig1_All	ATP synthase gamma-subunit [*Coccomyxa subellipsoidea* C-169]	gi|384254058|gb|EIE27532.1|	2.89E-06	photosynthesis
1487	gi|145568027|gb|ABP82370_1|	ribulose-1,5-bisphosphate carboxylase/oxygenase large subunit, partial (chloroplast) [*Ulva compressa*]	gi|145568517|gb|ABP82615.1|	0.01299	Carbon fixation in photosynthetic organisms
1640	CL1959_Contig1_All	fructose-1,6-bisphosphate aldolase [*Chlamydomonas reinhardtii*]	gi|159484548|ref|XP_001700318.1|	4.93E-07	Carbon fixation in photosynthetic organisms
2251	gi|145567604|gb|ABP82159_1|	ribulose-1,5-bisphosphate carboxylase/oxygenase large subunit, partial (chloroplast) [Ulva sp.A027488]	gi|145568577|gb|ABP82645.1|	0.02164	Carbon fixation in photosynthetic organisms
3028	gi|300259803|gb|EFJ44027_1|	malate dehydrogenase [*Volvox carteri* f*. nagariensis*]	gi|302846584|ref|XP_002954828.1|	0.0005646	Carbon fixation in photosynthetic organisms
3734	CL1800_Contig3_All	MAP kinase phosphatase 6 [*Chlamydomonas reinhardtii*]	gi|159474472|ref|XP_001695349.1|	3.85E-05	Carbon fixation in photosynthetic organisms
2823	Unigene12037_All	SMAD/FHA domain-containing protein [*Coccomyxa subellipsoidea* C-169]	gi|384246676|gb|EIE20165.1|	1.24E-05	Carotenoids biosynthesis
1308	Unigene18531_All	cytochrome P450 [*Coccomyxa subellipsoidea* C-169]	gi|384250461|gb|EIE23940.1|	0.0002477	zea biosynthesis
2494	CL3922_Contig1_All	prenylated rab acceptor family protein [*Chlamydomonas reinhardtii*]	gi|159463304|ref|XP_001689882.1|	0.002142	Brassinosteroid biosynthesis
786	Unigene5919_All	glutathione S-transferase [*Coccomyxa* sp.]	gi|1150788|gb|AAC50036.1|	0.0007462	Glutathione metabolism
978	Unigene163_All	glutathione S-transferase [*Coccomyxa subellipsoidea* C-169]	gi|384253101|gb|EIE26576.1|	5.11E-07	Glutathione metabolism
1426	Unigene12106_All	glutathione S-transferase [*Coccomyxa subellipsoidea* C-169]	gi|384253101|gb|EIE26576.1|	7.65E-06	Glutathione metabolism
1705	CL52_Contig1_All	glutathione S-transferase [*Chlamydomonas reinhardtii*]	gi|159465645|ref|XP_001691033.1|	0.003677	Glutathione metabolism
1919	CL4568_Contig1_All	glutathione S-transferase [*Chlamydomonas reinhardtii*]	gi|159463928|ref|XP_001690194.1|	0.005224	Glutathione metabolism
2393	CL6567_Contig1_All	chloroplast ascorbate peroxidase [*Chlamydomonas* sp.]	gi|384245236|gb|EIE18731.1|	1.10E-06	Glutathione metabolism
3282	CL2017_Contig1_All	GPX1b [*Chlorella* sp.]	gi|385258209|gb|AFI55002.1|	3.70E-05	Glutathione metabolism
3425	Unigene12312_All	isomerase [*Chlamydomonas reinhardtii*]	gi|159474628|ref|XP_001695427.1|	1.72E-07	Glutathione metabolism
3579	CL1055_Contig7_All	glucose-6-phosphate 1-dehydrogenase 2, chloroplastic-like isoform 2 [*Brachypodium distachyon*]	gi|357168050|ref|XP_003581458.1|	0.001315	Glutathione metabolism
4134	CL2243_Contig1_All	Glutathione S-transferase [*Ectocarpus siliculosus*]	gi|298712930|emb|CBJ26832.1|	3.60E-05	Glutathione metabolism
3025	CL5370_Contig1_All	adenylate kinase [*Coccomyxa subellipsoidea* C-169]	gi|384247647|gb|EIE21133.1|	2.98E-05	Purine metabolism
376	Unigene20819_All	F1F0 ATP synthase gamma subunit [*Coccomyxa subellipsoidea* C-169]	gi|384251996|gb|EIE25473.1|	5.80E-06	Oxidative phosphorylation
388	CL4038_Contig3_All	PREDICTED: cytochrome c1–1, heme protein, mitochondrial-like [*Glycine max*]	gi|356571411|ref|XP_003553870.1|	4.12E-05	Oxidative phosphorylation
421	Unigene8008_All	hypothetical protein NADH dehydrogenase[ubiquinone] [*Chlorella variabilis*]	gi|307104659|gb|EFN52912.1|	0.001728	Oxidative phosphorylation
643	CL1383_Contig2_All	NADH-quinone oxidoreductase, partial [*Coccomyxa subellipsoidea* C-169]	gi|384248569|gb|EIE22053.1|	0.001639	Oxidative phosphorylation
1747	Unigene28169_All	NADH dehydrogenase [ubiquinone] [*Coccomyxa subellipsoidea* C-169]	gi|307104198|gb|EFN52453.1|	0.000603	Oxidative phosphorylation
2429	Unigene16018_All	NADH:ubiquinone oxidoreductase 11 kDa subunit [*Chlamydomonas reinhardtii*]	gi|159475537|ref|XP_001695875.1|	0.0003178	Oxidative phosphorylation
2124	CL1257_Contig3_All	calciumdependent protein 4 [*Ectocarpus siliculosus*]	gi|325189489|emb|CCA23977.1|	0.0007071	Ca^2+^ binding protein
2881	CL25_Contig3_All	EF-hand [*Coccomyxa subellipsoidea* C-169]	gi|384250284|gb|EIE23764.1|	0.0008694	Ca^2+^ binding protein
4102	CL6733_Contig1_All	Calcium dependent protein 4 [*Ectocarpus siliculosus*]	gi|323454023|gb|EGB09894.1|	0.004934	Ca^2+^ binding protein
4225	gi|158284273|gb|EDP10023_1|	Caltractin [*Chlamydomonas reinhardtii*]	gi|159464110|ref|XP_001690285.1|	0.01586	Ca^2+^ binding protein
3021	gi|158282407|gb|EDP08159_1|	EDS1[*Chlamydomonas reinhardtii*]	gi|159476166|ref|XP_001696182.1|	0	Plant-pathogen interaction

**Figure 5:**
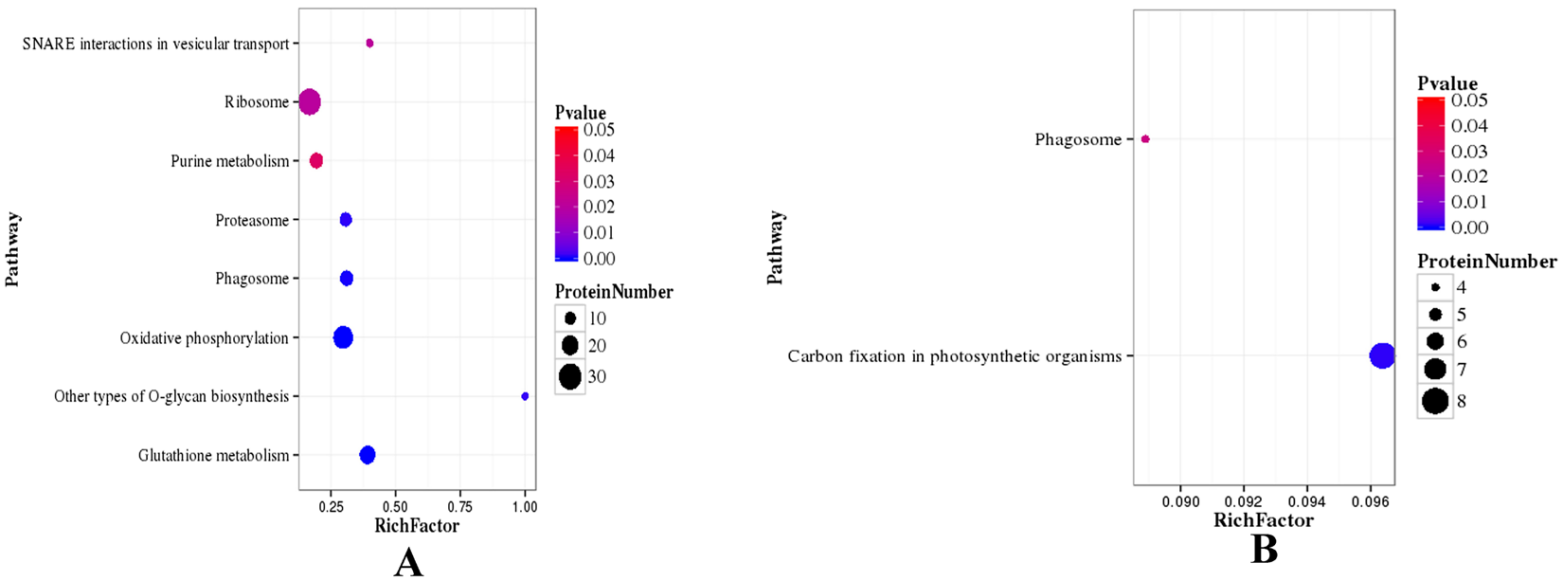
Pathway enrichment statistical scatter plot of DEPs. (**A**) UpSHT vs UpHT down-regulated proteins; (**B**) UpSHT VS UpHT up-regulated proteins; The vertical axis represents the name of the pathway; the horizontal axis represents the pathway corresponding rich factor. The rich factor refers to the ratio of the number of differentially expressed genes in the pathway and the number of all annotated genes in the pathway. Higher rich factors indicate greater degrees of enrichment. Q values are often completed after multiple hypothesis testing with corrected *P* value values ranging from 0 to 0.05. The closer they are to zero, the more significant the enrichment.

Using the Blast2GO program, 429 DEPs were mapped to KEGG data under UpSHT compared with UpHT. The pathway enrichment analysis showed that the most significantly enriched pathways among the 77 up-regulated proteins were carbon fixation in photosynthetic organisms (8). The down-regulated proteins were enriched in glutathione metabolism (18), oxidative phosphorylation (25), phagosome (14), proteasome (12), ribosome (30), other types of o-glycan biosynthesis (3), purine metabolism (14) and SNARE interactions in vesicular transport (4) pathways (Fig. 5A,B and Table 3). At present, under UpHT treatment, glutathione S-transferase, heat shock proteins, MnSOD and ubiquitin-related proteins were up-regulated (data not shown). However, in UpSHT compared with UpHT, glutathione S-transferase, heat shock protein and antioxidant activity-related proteins partion were down-regulated (Table 2). The results indicated that upon high-temperature stimulus, *U. prolifera* induces defense mechanisms, and the expression of heat shock proteins and antioxidant-associated proteins is increased. However, SA addition alleviated the high-temperature stimulus, resulting in down-regulation of antioxidant related proteins.

**Table 3:** DEPs mapped to pathways (top20).

#	Pathway	Diff proteins with pathway annotation (429)	All Proteins with pathway annotation (3046)	P value	Pathway ID
1	Oxidative phosphorylation	28 (6.53%)	84 (2.76%)	4.595368e-06	ko00190
2	Glutathione metabolism	19 (4.43%)	46 (1.51%)	4.790152e-06	ko00480
3	Phagosome	18 (4.2%)	45 (1.48%)	1.465223e-05	ko04145
4	Other types of O-glycan biosynthesis	3 (0.7%)	3 (0.1%)	0.002776944	ko00514
5	Proteasome	12 (2.8%)	39 (1.28%)	0.005547541	ko03050
6	Ribosome	34 (7.93%)	179 (5.88%)	0.03682623	ko03010
7	SNARE interactions in vesicular transport	4 (0.93%)	10 (0.33%)	0.04048394	ko04130
8	Pentose phosphate pathway	8 (1.86%)	30 (0.98%)	0.05031079	ko00030
9	Photosynthesis - antenna proteins	8 (1.86%)	31 (1.02%)	0.05982023	ko00196
10	Brassinosteroid biosynthesis	3 (0.7%)	8 (0.26%)	0.09012274	ko00905
11	alpha-Linolenic acid metabolism	4 (0.93%)	13 (0.43%)	0.0979834	ko00592
12	Carbon fixation in photosynthetic organisms	16 (3.73%)	83 (2.72%)	0.1139435	ko00710
13	Arachidonic acid metabolism	4 (0.93%)	14 (0.46%)	0.1226120	ko00590
14	Purine metabolism	14 (3.26%)	72 (2.36%)	0.1264869	ko00230
15	Taurine and hypotaurine metabolism	1 (0.23%)	1 (0.03%)	0.1408404	ko00430
16	Synthesis and degradation of ketone bodies	1 (0.23%)	1 (0.03%)	0.1408404	ko00072
17	Linoleic acid metabolism	2 (0.47%)	5 (0.16%)	0.1480381	ko00591
18	Diterpenoid biosynthesis	2 (0.47%)	5 (0.16%)	0.1480381	ko00904
19	C5-Branched dibasic acid metabolism	2 (0.47%)	5 (0.16%)	0.1480381	ko00660
20	Porphyrin and chlorophyll metabolism	9 (2.1%)	47 (1.54%)	0.2078737	ko00860

### Correlations between transcriptome and proteome data

**Figure 6:**
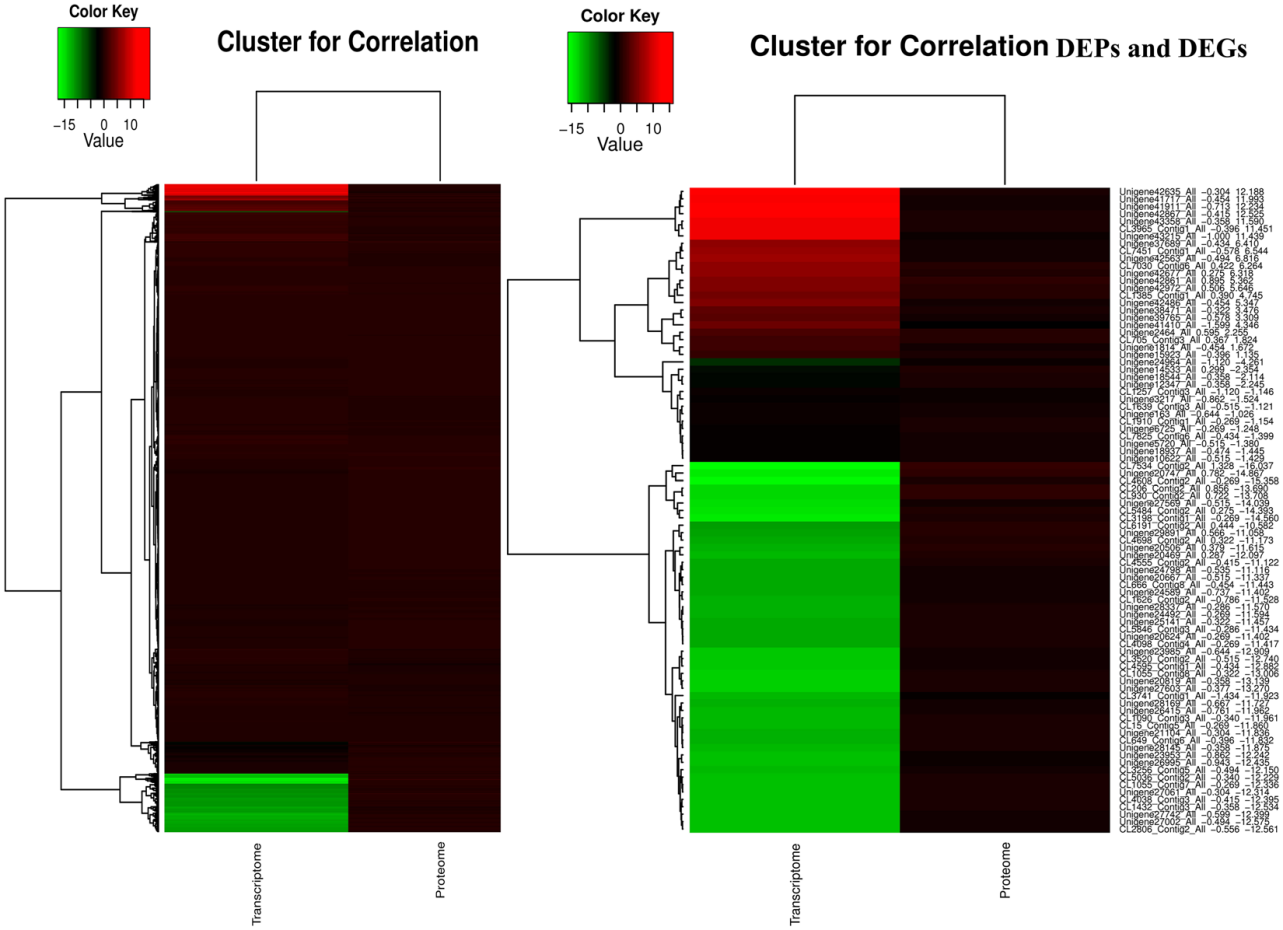
Heatmap analysis to the correlation of all proteins from proteome and transcriptome (**A**) and correlation DEPs from DEPs and DEGs (**B**) on the basis of the relative abundance (log2 (UpSHT/UpHT) of proteome and transcriptome. The small panel with color gradient represents the changes of protein abundance or mRNA abundance from down-regulated (green) to up-regulated (red).

Integration of the proteome and transcriptome of *U. prolifera* was also conducted. Venn diagrams of the correlation numbers for the identification and quantification of the proteome and transcriptome are shown in Supplementary Fig. 3. A one-by-one search found that 3984 (89.55%) of the 4449 identified proteins had corresponding transcripts in the RNA-seq data. In addition, correlations between the 651 DEPs and the 12,296 DEGs were 87 DEPs. Because RNA-seq detection is much more sensitive than proteome determination, the RNA-seq data are likely to delineate the entire figure of the cluster structure. We investigated whether changes in protein levels correlated with changes in the corresponding transcripts. A low correlation between transcript level changes and protein level changes was observed for all quantified proteins. mRNA formation is not always correlated with protein synthesis, particularly for intermediate- and low-abundance mRNAs and proteins^22^. Because a large number of protein-encoding genes are likely to be elicited from the gene clusters, we suspect that transcription and translation of these genes may follow a synchronized mode in response to environmental temperature and hormone regulation^23^. If this hypothesis is correct, the corresponding mRNAs may display similar modes of abundance changes as the corresponding proteins. We therefore conducted quantitative correlation of the proteomes and transcriptomes. We mapped the correlated proteomes and transcriptomes and compared the modes of abundance changes due to SA regulation for both mRNAs and proteins. Heat map analysis of correlation proteins of the transcriptome and proteome and cluster analysis of DEPs and DEGs were conducted (Fig. 6 and Supplementary File 2). In the analysis of the correlation of DEPs and DEGs, 87 DEPs were matched with corresponding DEGs, and 60 DEPs (60/87, 68.96%) were well matched with the tendency of the change in abundance of the mRNA. The fold changes in abundance are listed in Supplementary File 2. The up-regulated correlated DEPs included translation elongation factor-like protein, aspartate aminotransferase, minus strand six-hairpin glycosidase, eukaryotic translation elongation factor 1 alpha, translated and other predicted proteins. A total of 53 correlated DEPs were the down-regulated, including ribosomal protein, polyubiquitin, glutathione S-transferase, component of TRAPP complex, GRIM-19, mitochondria-targeted chaperonin putative, thioredoxin, aspartate aminotransferase, glucose-6-phosphate 1-dehydrogenase 2 and rubisco activase. The corresponding DEGs exhibited the same trends. These results firmly support our hypothesis that significant abundance changes in the correlated DEPs are tightly correlated with the transcriptional activity of the corresponding DEGs. In our study, the correlated DEPs and DEGs were tightly correlated with the transcriptome. However, opposite patterns of DEP and DEG expression were also observed. For example, chloroplast light-harvesting protein CP29 precursor were up-regulated in the proteome analysis, whereas the corresponding DEGs down-regulated. The 17.8 kDa heat shock protein was down-regulated at the translation level but up-regulated at the transcriptome level. In the process of protein synthesis, factors such as codon preference, differences in ribosome concentration, the efficiency of translation of an organism, and the states of tissues and cells may differ.

DEPs may significantly increase or decrease without a change in the corresponding mRNAs. The statistical analysis of protein expression changes in the absence of mRNA expression changes contributed to the elucidation of the biological processes. In the present study, thioredoxin m, thioredoxin-like protein, proteasome regulatory subunit, glutathione S-transferase, thioredoxin peroxidase, thioredoxin x, ubiquinone oxidoreductase, mitochondrial chaperonin HSP10, ubiquinone oxidoreductase 11 kDa and MnSOD were found to be down-regulated, whereas the corresponding mRNAs showed no change. In addition, oxidoreductase, soluble carbonic anhydrase precursor, adenylate kinase, acireductone dioxygenase, ubiquitin carboxyl-terminal hydrolase 1, and six-hairpin glycosidase were significantly increased, whereas their corresponding mRNAs showed no change in abundance. Significant changes in DEGs without changes in the corresponding proteins were also observed. Heat shock protein 90 increased, and ferredoxin-thioredoxin reductase (FTR), NADH:ubiquinone oxidoreductase 24 kDa subunit, calcium-dependent protein kinase, light-harvesting protein of photosystem I, and thioredoxin-like protein decreased, but their corresponding proteins did not change. Although the global correlation result was negative, these correlation sequences provide a new direction to explore the interaction of the transcriptome and proteome. Therefore, the quantitative proteome and transcriptome of *U. prolifera* under SA regulation at high temperature not only provide functional information to explore SA-regulated proteins but also offer mechanistic insights into the regulation of the expression of these proteins. In summary, these results suggest that changes in protein profiles at high temperatures may be controlled at the post-transcriptional level, and thus, changes in mRNA expression provide only limited insight into changes in protein expression.

### Concordance within KEGG biological pathways

**Figure 7:**
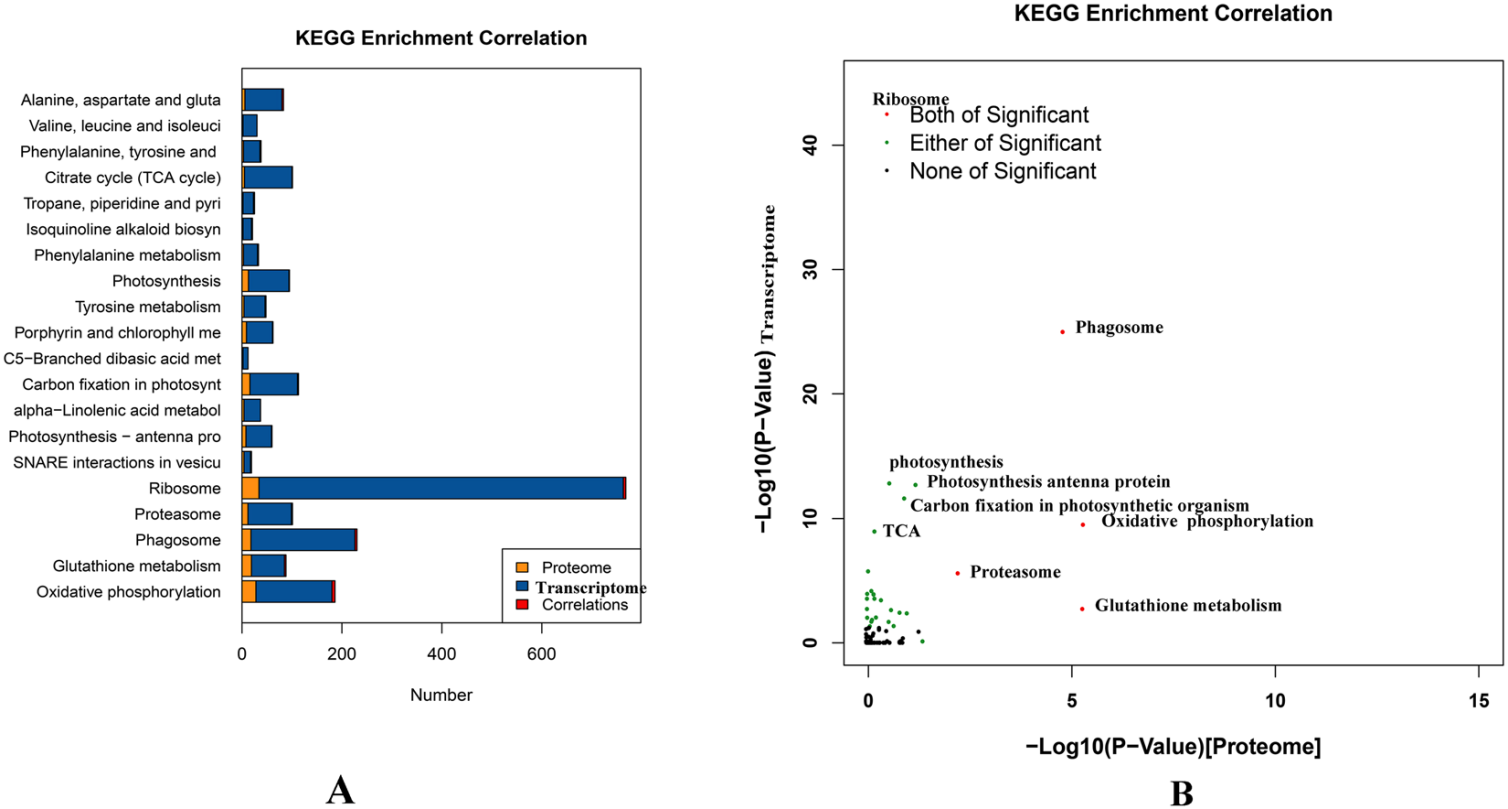
KEGG enrichment correlation proteome and transcriptome. (**A**) Number of KEGG enrichment correlation proteome and transcriptome. (**B**) The overview scatter diagram of KEGG enrichment correlation between the protein level and transcript level of genes.

To obtain an overview of the correlation between the protein and transcript levels of genes within 91 KEGG pathways, the correlation of proteome and transcriptome KEGG enrichment was analyzed (Fig. 7A,B). Ribosome, phagosome, oxidative phosphorylation, glutathione metabolism and proteasome were significant pathways in both the proteome and transcriptome. In addition, genes involved in photosynthesis, carbon fixation, TCA and amino acid metabolism were enriched at the transcriptome level. SNARE interactions in vesicular transport were enriched only at the proteome level. This analysis indicates that proteins involved in metabolic pathways were particularly well controlled at the post-transcriptional level.

### Expression patterns of the protein-protein interaction (PPI) network

Most proteins exert their biological functions by interacting with each other. To uncover functional aspects associated with these proteins, we constructed a PPI network based on data downloaded from the STRING database. Only protein pairs with a confidence score >0.4 were used to construct the PPI network. For the UpSHT vs UpHT protein correlation with the transcriptome, we choose 278 proteins involved in response to stimulus, photosynthesis, carbon fixation in photosynthetic organisms, antioxidant activity, heat shock protein, and plant signal transduction to construct the PPI.

**Figure 8:**
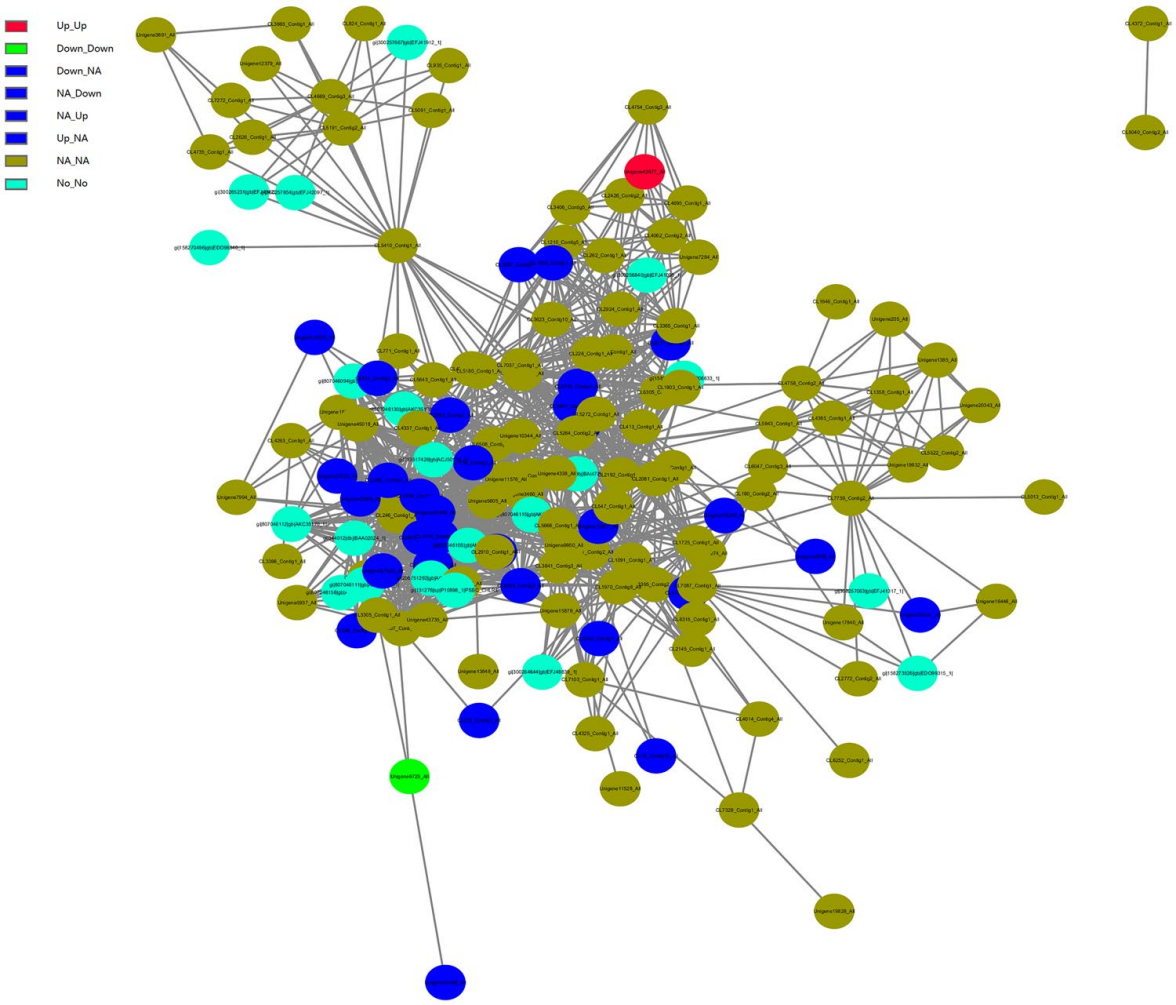
The protein-protein(portion) interaction network of *U. prolifera* under UpSHT compared with UpHT condition. These PPI interactions with a combined score larger than 0.4 in the STRING database were extracted to build the network. The gene with different regulatory pattern in protein/transcript level were marked as different color as follows: up/up, Red; down/down, Green; up/NA, down/NA, NA/up, NA/down, Blue; NA/NA, yellow green; No/No, navy.

Based on the differential expression patterns at the transcript and protein levels in UpSHT compared to UpHT, these nodes in the PPI network were divided into seven groups (Fig. 8). We described five groups with differential expression of both the transcript and protein level. In group 1 and group 2, aspartate aminotransferase were up-regulated at the transcript level and protein level. In addition, polyubiquitin were down-regulated at the transcript level and protein level. Group 3 and group 4 contained 20 genes with up-regulated or down-regulated expression at the protein level and no changes at the transcript level in UpSHT compared with UpHT. These genes were mainly enriched in response to stimulus,antioxidant activity, photosynthesis, carbon fixation in photosynthetic organisms, carotenoid biosynthesis and zeatin biosynthesis. Group 5 contained 11 genes with up-regulated or down-regulated expression at the transcript level but no difference in expression at the proteome level. These genes were primarily enriched in response to stimulus, photosynthesis, and carbon fixation in photosynthetic organisms.

### Transcriptome and proteome correlation expression analysis of antioxidant activity

Reactive oxygen species (ROS) refer to free radicals, including hydrogen peroxide (H_2_O_2_), singlet oxygen (O_2_^−^), and hydroxylradicals (OH^−^). ROS are natural by-products of normal oxygen metabolism, drug metabolism, and other intracellular redox reactions. Excessive amounts of ROS can damage macromolecules and cell membranes. To removetoxic ROS, aerobic organisms have developed a number of antioxidant systems that serve as protective mechanisms, namely superoxide dismutases (SOD), catalases, peroxidases, thioredoxin, and glutathione^24^. A previous study indicated that abiotic stresses such as high temperature, low temperature, and drought induce plant defense mechanisms, including the expression of antioxidant enzymes to regulate their adaptation mechanisms^25^. In the present study, we determined that the transcript abundance of 31 antioxidant genes exhibited highly dynamic changes in response to the UpSHT condition. Transcriptome analysis indicated that catalase, ascorbate peroxidase, glutathione S-transferase and glutathione reductase were down-regulated, while up-regulated genes were dominated by four peroxiredoxin, FeSOD, partion catalase and MnSOD. In the correlation analysis of the transcriptome and proteome, 11 correlated proteins were involved in the antioxidant activity (Table 4). Six DEPs, glutathione S-transferase, polyubiquitin, thioredoxin peroxidase (ISS), GPX1b, MnSOD and L-ascorbate peroxidase were down-regulated, but only polyubiquitin and glutathione S-transferase corresponded to DEGs; the other corresponding genes showed no significant changes. In the analysis, we found that a gene may encode multiple transcripts and peptides, and the expression of these transcripts and peptide segments may different. Different transcripts of the same gene may perform different functions but have similar physiological performance. The results indicated that high temperatures induced the expression of antioxidant genes, but the addition of SA alleviated the stimulation, leading to partial down-regulation of antioxidant genes. This process is consistent with the results reported for the brown alga *Ectocarpus siliculosus* ^26^.

**Table 4:** Correlation antioxidant and heat shock proteins list.

NO.	Protein ID	Protein Description	DEPs	Gene ID	DEGs	molecular function
454	CL232_Contig1_All	401 985 minus strand hypothetical protein [*Chlorella variabilis*]	—	CL232.Contig1_All	NA	antioxidant activity
695	Unigene6725_All	53 280 minus strand polyubiquitin [*Aureococcus anophagefferens*]	—	Unigene6725_All	—	antioxidant activity
1284	CL547_Contig1_All	1 1194 catalase [*Ulva fasciata*]	NA	CL547.Contig1_All	—	antioxidant activity
1321	Unigene13607_All	thioredoxinperoxidase[*Ostreococcus tauri*]	—	Unigene13607_All	NA	antioxidant activity
1323	CL514_Contig1_All	1 408 glutathione reductase [*Ulva fasciata*]	NA	CL514.Contig1_All	NA	antioxidant activity
1365	Unigene18274_All	430 1071 heme peroxidase [*Coccomyxa subellipsoidea* C-169]	NA	Unigene18274_All	NA	antioxidant activity
2070	CL1091_Contig1_All	134 1552 minus strand glutathione reductase [*Chlamydomonas* sp_ ICE-L]	NA	CL1091.Contig1_All	NA	antioxidant activity
2539	CL2145_Contig1_All	NADPH dependent thioredoxin reductase [*Volvox carteri* f.*nagariensis*]	NA	CL2145.Contig1_All	NA	antioxidant activity
3282	CL2017_Contig1_All	222 668 minus strand GPX1b [*Chlorella* sp_ NJ-18]	—	CL2017.Contig1_All	NA	antioxidant activity
3388	Unigene9950_All	thioredoxin dependent peroxidase [*Chlamydomonas reinhardtii*]	NA	Unigene9950_All	NA	antioxidant activity
4113	Unigene7941_All	Predicted protein [*Ostreococcus lucimarinus* CCE9901]	NA	Unigene7941_All	NA	antioxidant activity
2681	CL6691_Contig2_All	MnSOD [*U. prolifera*]	—	CL6691.Contig2_All	NA	antioxidant activity
202	Unigene40313_All	Heat Shock Protein 90, cytosolic [*Ostreococcus lucimarinus* CCE9901]	NA	Unigene40313_All	+	HSP
739	CL1725_Contig1_All	heat shock protein 70 [*U. prolifera*]	NA	CL1725.Contig1_All	NA	HSP
848	Unigene1814_All	17_8 kDa class I heat shock protein [*Arabidopsis thaliana*]	—	Unigene1814_All	+	HSP
1066	CL5126_Contig3_All	small heat shock protein; heat shock protein 20 [*Ectocarpus siliculosus*]	NA	CL5126.Contig3_All	NA	HSP
1236	Unigene47599_All	heat shock protein 70, partial [*Spumella uniguttata*]	NA	Unigene47599_All	NA	HSP
1782	CL2920_Contig1_All	heat shock protein Hsp70E [*Volvox carteri* f*. nagariensis*]	NA	CL2920.Contig1_All	NA	HSP

### Transcriptome and proteome correlation expression analysis of plant hormone biosynthesis and signal transduction related genes

Studies have investigated the effects of different phytohormones on growth and development in plants. Some investigations have focused on certain stress responses mediated by phytohormones^27–29^, whereas other studies have focused on the effects of regulatory elements on hormones in signaling pathways^30^ and the relationships between different types of phytohormones^31^. In the present study, the expression pattern analysis showed that SA application changed the expression of most transcripts encoding key enzymes involved in plant hormone signal transduction and zeatin biosynthesis at mRNA level and protein level. ARFs (which bind specifically to TGTCTC auxin response elements (AuxRE) in the promoters of these genes and function in combination with Aux/IAA (auxin/indole acetic acid) repressors) dimerize with ARF activators in an auxin-regulated manner^32, 33^. ARFs were down-regulated at the transcript level, indicating that SA affected the expression of ARFs, auxin signal transduction, and consequently, the growth of *U. prolifera*. Abscisic acid (ABA) serves as an endogenous messenger in biotic and abiotic stress responses in plants, and drought and high salinity result in strong increases in ABA levels in plants, accompanied by a major change in gene expression and adaptive physiological responses^34^. ABA is formed by the oxidative cleavage of carotenoids. The expression of ABA1 (zeaxanthin epoxidase) and ABA-responsive element binding factors (ABFs, CL1484.Contig2_All) involved in the signaling pathway of ABA were down-regulated at the mRNA level. However, xanthoxin dehydrogenase (ABA2, Unigene42538_All) was up-regulated. These results indicated that ABA biosynthesis and signal transduction decreased. In the cytokinin signaling pathway, decreases in the downstream elements histidine phosphotransfer proteins (AHPs, CL7329.Contig2_All) and the two-component response regulator ARR-A family and cytokinin trans-hydroxylase were observed. In addition, BRI1 a cell-surface receptor for BRs that plays key regulatory roles in the BR signal transduction pathway and serine/threonine-protein phosphatase (BSU1) decreased. As a key negative regulator, Jasmonate ZIM-domain (JAZ) plays a central role in the signal transduction of JA. The observed down-regulation of JAZ (Unigene27760_All) suggests an enhancement of JA signal transduction. In the ethylene signaling pathway, CTR1(Serine/threonine protein kinase) is key negative regulator, 12 unigenes encode CTR1, 4 unigenes were up-regulated, and 8 unigenes were down-regulated. Mitogen-activated protein kinase 6 (MAPK6, Unigene37470_All) was up-regulated, and Unigene6149_All was down-regulated.

In the integrated transcriptome and proteome data, a broad survey of plant hormone signal transduction resulted in the identification of transcripts and proteins corresponding to a substantial number of key enzymes. Approximately 118 proteins or mRNAs were mapped to plant hormone signal transduction, and 14 key enzymes were identified. In zeatin biosynthesis, the expression of cytokinin trans-hydroxylase protein (CYP735A, Unigene18531_All) was decreased at the mRNA and protein levels. In brassinosteroid (BR) biosynthesis, three significant DEPs mapped to BR synthesis decreased: 90B/724B(gi|158273526|gb|EDO99315_1|_Protein, CL3922_Contig1_All_Protein (90C1D1), and Unigene18531_All_Protein(735A). In the plant signal transduction process, 7 key proteins correlated with their mRNAs, including CRE1, AHP, B-ARR, PP2C, SnRK2, CTR1, and BSU1, were identified at the mRNA and protein levels, but the expression of these proteins was not significant. It may be inferred that SA enhanced the effects of high temperature on the expression of key regulated enzymes in plant hormone synthesis and signal transduction. The expression of key genes in the signal transduction pathway of carotenoid biosynthesis, the ABA signaling pathway, the zeatin biosynthesis pathway, and cytokinin were reduced, whereas the expression of those in the JA signaling pathway increased. These mechanisms increase plant resistance, and SA is added to alleviate high-temperature-related stress. Thus, this result is consistent with studies of *Populus euphratica* ^35^ and and *Poncirus trifoliata*, but differs from an equivalent analysis of *Lilium lancifolium* ^36^. Phytohormone signal pathway regulation plays important roles in SA regulation in response to high-temperature stresses, but the specific mechanisms by which phytohormone mediation signaling is involved in the SA responses of *U. prolifera* remain to be determined.

### Transcriptome and proteome correlation expression analysis of heat shock protein

Heat shock proteins (HSP) and antioxidant activity play vital role in protecting against stress by re-establishing normal protein conformation and maintaining cellular homeostasis^37^. In higher plants, the signal response to high-temperature stress involves a reduction in the synthesis of normal proteins and is accompanied by accelerated expression of heat-responsive genes and HSPs. In most organisms, the up-regulation of genes encoding HSPs and other chaperones has been observed under abiotic stress conditions. In our study, under high temperature condition without SA, the expression of HSPs were induced (data not shown). While a total of 42 unigenes encoding HSPs were differentially expressed at the mRNA level under UpSHT compare with UpHT. In addition, HSP70, HSP90, HSP60 and HSP10 were all significantly differentially expressed in the SA treatment under high-temperature conditions at the transcriptome level. HSP60 and HSP10 were down-regulated, whereas HSP70 and HSP90 did not show consistent changes: they were partially up-regulated and partially down-regulated. In the proteome analysis, changes in 16 unigenes encoding HSP90, HSP70 and HSP17 were correlated with the changes at the mRNA level. By contrast, Unigene1814_All encode HSP17 was significantly down-regulated at the protein level but up-regulated at the mRNA level. The DEG of Unigene40313_All encoding HSP90 was up-regulated, whereas no significant change was observed at the protein level (Table 4). The total balance of HSP proteins in algae may be a key factor in stress tolerance of the intertidal environment.

### Transcriptome and proteome correlation expression analysis of photosynthesis-related proteins

Photosynthesis is one of the systems that are most sensitive to high-temperature stress. Changes in environmental temperature are primarily reflected by photosynthesis, which triggers a response aimed at attaining the best possible performance under the new conditions. Consequently, a balance is sought between the energy of absorbed light, carbon assimilation, and consumption in metabolic sinks. Several studies have shown that high-temperature stress can significantly inhibit photosynthesis^38, 39^. The process of photosynthesis is initiated by the absorption of light. In green algae, light-harvesting chlorophyll protein complexes are the major light-harvesting complexes. These complexes comprise photosystem α (light-harvesting complex α, photosynthetic reaction center), photosystem I (LHCI and photosynthetic reaction center) and linker polypeptides. The light-harvesting complex is a complex of subunit proteins that may be part of a larger supercomplex of the photosystem, the functional unit in photosynthesis and used by plants and green algae to collect more of the incoming light than would be captured by the photosynthetic reaction center alone. In the present study, the LHCI subunits lhca1, lhca2 and lhca5 were down-regulated at both the transcriptome and proteome levels, and lhca3 and lhca4 were down-regulated at the transcriptome level and proteome level, respectively. In addition, the LHCα subunits lhcb1, lhcb2 and lhcb3 were also down-regulated at the protein and mRNA levels. Lhcb5 was down-regulated at the mRNA level. Down-regulation of the LHC would significantly influence light absorbance and energy transfer in *U. prolifera*. In seaweeds, carbonic anhydrase plays key roles in the CO_2_-concentrating mechanism (CCM) by accelerating the conversion of the external HCO_3_− pool into CO_2_ or by mobilizing the internal pool of HCO_3_− to supply the carboxylation reaction of RuBisCo^40, 41^. RuBisCo catalyzes the first major step of carbon fixation in photosynthesis. The affinity of RuBisCo for CO_2_ decreases with increasing temperature. RuBisCo activase is required to maintain RuBisCo in the activated state. In the present study, although the level of carbonic anhydrase precursor was up-regulated at the mRNA level, the level of expression of RuBisCo activase was down-regulated at the protein and mRNA levels. The diminished capacity of RuBisCo and its low affinity for CO_2_ clearly indicated that carbon fixation or assimilation was highly inhibited when *U. prolifera* were exposed to SA under high-temperature stress, may be RuBisCo is not the only limited factor for photosynthesis. Decreases in proteins participating in photosynthetic electron and energy transfer, such as cytochrome b6-f complex iron-sulfur subunit, ferredoxin-NADP^+^ reductase, F-type H^+^-transporting ATPase subunit, fructose-1,6-bisphosphatase, and glyceraldehyde 3-phosphate dehydrogenase, were also observed. Thus, the down-regulated expression of these proteins further suggests that photosynthesis in *U. prolifera* is inhibited by SA regulation under high-temperature stress, However, the increase of the expression of carbonic anhydrase precursor plays an important role in the accelerate to photosynthesis balance adjustment.

### Transcriptome and proteome correlation expression analysis of Ca^2+^-binding protein

**Figure 9:**
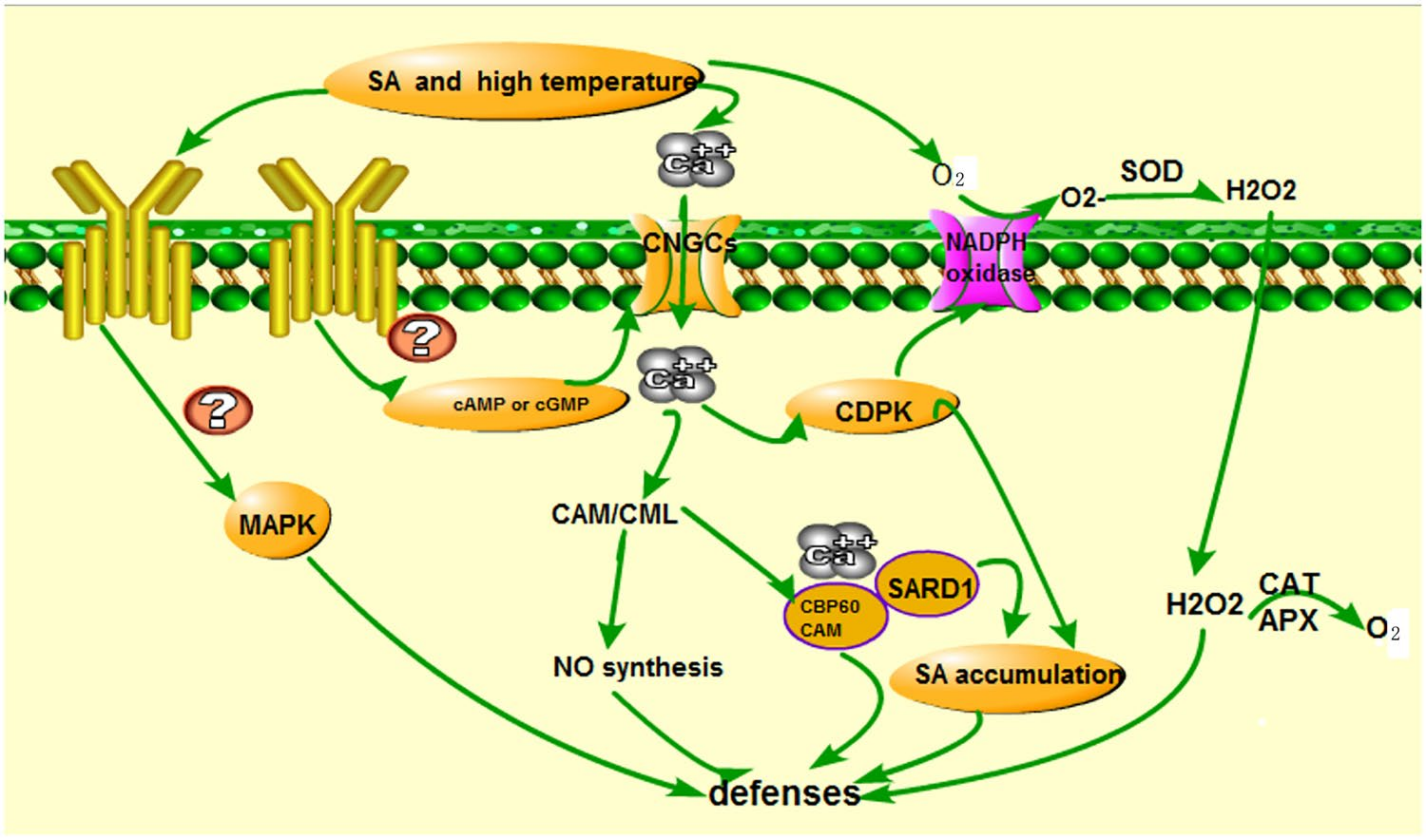
Model of possible mechanisms of SA regulated high temperature in the defense signaling transduction in *U. prolifera*.

Ca^2+^-dependent signalling processes enable plants to perceive and respond to diverse environmental stressors, such as osmotic stress and high temperature^42–44^. Cyclic nucleotide-gated channels (CNGCs) are Ca^2+^-permeable cation transport channels, which are present in both animal and plant systems. They have been implicated in the uptake of both essential and toxic cations, Ca^2+^ signaling, pathogen defense, and thermotolerance in plants^45, 46^. Ca^2+^ elevation in the cytosol is an essential early event during pathogen response signaling and environment stress cascades. However, the specific ion channels involved in Ca^2+^ influx into plant cells, and how Ca^2+^ signals are initiated and regulate downstream events during pathogen defense responses, are at present unclear. CNGCs provide a pathway for Ca^2+^ conductance across the plasma membrane (PM) and facilitate cytosolic Ca^2+^ elevation in response to stimuls. Recent studies indicate that the recognition of environment stimuls results in cyclic nucleotide production and the activation of CNGCs, which leads to downstream generation of pivotal signaling molecules (such as NO). Calmodulins (CaMs) and CaM-like proteins (CMLs) are also involved in this signaling, functioning as Ca^2+^ sensors and mediating the synthesis of NO during the plant pathogen response signaling cascade. The previous study indicated pathogen stimuls could actived Ca^2+^ signal pathway and obtained hypersensitive response^45^. In addition, CaM binding protein CBP60g contributes to MAMP-induced SA accumulation and is involved in disease resistance against *Pseudomonas syringae* in *Arabidopsis thaliana* ^47^. In our previous study, the expression of HSP90, CDPK, and EDS1 were up-regulated in *U. prolifera* under high temperature condition, Ca^2+^ signal play a key role in regulating high temperature in *U. prolifera*. It could be infer that high temeprature stimuls lead to Ca^2+^ signal enchanced and CBP60 when activated by CAM binding, positively regulated signaling leading to SA accumulation and defense gene expression. In the present study, the expression of CNGCs(CL4094.Contig1_All) were down-regulated. 24 unigene encoded CDPK, of which 13 unigene up-regulated. 51 unigene encoded CAMCML, 39 unigene up-regulated. However, at protein level, the expression of CALM, CAMCML, EDS1, CDPK were down-regulated. CNGC, CAMCML and CDPK expression declined. Ca^2+^ and CALM conjugate Ca^2+^-CAM decreased result in regulating downstream signal component NO and H_2_O_2_ accumlation decreased, which are essential for the development hypersensitive response. In addition, CDPK can activate H_2_O_2_ accumulation through regulated NADPH oxidase (Fig. 9). In the biological index measured indicated SOD and CAT activity increased, MDA content declined result in H_2_O_2_ content decreased. The results indicted that SA addition alleviated high temperature stress, ROS signal declined, inhibited Ca^2+^ signaling and EDS1 expression. In conclusion, Ca^2+^-signal play the important role in SA regulated high temperature stress in *U. prolifera*.

### qPCR to verify the reliability of the transcriptome

To verify the reliability of the transcriptome data, the expression of 14 unigenes were investigated. 86% of unigenes were consistent with the available transcriptome data (Supplementary Table 1). The results showed that the expression level of 12 unigene genes was consistent with the data of the obtained transcriptome. The expression of peroxiredoxin5 (Unigene25055_All) were down-regulated in the transcriptome data, but peroxiredoxin5 was up-regulated in qPCR. NADPH-oxidase (Unigene22247_All) were down-regulated in the transcriptome, but not significant reduced in qPCR.

### Biological index and photosynthesis index

SOD belongs to the metalloenzyme family, which played an important role in preventing oxidation of biological molecules, and could protect plant tissues from an elevated concentration of O_2_^−^ produced by a number of environmental stresses. SODs catalyzed O_2_^−^+2H^+^ →O_2_ +H_2_O_2_. In addition, APX and CAT catalyzed H_2_O_2_→O_2_+H_2_O. In the present study, the SOD, CAT, APX activity, and MDA content have been detected. The result indicated that a certain concentration of salicylic acid could alleviate high temperature pressureand improve SOD, CAT, APX activities at early time. MDA content (indicating that the degree of membrane lipid) decreased, and enhanced the ability of the *U. prolifera* to scavenge free radicals(Supplementary Figs 4, 5, 6 and 7). SA 0.1mmol/L is the best concentration. So in the present study, we choose the SA 0.1mmol/L as the teratment concentration.

In this paper, we measured the responses of photosynthesis of *U. prolifera* under UpSHT, UpHT and UpC (25 °C). The results suggested that PSII photosynthesis were significantly reduced at the onset of 3h under UpHT compared to UpC. These reductions were reflected by decreases in the maximum quantum yield (Fv/F m), the effective photosynthetic quantum yield (Fv′/Fm′), the respiration rate and the photosynthetic oxygen evolution rate. It indicated that high temperature stimuls damage PSα and reduce the original light energy conversion efficiency, photosynthesis reaction process is restrained. At the previous report, PSII and nitrogen assimilation responses to desiccation of *U. prolifera* were significantly reduced at the onset of desiccation. These reductions were reflected by decreases in the maximum quantum yield (Fv/Fm), effective photochemical quantum yield (Fv′/Fm′), light utilization efficiency (α)^48^. Our PSII photosynthetic performance results for *U. prolifera* were consistent with the declining trend during high temperature. However, the accumulation of pigment content, the value of Fv′/Fm′, Fv/Fm, relative electron transport rates (rETR) increased. The respiration rate and the photosynthetic oxygen evolution rate decreased under UpSHT compare to UpHT conditions, but not significant (Supplementary Figs 8, 9, 10 and Supplementary Table 2).

## Conclusions

We compiled a comprehensive data set of protein and transcript expression changes that occur in *U*. prolifera grown in SA treatment at high temperature. We demonstrated that that post-transcriptional gene regulation influenced different biological pathways and secondary metabolite genes. On the basis of these findings, we proposed that some proteins related to electron transport chain of photosynthetic, antioxidant enzymes, HSPs and hormone signal transduction, other stress response proteins, Ca^2+^ signaling may play key roles in enhancing *U. prolifera* adaptation to SA regulation from heat stress. These results provide a better understanding of the proteins involved in, and mechanisms of thermotolerance in *U. prolifera*.

## Materials and Methods

### Algal materials

*U. prolifera* samples were collected from the intertidal zone of the eastern gulf of the East China Sea, Xiangshan, Ningbo, China (121.82424 E, 29.552086 N). The algae were washed, fouling organisms and mixed algae were brushed away, disinfected with 0.2 % KI for 10 min, and then flushed with sterile seawater. The *U. prolifera* samples were cultured in Provasoli seawater medium^49^ with 200 µg/mL ampicillin at 25 °C with 40 μmol photons m^−2^s^−1^ irradiance provided by cool-white fluorescent lamps with a photoperiod of 12:12 (light/dark) in a biochemical incubator. In order to obtain relatively sterile *U. prolifera* materials, *U. prolifera* were disinfected and transfered in fresh Provasoli seawater medium every 7 days, and continuous cultured for 35 d at 25 °C. Subsequently, *U. prolifera* were treated with 0.1 mmol·L^−1^ SA and transferred to a high-temperature 35 °C growth chamber with the same irradiance and photoperiod (denoted UpSHT), *U. prolifera* samples were cultured at 35 °C as the control (denoted UpHT), and each treatment has three biological repeats. The gametophyte thalli were harvested at 1h and 3 h after being transferred to the 35 °C chamber, the gametophyte thalli were snap frozen with liquid nitrogen and maintained at −80 °C until RNA and protein extraction.

### High–throughput RNA sequencing and data processing

The total RNA isolated from three biological repeats sample was mixed. RNA extraction was performed according to the manufacturer’s instructions of cetyltrimethyl ammonium bromide-polyvinyl pyrrolidone (CTAB-PVP) method. The total RNA was checked for quality and quantity using an agilent 2100 bioanalyzer. Briefly, total RNA (about 200ng) was enriched by oligo(dT) magnetic beads and oligo(dT) beads were used as primer to synthesize the first and second strand cDNA. After checking and quantifying the DNA, we mixed the multiplexed DNA libraries with normalized 10nM concentration in equal volumes. The library was then sequenced using an Illumina HiSeq™ 2000 platform^50^. Raw reads produced from sequencing machines contain dirty reads, which have been uploaded to the NCBI Sequence Read Archive (Bioproject: PRJNA321448 (SRP074997). The clean reads were acquired by removing low-quality reads and were used for denovo transcriptome assembly. Trinity software were used to assemble the transcriptomes according to the report by Grabherr *et al*.^51^. High-quality reads were assembled into contigs, transcripts, and unigenes using Trinity (http://trinityrnaseq.sourceforge.net/)^51, 52^. The functional annotation of all-unigenes was performed by blast (http://blast.ncbi.nlm.nih.gov/Blast.cgi) against NCBI, Nt, Kyoto Encyclopedia of Gene Genomes (KEGG) database, Cluster of Orthologous Groups (COG) database, Nr database, and Swiss-Prot database with an e-value cut-off of 1e^−5^.

The reads per kilobase per million mapped reads (RPKM) were used to quantify the gene expression. RPKM values were normalized, genes with significantly different expression were determined by FDR ≤ 0.001 AND |log2(UpSHT/UpHT)| ≥ 1. Functional annotation and classifcation of genes for the DEGs was conducted using the Blast 2 GO program (http://www.blast2go.com/b2ghome)^53^ and KEGG pathway analysis and Kyoto Encyclopedia of Genes and Genomes (KEGG) (http://www.genome.jp/kegg-bin/search_pathway) were performed. The clustering of the heat map was conducted using Cluster 3.0 and treeview.

### Protein preparation and iTRAQ labeling

Protein samples from *U. prolifera* were prepared denote UpHT and UpSHT, each treatment has three biological repeats. Protein sequencing were conducted by 8-plex isobaric tags for relative and absolute quantitation. Protein were extracted using the following method. Briefly, *U. prolifera* samples were disrupted in lysis buffer (including 0.1M·L^−1^ Tris-HCl, 1.4 M·L^−1^ NaCl, 0.02 M·L^−1^Na_2_EDTA, 2 % CTAB, 0.1 % DIECA, 2 % PVP K-30,0.2 % β-Mercaptoethanol adjust pH to 8.0) with enzyme inhibitors (Pheylmethylsulfonyl fluoride, PMSF) by tissue lyser machine and sonicated on ice. The expected proteins were extracted after centrifugation at 25,000 g for 20 mins. The supernatant was carefully removed and mixed with 5 volume of cold acetone, stored at −20 °C for overnight. The mixture was centrifuged again. Dissolve the pellets with lysis buffer. Add 10mM DTT (dithiothreitol) to the solution and keep it at 56 °C for 1 h to reduce the disulfide bond of peptides. Add 55 mM IAM(Iodacetamide) to solution and keep it in a dark room for 45 mins. Add 5 volume of chilled acetone into the solution and keep it at −20 °C for 2 h. Centrifuge the solution again. Dissolve the pellet with lysis buffer to get protein solution. Then, determined protein quality by SDS-PAGE. Each 100 μg of protein was digested in trypsin solution (1:10) and incubated at 37 °C for 4 h. The digested peptides were labelled using iTRAQ reagents according to the manufacturer’s instructions (Applied Biosystems, Foster City, CA, USA). The peptides from UpHT and UpSHT were labelled with 115, 116, 117, 118, 119, and 121 iTRAQ reagents, respectively. Peptide were separated and analyzed by LC-ESI-MS/MS. To decrease the complexity of the labelled pepides, the mixture was separated by strong cation exchange chromatography using a Shimadzu HPLC system (LC-20AB; Shimadzu, Kyoto, Japan). For reverse phase chromatography, the Shimadzu LC-20AB HPLC system were used, the digested peptide was reconstituted with solvent A (5 % acetonitrile, 95 % H_2_O, adjust pH to 9.8 with ammonia) to 2 mL and loaded onto a 4.6×250 mm Gemini C18 column containing 5-μm particles (Phenomenex). The peptides are eluted at a flow rate of 1 mL·min^−1^ with a gradient of 5 % solvent B (5 % H_2_O, 95% acetonitrile, adjust pH to 9.8 with ammonia) for 10 min, 5–35 % solvent B for 40 min, 35–95 % solvent B for 1 min. The system is then maintained in 95 % solvent B for 3 min and decreased to 5 % within 1 min before equilibrating with 5 % solvent B for 10 min. Elution is monitored by measuring the absorbance at 214 nm, and fractions are collected every 1 min. The peptides were subjected to nanoelectrospray ionization followed by tandem mass spectrometry (MS/MS) in an Q EXACTIVE (Thermo Fisher Scientific, San Jose, CA) coupled online to the HPLC. Intact peptides were detected in the Orbitrap at a resolution of 70,000. Peptides were selected for MS/MS using high-energy collision dissociation (HCD) operating mode with a normalized collision energy setting of 27.0; ion fragments were detected in the Orbitrap at a resolution of 17500. A data-dependent procedure that alternated between one MS scan followed by 15MS/MS scans was applied for the 15 most abundant precursor ions above a threshold ion count of 20,000 in the MS survey scan with a following dynamic exclusion duration of 15 s. The electrospray voltage applied was 1.6 kV. Automatic gain control (AGC) was used to optimize the spectra generated by the Orbitrap. The AGC target for full MS was 3e6 and 1e5 for MS2. For MS scans, them/z scan range was 350 to 2000Da. For MS2 scans, the m/z scan range was 100–1800.

### Proteomics data processing

The raw MS/MS data were converted into “.mgf” files using proteinpilot software (AB Sciex). Mascot version 2.3.0 (Matrix Sciences, London, UK) was used to search against the transcriptome database of *U. prolifera* and partion for other green algae proteins. The parameters were set as follows: peptide tolerance, 0.05 Da; fragment MS tolerance, 20 ppm; fixed modification, carbamidomethyl (C), iTRAQ8plex (N-term), iTRAQ8plex (K); and variable modifications including oxidation (M), iTRAQ8plex (Y). A maximum of one missed cleavage was allowed, and peptide charge states were set to +2 and +3. The search that was performed in Mascot was an automatic decoy database search. To identify false positives, raw spectra from the actual database were compared with a generated database of random sequences. Only peptides with significant scores at the 95 % confidence level were considered reliable and used for protein identification. For protein quantitation, a protein was required to contain at least two unique peptides. Protein quantitative ratios were weighted and normalized relative to the median ratio in Mascot. Only proteins with significant quantitative ratios between the two treatments (*p* < 0.05) and with fold changes >1.2 or <0.83 were considered differentially expressed.

To obtain a comprehensive understanding of the proteome and transcriptome profiles, correlation analysis was performed.

### PPI network analysis

The PPI data of *U. prolifera* were downloaded from the STRING database^54^. Each interaction has a combined score, which represents the reliability of the interaction between proteins. The PPI interactions with a combined score (0: lowest confidence; 1: highest confidence) larger than 0.4 were used for further network analysis. All differentially expressed proteins were mapped onto the PPI network and Cytoscape tool^55^ was used to visualise the network. GO term enrichment was determined by using the BiNGO plugin in Cytoscape.

### qRT-PCR validation of gene expression

The isolated RNA sequencing samples were also used to perform real-time quantitative (qRT-PCR) analysis. From the DEGs, 14 related genes were selected to verify the reliability of the transcriptome by real-time fluorescence quantitative PCR. The total RNA was reverse transcribed to a single-stranded cDNA using TransScript^®^ All-in-One First-Strand cDNA Synthesis SuperMix for qPCR (one-step gDNA removal) (Trans) according to the manufacturer’s instructions. Quantitative real-time PCR was performed on an ABI Quant Studio6 Flex (USA) using SYBR Green PCR kits (Trans, China) according to the manufacturers' instructions. β-actin as the control gene. PCR was performed according to the following procedure: 94 °C for 30 s, followed by 40 cycles of 94 °C for 5 s, 55 °C for 30 s, and 72 °C for 30 s. The relative expression was calculated using the 2^−ΔΔCT^ method, all reactions in triplicate times, each treatment has three biological repeats^56^.

### Related biological index and photosynthesis index determination

It was proposed that physiological adaptations of *U. prolifera* may enable it to survive the harsh intertidal environment and contribute to subsequent blooms. To investigate the effects of SA on antioxidant activities and photosynthesi indexs under high temperature, *U. prolifera* was treated at different SA concentration and times (35 °C, SA1 mmol·L^−1^ +35 °C, SA 0.1 mmol·L^−1^ +35 °C, SA 0.01 mmol·L^−1^ +35 °C, SA 0.001 mmol·L^−1^ +35 °C for 3, 6, 12, 24, 48 and 96 h). After treatment, *U. prolifera* were quickly frozen by liquid nitrogen and stored at −80 °C.

Pigment extraction and determination were weighed about 0.02 g of algae into the 10 mL centrifuge tube, add 5 mL of methanol. In the 4 °C refrigerator for 12 h. 2 mL supernatant was used to scan for 280–750 nm by a spectrophotometer. The contents of chlorophyll a (Chla) and chlorophyll b (Chlb) were calculated according to the formula^57^. Lipid peroxidation was determined by the estimation of malonaldehyde (MDA) with 2-thiobarbituric acid (TBA). *U. prolifera* were extracted in 10 mL of 0.25 % TBA in 10 % trichloroacetic acid. The mixture was incubated at 95 °C for 30 min and then cooled quickly on ice bath. The resulting content was centrifuged at 10,000 g for 15 min. The absorbance of the supernatant was recorded at 532 and 600 nm. The nonspecific absorbance at 600 nm was subtracted from the absorbance at 532 nm.

For antioxidative enzyme extraction 0.5 g *U. prolifera* was homogenized in 5.0 mL extraction buffer containing 1 mM EDTA, 0.05 % Triton-X-100, 2 % PVP, 1 mM ascorbate in 50 mM phosphate buffer, pH7.8. This mixture was centrifuged at 12,000 g for 20 min at 4 °C. Resulting supernatant was stored at −20 °C for the assay of following antioxidative enzymes. SOD activity was assayed by measuring its ability to inhibit the photochemical reduction of nitroblue tetrazolium by the method. The absorbance was taken at 560 nm and activity was expressed as lM NBT reduced min^−1^ mg^−1^ protein. Activity of APX is the rate of H_2_O_2_-dependent oxidation of ascorbic acid. Catalase activity was assayed as described by Vanacker *et al*.^58^.

### Determination of chlorophyll fluorescence parameters

Chlorophyll fluorescence parameters of algae were measured using a xenon lamp pulse modulation fluorescencemeter (Water-PAM). The maximum photochemical efficiency (Fv/Fm) and the effective photochemical efficiency (Fv′/Fm′) were obtained under culture condition. A fast light response curve (RLC) was measured at a gradient of 0, 34, 75, 114, 167, 257, 379, 570, 861 and 1218 µmol·m^−2^·s^−1^. rETR is the relative electron transport rate, according to the formula *rETR* = *rETR*_max_ (1−e^αx/*rETR*^_max_), *E*_k_ = *rETR*_max_/α, Ek is the initial light saturation point, and α is the apparent photosynthetic utilization, and Rk is the maximum relative electron transport rate. The liquid phase oxygen electrode (Hansatech, UK) was used to measure the rate of photosynthetic oxygen evolution of algae, and the water bath temperature was controlled at 25 °C using a three-hole thermostatic water tank. The oxygen electrode was maintained by a peristaltic pump. The water temperature is stable. Photosynthetic rates were determined at 0, 30, 60, 100, 150, 200, 250, and 500 μmol photons m^−2^ s^−1^. Net photosynthetic oxygenation rate and respiration rate were obtained.

## Electronic supplementary material

Supplementary information 

Supplementary File 1

Supplementary File 2
