# Supplementary material for: Integration of deep transcriptome and proteome analyses of salicylic acid regulation high temperature stress in Ulva prolifera
Source: Sci Rep. 2017 Sep 8;7:11052. doi: 10.1038/s41598-017-11449-w (PMC5591278; doi:10.1038/s41598-017-11449-w)
Supplement: Supplementary file 1 — Supplementary information [file 41598_2017_11449_MOESM1_ESM.doc]

**Integration of deep transcriptome and proteome analyses of salicylic acid regulation high temperature stress in *Ulva prolifera***

Meihua Fan^1,2 ＊^,Xue Sun^1^, Nianjun Xu^1＊^, Zhi Liao^2^, Yahe Li^1^, JianxinWang^2^, Yingping Fan^2^, Dalian Cui^2^, Peng Li^2^, Zengliang Miao^2^

**Supplementary Fig and table**


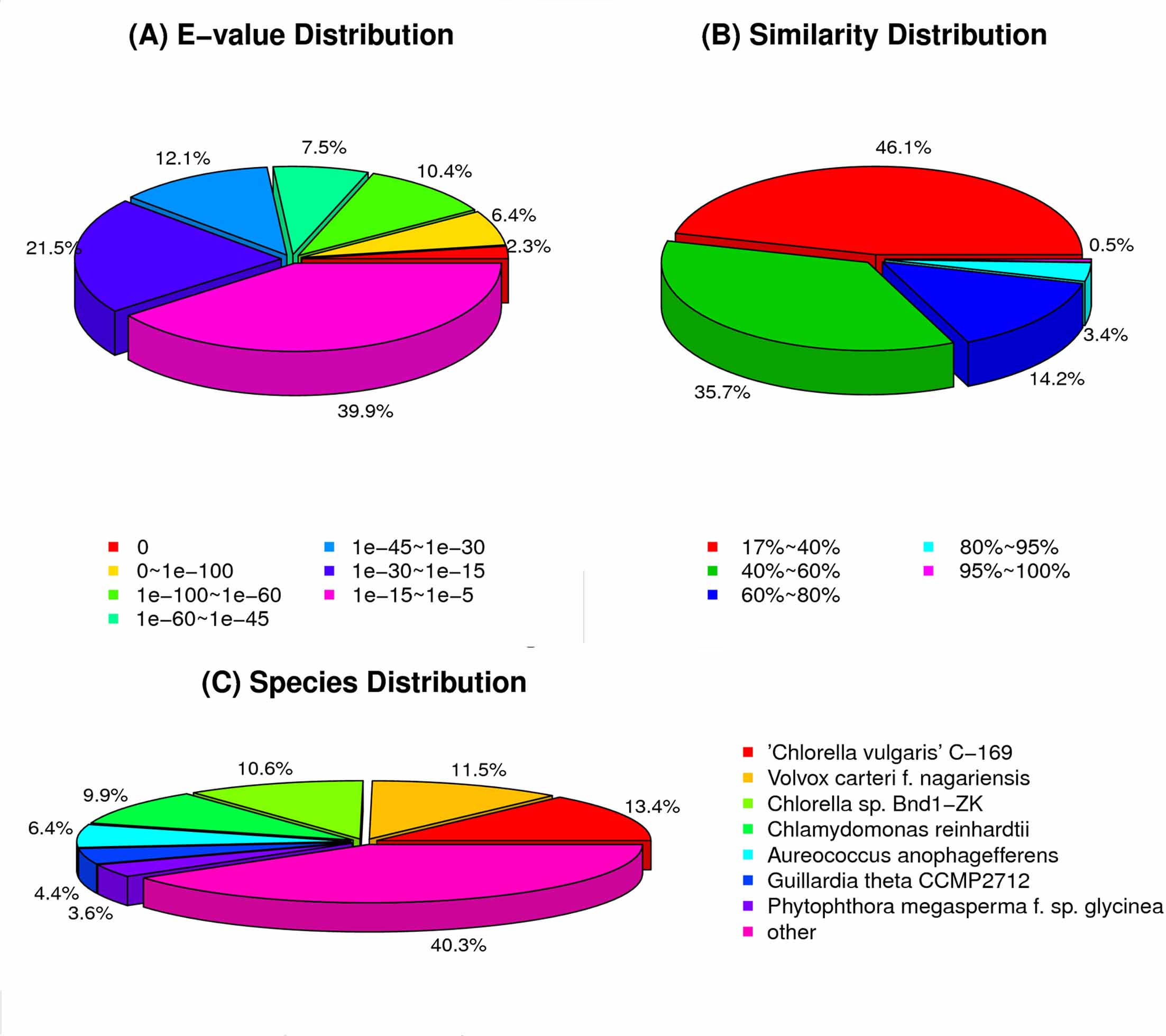


**Supplementary Fig.1 Classification of unigenes from *U. prolifera* based on NCBI-NR comparisons**.

(A) E-value distribution (B) similarity distribution and (C) species distribution.


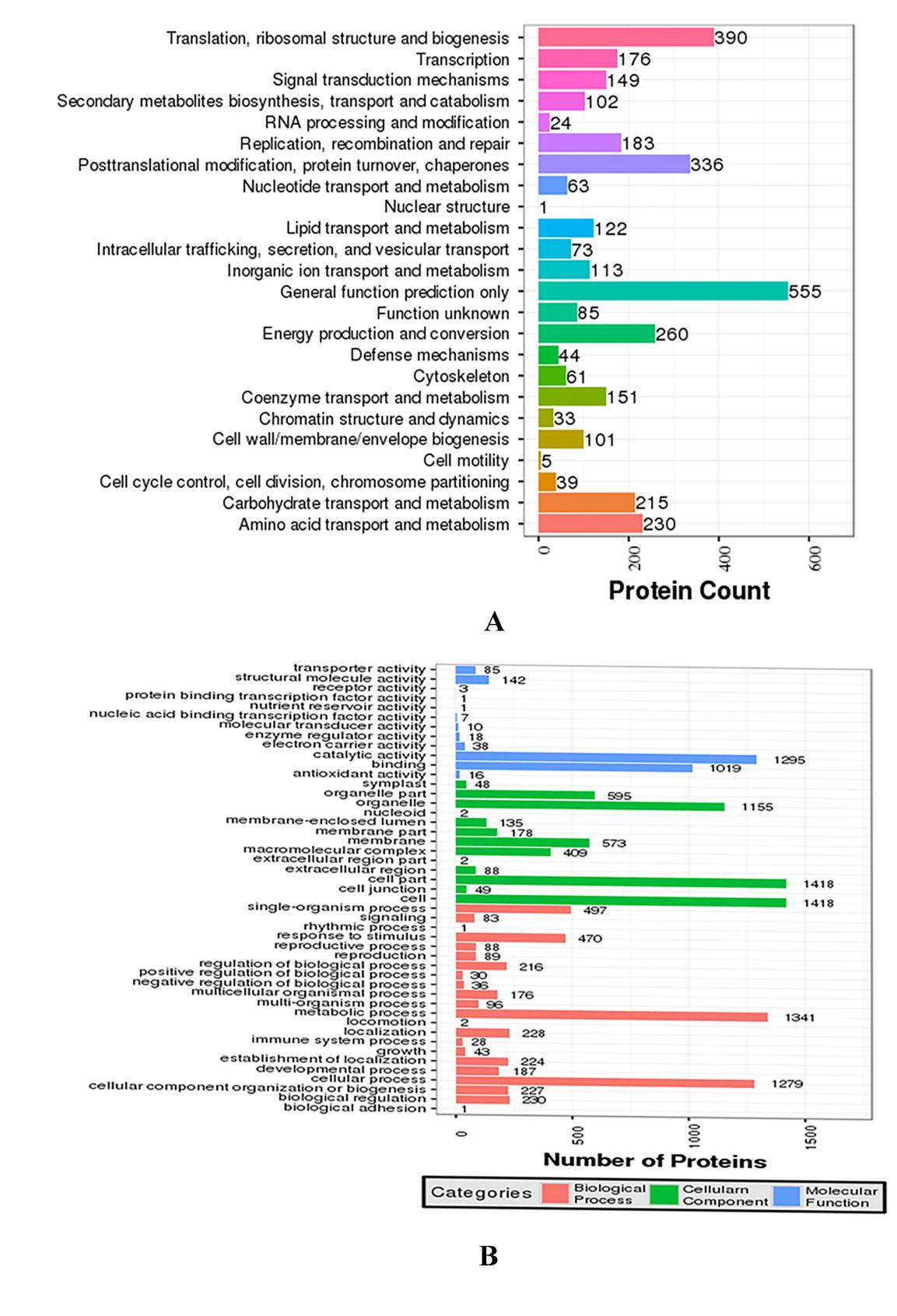


**Supplementary Fig. 2 Bar chart of COG (A) and GO(B) annotation for all-identified proteins**. Y-axis displays the COG term and GO terms, x-axis displays the corresponding protein count illustrating the protein number of different function.


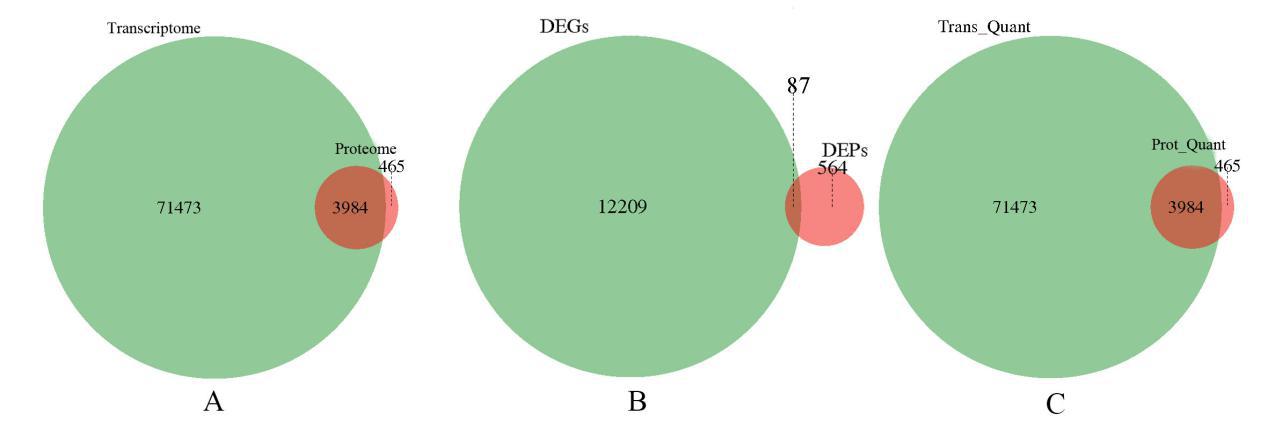


**Supplementary Fig. 3 Venny digram of transcriptome and proteome**


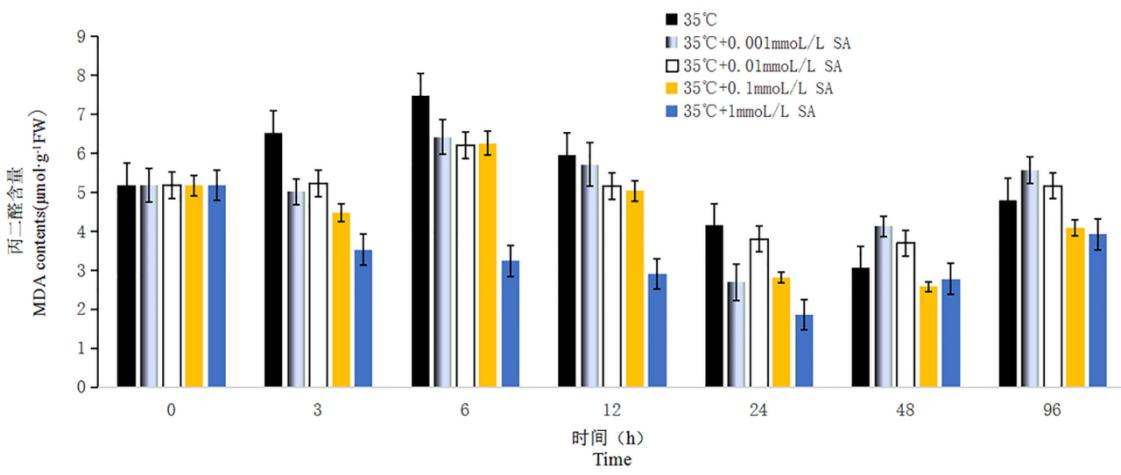


**Supplementary Fig. 4 Effects of salicylic acid on MDA content of *U. prolifera* under high temperature stress**


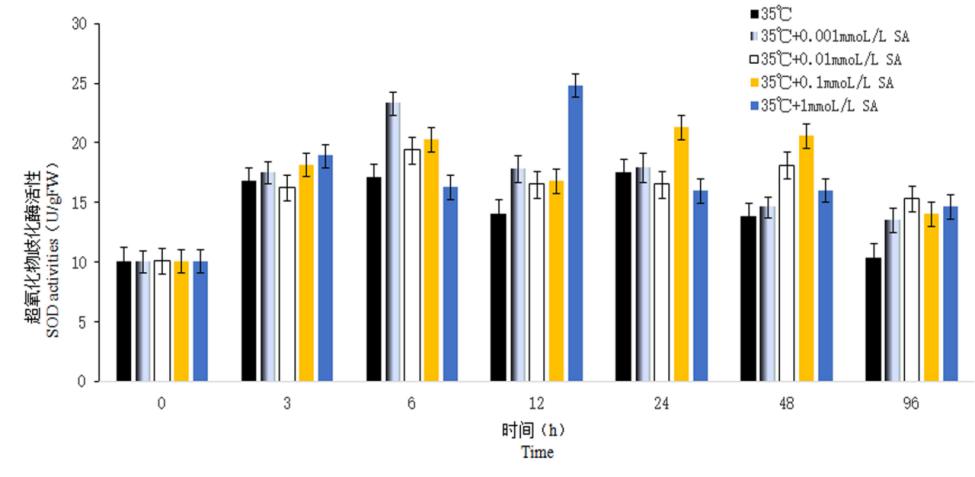


**Supplementary Fig. 5 Effects of salicylic acid on superoxide dismutase activity of *U. prolifera* under high temperature stress**


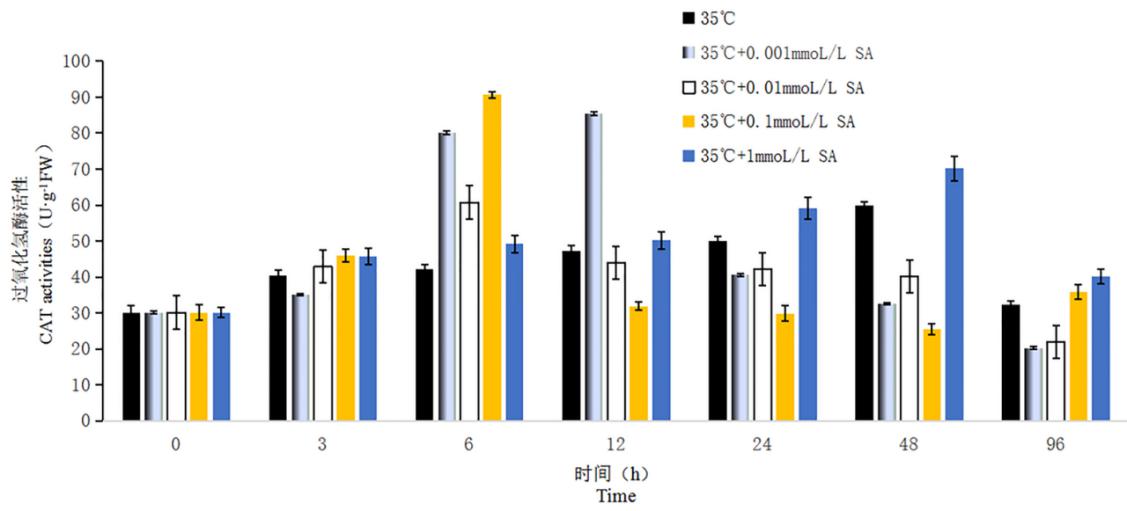


**Supplementary Fig. 6 Effects of salicylic acid on catalase activity of *U. prolifera* under high temperature stress**


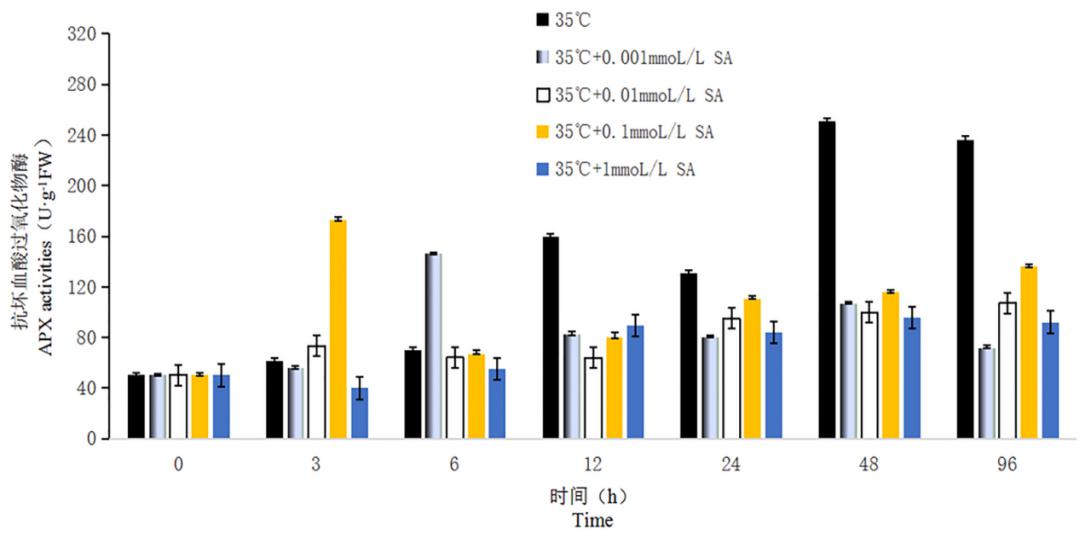


**Supplementary Fig. 7 Effects of salicylic acid on ascorbate peroxidase activity of *U. prolifera* under high temperature stress**

**
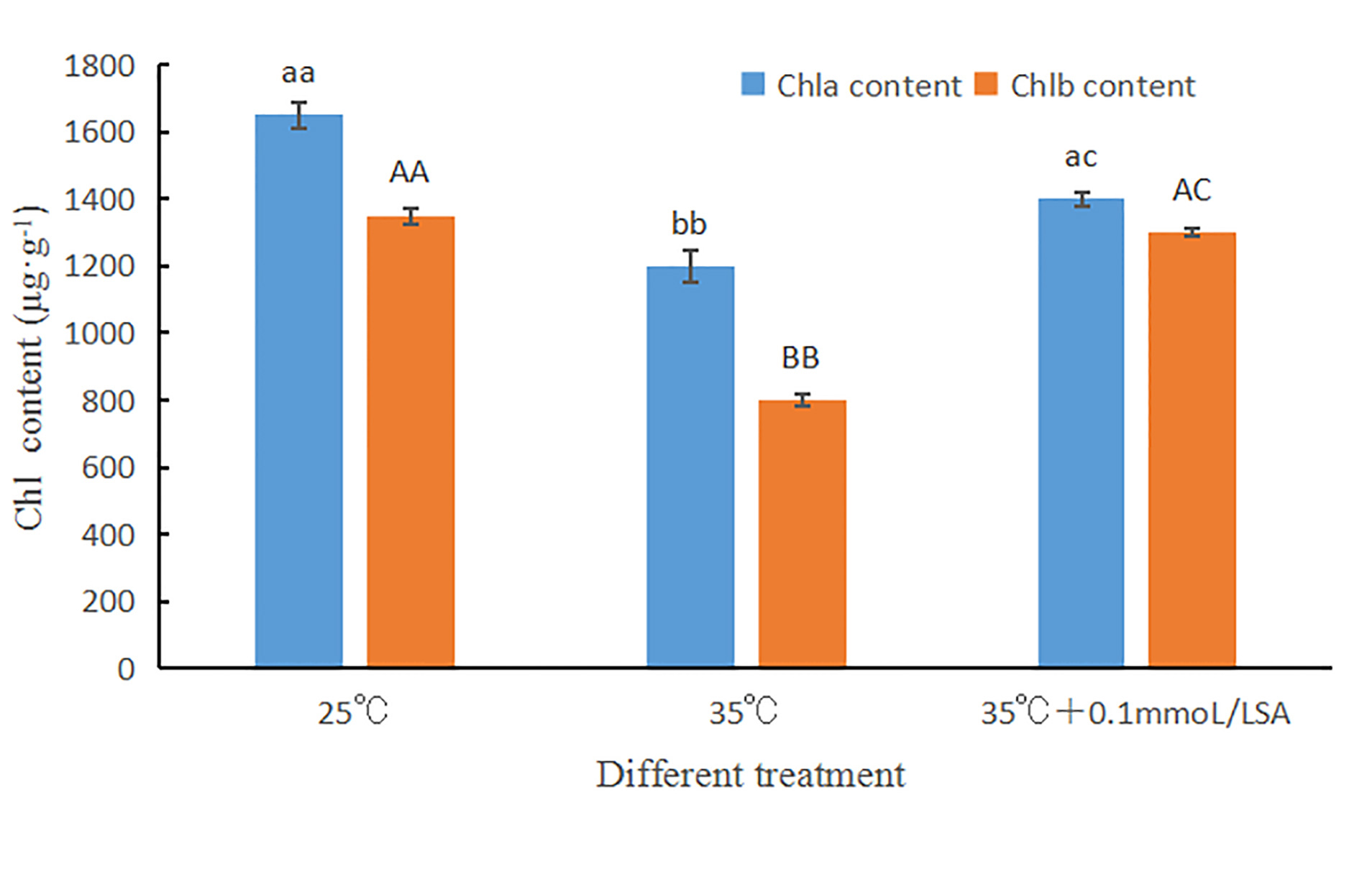
**

**Supplementary Fig. 8 Effects of salicylic acid on chlorophyll content of *U. prolifera* under high temperature stress**

**
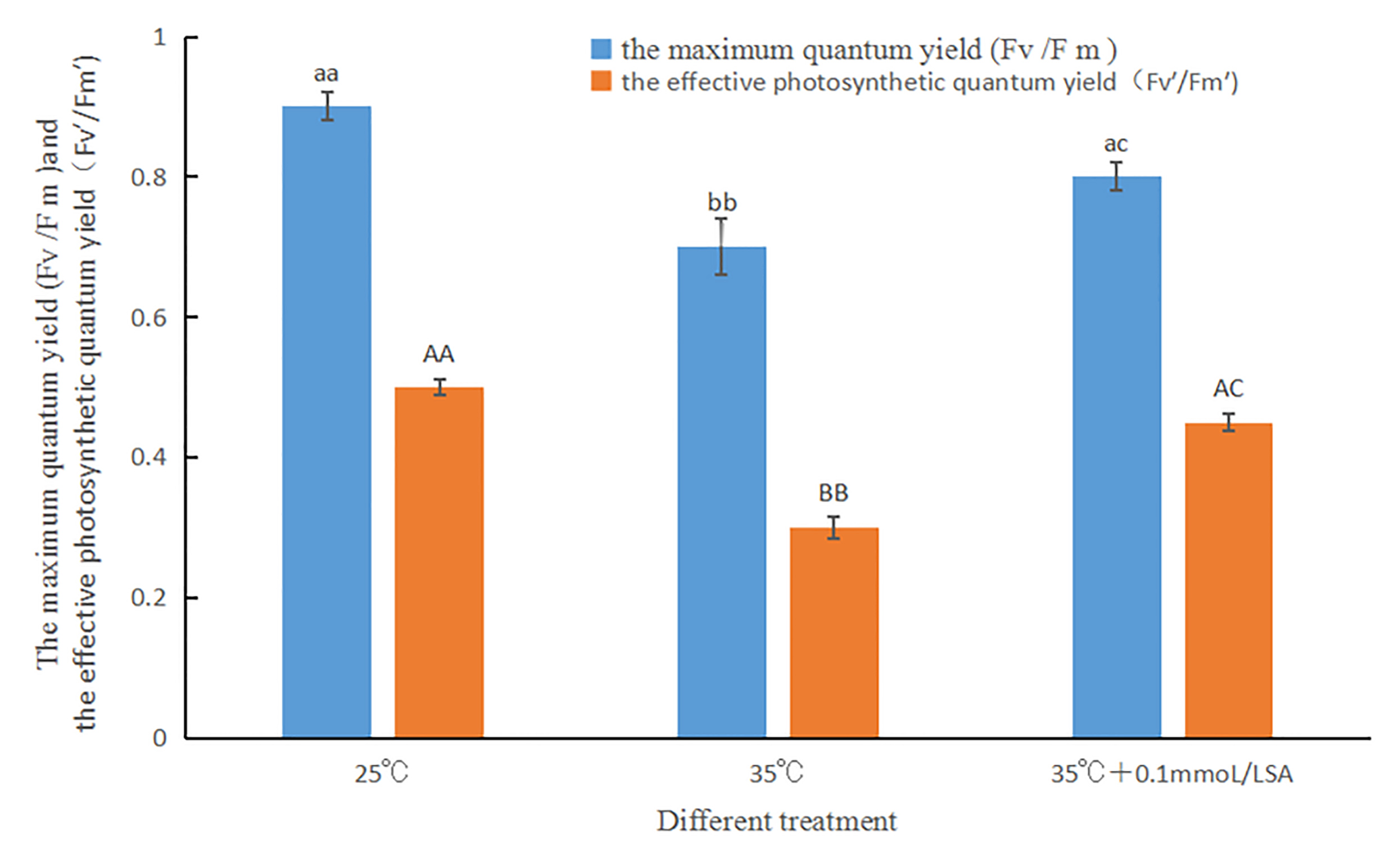
**

**Supplementary Fig. 9 Effects of salicylic acid on the maximum quantum yield (Fv/Fm ), effective photochemical quantum yield (Fv′/Fm′) of *U. prolifera* under high temperature stress**

**
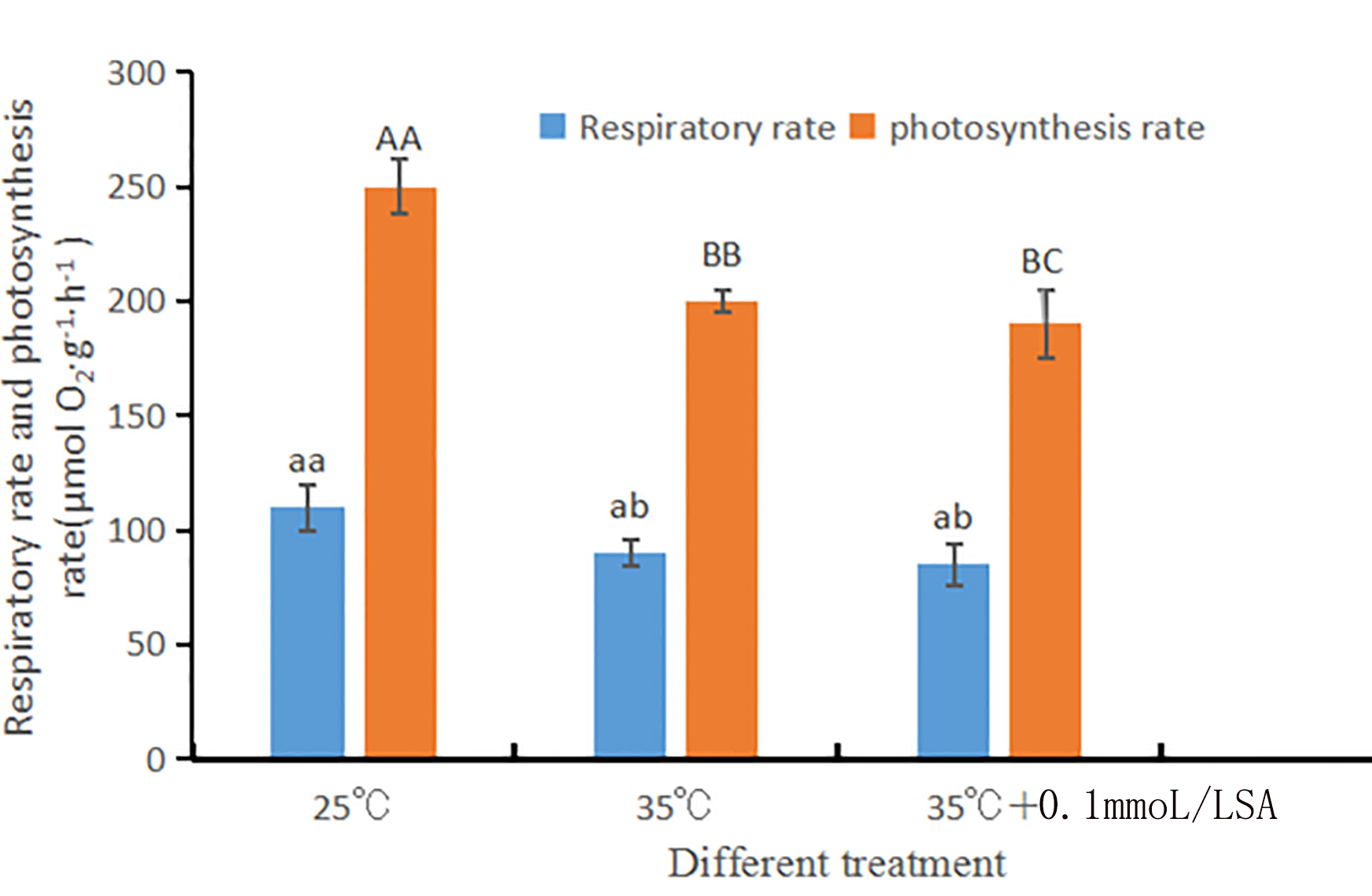
**

**Supplementary Fig. 10 Effects of salicylic acid on the respiratory rate and photosynthetic rate of *U. prolifera* under high temperature stress**

**Supplementary Table1**

**qPCR validation of the transcriptome data**

| Gene ID | Annotation | Primer | Primer sequence | | log2 ratio (UpSHT/UpHT) | qPCR (2^-ΔΔct^) |
| --- | --- | --- | --- | --- | --- | --- |
| Unigene44717_All | glutathione reductase | GR-F | | AGTGGTGGTGTAAGAGCAA | 10.9 | 4.273±0.09 |
|  |  | GR-R | | TGGACCCGTAAACAAATAG |  |  |
| CL6691.Contig1_All | MnSOD | SOD-F | | ATTCCAGATGGCTTTCAG | -12.1 | 0.383±0.012 |
|  |  | SOD-R | | CCAGGATACAGTGGCTCA |  |  |
| CL5666.Contig2_All | Heat shock protein 90 | HSP90-F | | GTTCTATGAGGCATTCGG | -15.776 | 0.152±0.006 |
|  |  | HSP90-R | | AGCTGTTGCACTGCGTAC |  |  |
| Unigene26892_All | Heat shock protein 70 | HSP70-F | | CAAGGCCACTGTGACCAA | -14.5 | 0.483±0.021 |
|  |  | HSP70-R | | ACCACCAGCACCACCATA |  |  |
| Unigene27760_All | JAZ | JAZ-F | | CAGGTGGTTCAGGCATAGT | -11.9 | 0.439±0.015 |
|  |  | JAZ-R | | GTAGAACCCACAAAGCCACCGACAC |  |  |
| Unigene24761_All | CAT | CAT-F | | CCAACGACTCCCGACACT | -12 | 0.216±0.010 |
|  |  | CAT-R | | AAGGTGGTAGTCCTCAAGCAG |  |  |
| Unigene25055_All | Peroxiredoxin5 | PRDX5-F | | CCAGTTTGCGCTTGCTAT | -11.3 | 13.523±0.872 |
|  |  | PRDX5-R | | CTGACCGTGTATGCTCCC |  |  |
| Unigene22247_All | NADPH-oxidase | NADPH-oxidase-F | | CGTCCAAAGTGCGTCAAT | -2.74 | 0.512±0.014 |
|  |  | NADPH-oxidase-R | | ATCAATATTTAACCTCCGTTTCCAA |  |  |
| Unigene28741_All | Prephenate dehydrogenase | PDH-F | | CGAACGACGATGCCTCAA | -11.21 | 0.206±0.009 |
|  |  | PDH-R | | GCACCACCCACAGCTCAA |  |  |
| Unigene21876_All | PEX19 | PEX19-F | | TGGGCAAGATGAGATACAAC | -10.6 | 0.054±0.001 |
|  |  | PEX19-R | | TCCTGAAGTGGGTCAAACA |  |  |
| Unigene28379_All | PEX10 | PEX10-F | | CGGAACGTCGTCTTTGGG | -11.24 | 0.361±0.011 |
|  |  | PEX10-R | | CCGCAGCACAGCTTGAT |  |  |
| CL4094.Contig1_All | Cyclic nucleotide-gated  channels (Ca^2+^) | CNGCS-F | | CAACCCGAGCCTCTGTAA | -11.7 | 0.360±0.021 |
|  |  | CNGCS-R | | GGTGATCTGAGCACGGATAT |  |  |
| Unigene25439_All | Trans-cinnamate 4  -monooxygenase | TC4M-F | | ACTGGCATTCTGCTACTCTT | -10.8 | 0.184±0.015 |
|  |  | TC4M-R | | CATCTCAATCCGACTCACC |  |  |
| Unigene20951_All | Cytokinin trans-hydroxylase | CKTH-F | | GACAGTGCTTTCCCATCT | -10.8 | 0.206±0.018 |
|  |  | CKTH-R | | GCTACTTCATTACCCTCGT |  |  |
|  |  | β-Actin-F | | AGGATGCATACGTTGGTGAA |  |  |
|  |  | β-Actin-R | | TTGTGGTGCCAAATCTTCTC |  |  |

Supplementary Table2 The fitted parameters of relative electron transport rates (rETR) and light intensity indices of Ulva prolifera under different treatments（Ek： The initial light saturation point；rETRmax：The maximal rate of rETR；α：The apparent photosynthetic efficiency）

|  | 25℃ | 35℃ | 35℃+0.1mmoL/LSA |
| --- | --- | --- | --- |
| *E*_k_ | 268.25±20.946 | 238.70±22.10 | 322.35±23.87 |
| α | 0.21±0.01 | 0.20±0.03 | 0.19±0.05 |
| *rETR*_max_ | 56.09±3.74 | 44.43±5.83 | 62.88±11.70 |
