# Supplementary material for: Integration of deep transcriptome and proteome analyses of salicylic acid regulation high temperature stress in Ulva prolifera
Source: Sci Rep. 2017 Sep 8;7:11052. doi: 10.1038/s41598-017-11449-w (PMC5591278; doi:10.1038/s41598-017-11449-w)
Supplement: Supplementary file 2 — Supplementary File 1 [file 41598_2017_11449_MOESM2_ESM.pdf]

UpSHT-VS-UpHT Down (DEPs portion)

No.	Protein_ID	Description	Mean_Ratio_UpSHT-VS-UpHT	SD_UpSHT-VS-UpHT	P value
154	CL595_Contig2_All	oxidoreductase [*Volvox carteri* f. *nagariensis*]	1.25	0.033	0.003397
188	CL5484_Contig2_All	ribosomal protein S6 [*Chlamydomonas reinhardtii*]	1.21	0.007	7.25E-05
259	Unigene29891_All	243 452 predicted protein [*Bathycoccus prasinos*]	1.48	0.138	0.004794
268	CL7120_Contig2_All	281 1024 soluble carbonic anhydrase precursor [*Chlorella sorokiniana*]	1.35	0.055	0.00192
311	gi|145568329|gb|ABP82521_1|	ribulose-1,5-bisphosphate carboxylase/oxygenase large subunit, partial (chloroplast) [*Ulva* sp.WELT A027747]	1.32	0.021	0.0001664
333	gi|158271131|gb|EDO96957_1|	peptidyl-prolyl cis-trans isomerase, cyclophilin-type [*Chlamydomonas reinhardtii*]	1.35	0.166	0.0319
397	CL206_Contig2_All	1 477 PREDICTED: elongation factor 2-like [*Solanum lycopersicum*]	1.81	0.217	0.0007856
416	Unigene14870_All	362 1093 cysteine proteinase [*Coccomyxa subellipsoidea* C-169]	1.26	0.015	0.0002143
490	gi|807046156|gb|AKC35214_1|	photosystem I reaction center subunit IX (chloroplast) [*Ulva fasciata*]	1.47	0.146	0.006013
571	gi|158277228|gb|EDP02997_1|	predicted protein, partial [*Chlamydomonas reinhardtii]*	1.21	0.029	0.006593
732	Unigene47331_All	1 249 LEN=249; minus strand; translated	1.36	0.111	0.01242
872	CL574_Contig1_All	71 214 minus strand ribosomal protein L14b/L23e [*Coccomyxa subellipsoidea* C-169]	1.93	0.135	6.48E-05
953	Unigene3975_All	predicted protein [*Micromonas* sp.]	1.22	0.066	0.03154
960	gi|960515081|gb|ALR86928_1|	photosystem II D2 protein (chloroplast) [*Ulva fasciata*]	1.25	0.061	0.01478
1073	gi|145567817|gb|ABP82265_1|	ribulose-1,5-bisphosphate carboxylase/oxygenase large subunit, partial (chloroplast) [*Ulva* sp.]	2.14	0.439	0.0008819
1156	Unigene8534_All	vacuolar H(+)-ATPase V0 sector, c/c' subunits [*Chlamydomonas reinhardtii*]	1.26	0.03	0.001851
1168	CL1298_Contig4_All	scavenger receptor cysteine-rich protein [*Chlamydomonas reinhardtii*]	1.44	0.295	0.04136
1183	CL1586_Contig4_All	149 1294 METM1f [*Volvox carteri* f. *nagariensis*]	1.25	0.01	6.93E-05
1230	gi|125577|sp|P19824_1|KPPR_CHLRE	phosphoribulokinase precursor [*Chlamydomonas reinhardtii*]	1.46	0.272	0.03031
1271	Unigene30462_All	1 354 LEN=354; translated	1.76	0.409	0.007515
1305	gi|300263255|gb|EFJ47457_1|	microtubule-associated protein [*Volvox carteri* f. *nagariensis*]	1.5	0.374	0.03988
1473	Unigene43911_All	1 283 LEN=283; minus strand; translated	1.24	0.037	0.005338
1560	CL1348_Contig3_All	1164 1871 LEN=2190; translated	1.66	0.063	5.04E-05
1588	CL1835_Contig3_All	predicted protein [*Micromonas pusilla* CCMP1545]	1.21	0.075	0.04867
1595	Unigene20506_All	predicted protein [*Chlamydomonas reinhardtii*]	1.3	0.102	0.02119
1767	gi|145567588|gb|ABP82151_1|	ribulose-1,5-bisphosphate carboxylase/oxygenase large subunit, partial (chloroplast) [*Ulva* sp.]	1.33	0.067	0.005011
1786	CL6502_Contig1_All	3 452 actin-related protein 2 [*Ectocarpus siliculosus*]	1.3	0.042	0.002195
1857	CL1385_Contig1_All	25 1437 minus strand translation elongation factor-like protein [*Karlodinium veneficum*]	1.31	0.025	0.0004046
1890	Unigene7937_All	predicted protein [*Ostreococcus lucimarinus* CCE9901]	1.23	0.028	0.003286
1981	Unigene3941_All	212 928 minus strand PREDICTED: uncharacterized protein LOC100809428 [*Glycine max*]	1.36	0.035	0.0004349
2072	CL4638_Contig1_All	speract/scavenger receptor, transmembrane glycoprotein [*Chlamydomonas reinhardtii*]	1.27	0.041	0.004165

2111	CL2355_Contig1_All	rieske [2Fe-2S] protein [*Chlamydomonas reinhardtii*]	1.23	0.013	0.0003328
2227	CL73_Contig7_All	neurotransmitter:Sodium symporter family [*Micromonas pusilla* CCMP1545]	1.23	0.065	0.02838
2369	Unigene4453_All	predicted protein [*Chlamydomonas reinhardtii*]	1.31	0.151	0.04445
2433	Unigene5712_All	ribosomal protein S23 [*Chlamydomonas reinhardtii*]	1.29	0.015	0.0001246
2538	CL7534_Contig2_All	71 907 minus strand TPA_inf: chloroplast light-harvesting protein CP29 precursor Lhcb4 [*Acetabularia acetabulum*]	2.51	3.765	0.04742
2573	CL4954_Contig1_All	GTP binding protein [*Chlamydomonas reinhardtii*]	1.33	0.184	0.0493
2641	Unigene42677_All	aspartate aminotransferase [*Volvox carteri* f. *nagariensis*]	1.21	0.026	0.004925
2672	CL1069_Contig4_All	111 2189 type II DNA topoisomerase [*Coccomyxa subellipsoidea* C-169]	1.92	0.268	0.0006893
2674	gi|145567931|gb|ABP82322_1|	ribulose-1,5-bisphosphate carboxylase/oxygenase large subunit, partial (chloroplast) [*Ulva* sp.]	1.32	0.044	0.00169
2712	Unigene45681_All	1 237 LEN=237; translated	1.3	0.1	0.02061
2781	gi|1172455|sp|P41758_1|PGKH_CHLRE	phosphoglycerate kinase precursor [*Chlamydomonas reinhardtii*]	2.26	0.725	0.002125
2830	Unigene16177_All	predicted protein [*Micromonas* sp.]	1.53	0.162	0.004367
2831	gi|1001185378|gb|AML80578_1|	cytochrome c oxidase subunit 1 (mitochondrion) [*Ulva linza*]	1.26	0.072	0.01879
2891	CL4698_Contig2_All	4 393 minus strand oxoglutarate:malate antiporter, partial [*Coccomyxa subellipsoidea* C-169]	1.25	0.085	0.03149
2925	Unigene13612_All	96 1424 tubulin folding cofactor E [*Arabidopsis thaliana*]	1.26	0.009	3.73E-05
2949	Unigene35497_All	1 200 LEN=200; minus strand; translated	1.55	0.432	0.03688
2986	gi|291586512|gb|ADE19008_1|	elongation factor Tu, partial (chloroplast) [*Ulva sp*.]	1.78	0.523	0.01186
3005	gi|1703374|sp|P51821_2|ARF1_CHLRE	ADP-ribosylation factor [*Chlamydomonas reinhardtii*]	1.99	0.92	0.015
3019	gi|158273526|gb|EDO99315_1|	cytochrome P450, CYP85 clan, partial [*Chlamydomonas reinhardtii*]	1.46	0.043	0.0001501
3082	Unigene36107_All	1 209 LEN=209; translated	1.28	0.119	0.03959
3092	Unigene31707_All	1 265 LEN=265; translated	2.13	0.585	0.002185
3151	Unigene4462_All	predicted protein [*Populus trichocarpa*]	1.23	0.062	0.0256
3188	Unigene14237_All	5205 5540 minus strand unknown [*Picea sitchensis*]	1.3	0.046	0.00286
3257	CL1808_Contig1_All	SR protein factor [*Chlamydomonas reinhardtii*]	1.22	0.071	0.03874
3436	Unigene14533_All	1 1915 LEN=1915; translated	1.23	0.045	0.01081
3497	CL4080_Contig2_All	subunit of Retromer complex [*Volvox carteri* f.*nagariensis*]	1.21	0.073	0.04738
3498	Unigene37824_All	53 235 minus strand tubulin beta [*Nannochloropsis gaditana* CCMP526]	1.41	0.243	0.03701
3550	CL705_Contig3_All	1406 2563 minus strand Six-hairpin glycosidase [*Coccomyxa subellipsoidea* C-169]	1.29	0.064	0.009133
3611	Unigene194_All	2 1048 minus strand predicted protein [*Hordeum vulgare*]	1.28	0.02	0.0003309
3627	Unigene11752_All	speract/scavenger receptor, transmembrane glycoprotein [*Chlamydomonas reinhardtii*]	1.46	0.144	0.006338
3649	CL4473_Contig1_All	307 1569 minus strand Clc chloride channel [*Coccomyxa subellipsoidea* C-169]	1.25	0.087	0.03196
3727	CL7048_Contig1_All	predicted protein [*Micromonas pusilla* CCMP1545]	1.34	0.103	0.01328
3749	CL930_Contig2_All	glutamate-1-semialdehyde aminotransferase [*Chlamydomonas reinhardtii*]	1.65	0.192	0.002062

3882	CL7030_Contig6_All	predicted protein [*Hordeum vulgare* subsp_ vulgare]	1.34	0.03	0.0003803
3979	Unigene25417_All	1 241 LEN=241; translated	1.21	0.037	0.01218
3999	Unigene32478_All	1 406 LEN=406; translated	1.26	0.028	0.001478
4065	Unigene1828_All	244 1173 PREDICTED: importin-9-like [*Glycine max*]	1.23	0.011	0.0001478
4081	CL3790_Contig1_All	type II secretory pathway family [*Micromonas* sp. RCC299]	1.37	0.048	0.0009598
4099	Unigene13669_All	flagellar-specific protein Ssa14 [*Volvox carteri* f. nagariensis]	1.24	0.03	0.002782
4139	Unigene42972_All	1 734 LEN=734; translated	1.42	0.043	0.0002973
4183	gi|158274793|gb|EDP00573_1|	unnamed protein product [*Chlamydomonas reinhardtii*]	1.51	0.174	0.006423
4201	Unigene5510_All	195 1070 unknown [*Picea sitchensis*]	1.28	0.03	0.001413
4254	CL4711_Contig1_All	2-oxoglutarate/malate translocator [*Chlamydomonas reinhardtii*]	1.3	0.152	0.04816
4272	Unigene40567_All	1 279 LEN=279; translated	1.41	0.173	0.01825
4301	Unigene2464_All	78 1415 minus strand eukaryotic translation elongation factor 1 alpha [*Boodlea coacta*]	1.51	0.343	0.03038
4324	gi|452119419|gb|AGG09538_1|	elongation factor Tu, partial (chloroplast) [*Ulva prolifera*]	1.48	0.159	0.006994
4401	Unigene20821_All	1 222 Six-hairpin glycosidase [*Coccomyxa subellipsoidea* C-169]	1.26	0.087	0.02976

UpSHT-VS-UpHT Down (DEPs portion)

No.	Protein_ID	Description	Mean_Ratio_Up!	SD_UpSHT-VS-UpI	Pvalue
14	Unigene38388_All	1 330 LEN=330; translated	0.79	0.02	0.001989
20	CL6101_Contig2_All	thioredoxin m [*Chlamydomonas reinhardtii*]	0.7	0.008	7.43E-06
24	CL7140_Contig2_All	1 597 LEN=597; translated	0.78	0.012	0.0003069
38	gi|300269063|gb|EFJ53243_	acetylglucosaminyltransferase [*Volvox carteri* f. *nagariensis*]	0.61	0.015	1.40E-05
63	Unigene7624_All	NDPK2a nucleotide diphosphate kinase 2 protein [*Physcomitrella patens* subsp_ patens]	0.8	0.006	4.62E-05
75	CL152_Contig5_All	109 495 minus strand PREDICTED: LOW QUALITY PROTEIN: ubiquitin-60S ribosomal protein L40 [*Vitis vinifera*]	0.83	0.013	0.002318
88	Unigene43358_All	1 472 LEN=472; minus strand; translated	0.78	0.043	0.01282
114	CL2455_Contig1_All	171 899 minus strand electron transfer flavo protein beta-subunit [*Coccomyxa subellipsoidea* C-169]	0.75	0.009	5.51E-05
149	CL1577_Contig1_All	176 649 minus strand hexokinase 1a [*Nicotiana tabacum*]	0.78	0.062	0.03081
162	CL5969_Contig3_All	Os07g0642300 [*Oryza sativa* Japonica Group]	0.73	0	8.27E-11
163	CL881_Contig2_All	peptidyl-prolyl cis-trans isomerase, cyclophilin-type [*Chlamydomonas reinhardtii*]	0.76	0.002	1.19E-07
168	Unigene43365_All	10 KDa phosphoprotein of photosystem II [*Pseudendoclonium akinetum*]	0.74	0.029	0.001919
170	Unigene2995_All	1 267 LEN=267; translated	0.78	0.009	0.0001055
175	CL2825_Contig3_All	component of cytosolic 80S ribosome and 40S small subunit [*Volvox carteri* f. *nagariensis*]	0.76	0.004	2.86E-06
194	CL2925_Contig2_All	52 702 Histone H1-II OS=Volvox carteri GN=H1-II PE=2 SV=3	0.83	0.008	0.0003523
215	Unigene34718_All	1 207 LEN=207; minus strand; translated	0.82	0.011	0.001014
237	Unigene3852_All	156 368 plastid acyl carrier protein [*Helicosporidium* sp. ex Simulium jonesi]	0.64	0.028	0.0002152
238	Unigene15924_All	predicted protein [*Chlamydomonas reinhardtii*]	0.65	0.055	0.002006
284	CL1817_Contig4_All	1455 2984 predicted protein [*Populus trichocarpa*]	0.83	0.001	8.43E-07
289	CL2580_Contig1_All	574 1506 minus strand plastid terminal oxidase [*Cyanidioschyzon merolae* strain 10D]	0.77	0.042	0.009641
329	CL2614_Contig3_All	108 689 Arf6/ArfB-family small GTPase [*Coccomyxa subellipsoidea* C-169]	0.8	0.007	0.0001191
330	CL2080_Contig1_All	71 1297 26S proteasome non-ATPase regulatory subunit 11 [*Zea mays*]	0.78	0.022	0.002094
343	Unigene12487_All	1 472 LEN=472; minus strand; translated	0.78	0.016	0.0009319
346	CL2527_Contig2_All	4-alpha-glucanotransferase [*Chlamydomonas reinhardtii*]	0.83	0.002	2.35E-06
367	CL3386_Contig1_All	300 764 minus strand thioredoxin-like protein [*Coccomyxa subellipsoidea* C-169]	0.68	0.034	0.0007802
372	Unigene5430_All	381 2138 predicted protein [*Hordeum vulgare* subsp_ vulgare]	0.78	0.008	6.84E-05
376	Unigene20819_All	86 700 minus strand F1F0 ATP synthase gamma subunit [*Coccomyxa subellipsoidea* C-169]	0.78	0.004	5.80E-06
382	CL2860_Contig1_All	132 1418 minus strand PLC-like phosphodiesterase [*Coccomyxa subellipsoidea* C-169]	0.83	0.012	0.001501
384	gi|158283995|gb|EDP09745	SR protein factor [*Chlamydomonas reinhardtii*]	0.78	0.027	0.003826
386	CL4608_Contig2_All	ribosomal protein [*Micromonas pusilla* CCMP1545]	0.83	0.007	0.0002488
388	CL4038_Contig3_All	2 259 PREDICTED: cytochrome c1-1, heme protein, mitochondrial-like [*Glycine max*]	0.75	0.008	4.12E-05

412	CL4858_Contig3_All	532 1020 EF-hand [*Coccomyxa subellipsoidea* C-169]	0.7	0.028	0.0007527
427	Unigene11937_All	269 609 LEN=609; translated	0.71	0.01	2.19E-05
462	gi|807046094|gb|AKC35152	ATP synthase subunit b (chloroplast) [*Ulva* sp.]	0.63	0.025	0.0001094
492	CL1786_Contig3_All	26S proteasome regulatory subunit [*Chlamydomonas reinhardtii*]	0.75	0.007	1.52E-05
519	Unigene1435_All	predicted protein [*Chlamydomonas reinhardtii*]	0.79	0.001	2.49E-08
522	gi|807046122|gb|AKC35180	50S ribosomal protein L5 (chloroplast) [*Ulva* sp.]	0.7	0.011	2.33E-05
551	CL6394_Contig1_All	578 1156 beta-lactamase-like protein [*Rhodiola fastigiata*]	0.73	0.013	0.0001157
552	CL966_Contig1_All	subunit of the ESCRT-I complex [*Chlamydomonas reinhardtii*]	0.7	0	1.54E-12
574	Unigene13592_All	675 1244 LEN=1555; minus strand; translated	0.48	0.008	1.11E-07
588	CL1190_Contig1_All	13 327 minus strand unknown [*Picea sitchensis*]	0.75	0.1	0.04755
591	CL3797_Contig1_All	255 1139 minus strand elongation factor Ts [*Coccomyxa subellipsoidea* C-169]	0.8	0.01	0.0002409
601	CL7352_Contig1_All	371 682 minus strand Frataxin [*Coccomyxa subellipsoidea* C-169]	0.72	0.015	0.0001184
620	Unigene18298_All	13 1023 FAD/NAD(P)-binding domain-containing protein [*Coccomyxa subellipsoidea* C-169]	0.81	0.008	0.0002074
622	gi|315319013|gb|ADU04518	LhcSR [*Ulva prolifera*]	0.76	0.021	0.001007
623	CL3367_Contig2_All	250 1284 anion-transporting ATPase [*Coccomyxa subellipsoidea* C-169]	0.76	0.005	4.92E-06
643	CL1383_Contig2_All	300 806 minus strand NADH-quinone oxidoreductase, partial [*Coccomyxa subellipsoidea* C-169]	0.74	0.029	0.001639
654	CL1390_Contig2_All	7 735 predicted protein [*Hordeum vulgare* subsp_ vulgare]	0.61	0.041	0.0004018
690	CL7578_Contig1_All	predicted protein [*Chlamydomonas reinhardtii*]	0.7	0.006	3.12E-06
695	Unigene6725_All	53 280 minus strand polyubiquitin [*Aureococcus anophagefferens*]	0.83	0.027	0.01657
703	CL597_Contig1_All	75 407 minus strand 60s acidic ribosomal protein-like protein [*Solanum tuberosum*]	0.63	0.008	1.42E-06
730	CL964_Contig2_All	Qa-SNARE Sso1/Syntaxin1, PM-type [*Chlamydomonas reinhardtii*]	0.77	0.057	0.01965
736	Unigene12267_All	171 1661 putative carboxypeptidase [*Coccomyxa subellipsoidea* C-169]	0.74	0.005	3.86E-06
740	gi|960515061|gb|ALR86908	30S ribosomal protein S8 (chloroplast) [*Ulva fasciata*]	0.55	0.008	4.16E-07
772	gi|158271390|gb|EDO97210	flagellar associated protein, cobalamin adenosyltransferase-like protein [*Chlamydomonas reinhardtii*]	0.65	0.018	5.06E-05
786	Unigene5919_All	128 784 glutathione S-transferase [*Coccomyxa* sp.]	0.73	0.024	0.0007462
796	CL3741_Contig2_All	farnesyl diphosphate synthase [*Chlamydomonas reinhardtii*]	0.81	0.028	0.0104
807	CL2484_Contig2_All	172 1617 putative 26S proteasome regulatory subunit [*Coccomyxa subellipsoidea* C-169]	0.82	0.013	0.001557
810	CL646_Contig1_All	2731 3276 minus strand chloroplast oxygen-evolving protein 3 [*Chlamydomonas incerta*]	0.51	0.017	3.58E-06
826	Unigene11822_All	predicted protein [*Micromonas* sp. RCC299]	0.75	0.02	0.0007952
828	CL2416_Contig1_All	plastid ribosomal protein L10 [*Chlamydomonas reinhardtii*]	0.64	0.001	1.44E-10
839	CL6565_Contig2_All	photosystem I reaction center subunit II, 20 kDa [*Chlamydomonas reinhardtii*]	0.82	0.009	0.0005726
847	CL2524_Contig2_All	459 1145 PREDICTED: putative uridine kinase C227_14-like [*Cucumis sativus*]	0.81	0.004	2.13E-05
848	Unigene1814_All	3 152 17_8 kDa class I heat shock protein OS=Arabidopsis thaliana GN=HSP17_8 PE=2 SV=1	0.73	0.016	0.0002223

849	CL2631_Contig2_All	566 1192 LEN=1873; translated	0.69	0.052	0.00342
851	CL3807_Contig1_All	predicted protein [*Chlamydomonas reinhardtii*]	0.7	0.007	4.08E-06
853	Unigene28145_All	predicted protein [*Chlamydomonas reinhardtii*]	0.78	0.01	0.0002001
876	CL4742_Contig1_All	predicted protein [*Micromonas pusilla* CCMP1545]	0.8	0.013	0.0009932
883	Unigene11934_All	mitochondrial tRNA import complex [*Micromonas* sp. RCC299]	0.52	0.026	1.91E-05
885	CL3586_Contig2_All	predicted protein [*Micromonas* sp.RCC299]	0.81	0.006	8.98E-05
906	CL3783_Contig2_All	378 569 LEN=569; translated	0.76	0.02	0.0007981
912	CL408_Contig1_All	200 763 soluble inorganic pyrophosphatase 2 [*Coccomyxa subellipsoidea* C-169]	0.68	0.007	3.14E-06
914	CL5198_Contig1_All	26S proteasome regulatory complex [*Volvox carteri* f. *nagariensis*]	0.72	0.004	1.21E-06
934	Unigene5985_All	predicted protein [*Micromonas pusilla* CCMP1545]	0.78	0.003	1.27E-06
938	CL2953_Contig1_All	N-carbamyl-L-amino acid amidohydrolase [*Chlamydomonas reinhardtii*]	0.78	0.005	1.58E-05
952	Unigene575_All	component of cytosolic 80S ribosome and 60S large subunit [*Volvox carteri* f. *nagariensis*]	0.7	0.072	0.009206
978	Unigene163_All	438 1154 minus strand glutathione S-transferase [*Coccomyxa subellipsoidea* C-169]	0.64	0.006	5.11E-07
1012	Unigene36640_All	1 204 LEN=204; translated	0.67	0.044	0.001433
1031	Unigene7736_All	220 438 minus strand NaCl-inducible protein [*Coccomyxa subellipsoidea* C-169]	0.58	0.034	0.0001183
1040	Unigene1392_All	predicted protein [*Populus trichocarpa*]	0.79	0.01	0.0001839
1061	CL3256_Contig5_All	component of TRAPP complex [*Chlamydomonas reinhardtii*]	0.71	0.008	9.98E-06
1064	Unigene39765_All	predicted protein [*Thalassiosira pseudonana* CCMP1335]	0.67	0.022	0.000151
1070	gi|300266134|gb|EFJ50322_	plastid/chloroplast ribosomal protein L18 [*Volvox carteri* f. *nagariensis*]	0.79	0.004	7.01E-06
1103	gi|158269914|gb|EDO96007	histone H4, partial [*Chlamydomonas reinhardtii*]	0.75	0.017	0.0004277
1114	Unigene17947_All	256 735 minus strand DUF924-domain-containing protein [*Coccomyxa subellipsoidea* C-169]	0.75	0.006	1.46E-05
1116	gi|158278933|gb|EDP04695	voltage-gated Ca2+ channel, alpha subunit [*Chlamydomonas reinhardtii*]	0.83	0.038	0.02924
1117	CL6074_Contig1_All	Os11g0181700 [*Oryza sativa* Japonica Group]	0.81	0.007	0.000168
1144	Unigene18241_All	Os12g0485800 [*Oryza sativa* Japonica Group]	0.66	0.004	1.93E-07
1152	CL2211_Contig4_All	1 310 LEN=310; minus strand; translated	0.67	0.013	2.33E-05
1188	Unigene12041_All	262 654 LSU ribosomal protein L11P [*Coccomyxa subellipsoidea* C-169]	0.8	0.004	1.12E-05
1196	Unigene18937_All	1 300 LEN=300; translated	0.72	0.074	0.01432
1223	CL3098_Contig1_All	255 1142 minus strand expressed unknown protein [*Ectocarpus siliculosus*]	0.75	0.01	5.73E-05
1233	Unigene20346_All	188 529 minus strand unknown [*Picea sitchensis*]	0.77	0.017	0.0008069
1252	Unigene7614_All	predicted protein [*Chlamydomonas reinhardtii*]	0.66	0.001	3.48E-10
1260	CL6009_Contig3_All	1 238 LEN=238; translated	0.83	0.005	0.0001036
1268	CL5071_Contig1_All	1 942 LEN=1325; translated	0.51	0.011	6.86E-07
1295	CL4649_Contig1_All	1301 1999 ferritin [*Ulva fasciata*]	0.72	0.017	0.0002189

1308	Unigene18531_All	393 1376 cytochrome P450 [*Coccomyxa subellipsoidea* C-169]	0.77	0.012	0.0002477
1314	CL5995_Contig1_All	FLU chloroplast precursor, alternative spliced version s-FLP [*Chlamydomonas reinhardtii*]	0.65	0.011	9.44E-06
1321	Unigene13607_All	thioredoxin peroxidase (ISS) [*Ostreococcus tauri*]	0.78	0.014	0.0004694
1372	CL1282_Contig1_All	223 1527 vacuolar ATP synthase subunit H [*Coccomyxa subellipsoidea* C-169]	0.79	0.014	0.000711
1405	CL7251_Contig1_All	predicted protein [*Chlamydomonas reinhardtii*]	0.8	0.009	0.0002211
1410	gi|170293977|gb|ACB13082	ATP synthase beta subunit, partial (plastid) [*Ulva lactuca*]	0.76	0.023	0.00137
1426	Unigene12106_All	2 487 glutathione S-transferase [*Coccomyxa subellipsoidea* C-169]	0.73	0.007	7.65E-06
1431	Unigene44820_All	ras-related gtp-binding protein [*Micromonas* sp. RCC299]	0.79	0.017	0.001208
1432	Unigene12095_All	thioredoxin m [*Chlamydomonas reinhardtii*]	0.78	0.006	2.56E-05
1439	gi|158277384|gb|EDP03152	predicted protein, partial [*Chlamydomonas reinhardtii*]	0.81	0.021	0.004385
1440	CL2133_Contig2_All	iron-sulfur subunit of mitochondrial succinate dehydrogenase [*Chlamydomonas reinhardtii*]	0.73	0.006	5.96E-06
1452	CL1910_Contig1_All	38 1480 predicted protein [*Hordeum vulgare* subsp_ vulgare]	0.83	0.038	0.02763
1458	CL6866_Contig1_All	245 841 flagellar associated protein [*Coccomyxa subellipsoidea* C-169]	0.67	0.014	3.32E-05
1474	CL2283_Contig4_All	40S ribosomal protein S3-1 [*Arabidopsis thaliana*]	0.83	0.009	0.0007366
1477	CL1997_Contig1_All	predicted protein [*Micromonas* sp. RCC299]	0.82	0.002	4.05E-06
1482	CL1639_Contig3_All	5 1300 minus strand predicted protein [*Hordeum vulgare* subsp_ vulgare]	0.7	0.078	0.01139
1483	CL2198_Contig2_All	312 503 glutathione S-transferase [*Coccomyxa subellipsoidea* C-169]	0.69	0.005	1.33E-06
1487	gi|145568027|gb|ABP82370	ribulose-1,5-bisphosphate carboxylase/oxygenase large subunit, partial (chloroplast) [*Ulva compressa*]	0.67	0.097	0.01299
1504	Unigene5869_All	359 739 Rubredoxin-like protein [*Coccomyxa subellipsoidea* C-169]	0.76	0.011	0.0001505
1535	CL4098_Contig4_All	17 373 minus strand GRIM-19 [*Coccomyxa subellipsoidea* C-169]	0.83	0.025	0.01109
1537	CL7117_Contig1_All	189 1457 minus strand si-synthase [*Coccomyxa subellipsoidea* C-169]	0.68	0.007	3.22E-06
1538	CL5102_Contig1_All	predicted protein [*Chlamydomonas reinhardtii*]	0.52	0.007	1.06E-07
1550	CL5153_Contig2_All	69 779 chloroplast light harvesting complex I protein [*Chlamydomonas incerta*]	0.66	0.025	0.0001874
1551	CL1735_Contig2_All	predicted protein [*Chlamydomonas reinhardtii*]	0.75	0.016	0.0003214
1565	Unigene1628_All	359 562 small nuclear ribonucleo protein polypeptide G [*Coccomyxa subellipsoidea* C-169]	0.83	0.011	0.001129
1569	CL1403_Contig1_All	307 1131 minus strand NAD(P)-binding protein [*Coccomyxa subellipsoidea* C-169]	0.73	0.007	8.77E-06
1571	Unigene26288_All	3 167 Sm-like ribonucleo protein [*Coccomyxa subellipsoidea* C-169]	0.46	0.013	5.58E-07
1591	CL3743_Contig4_All	predicted protein [*Chlamydomonas reinhardtii*]	0.82	0.022	0.007299
1597	CL5085_Contig1_All	light-harvesting protein of photosystem I [*Chlamydomonas reinhardtii*]	0.77	0.055	0.0187
1601	gi|158280270|gb|EDP06028	centriole proteome protein, partial [*Chlamydomonas reinhardtii*]	0.8	0.004	8.47E-06
1605	CL2725_Contig1_All	plastid acyl-ACP desaturase [*Volvox carteri* f. *nagariensis*]	0.7	0.002	4.84E-08
1640	CL1959_Contig1_All	fructose-1,6-bisphosphate aldolase [*Chlamydomonas reinhardtii*]	0.76	0.002	4.93E-07
1657	CL4555_Contig2_All	1 578 LEN=578; minus strand; translated	0.75	0.015	0.0002676

1701	gi|158283897|gb|EDP09647	DNA polymerase delta subunit one [*Chlamydomonas reinhardtii*]	0.72	0.115	0.03663
1705	CL52_Contig1_All	glutathione S-transferase [*Chlamydomonas reinhardtii*]	0.79	0.024	0.003677
1710	Unigene24798_All	predicted protein [*Chlamydomonas reinhardtii*]	0.69	0.046	0.002339
1746	CL3520_Contig2_All	cytochrome b6-f complex subunit V [*Volvox carteri* f.*nagariensis*]	0.7	0.042	0.002479
1761	Unigene34600_All	1 207 LEN=207; translated	0.72	0.038	0.002339
1766	CL5524_Contig1_All	134 1195 NAD-dependent epimerase/dehydratase [*Coccomyxa subellipsoidea* C-169]	0.83	0.007	0.0002724
1779	CL5569_Contig1_All	49 522 minus strand nucleoside diphosphate kinase [*Hyacinthus orientalis*]	0.78	0.009	0.0001061
1804	Unigene21385_All	predicted protein [*Thalassiosira pseudonana* CCMP1335]	0.83	0.015	0.002861
1820	Unigene18279_All	501 890 minus strand nitric oxide dioxygenase [*Galdieria sulphuraria*]	0.73	0.056	0.009448
1869	gi|158277761|gb|EDP03528	predicted protein [*Chlamydomonas reinhardtii*]	0.69	0.029	0.0006453
1876	CL2806_Contig2_All	5 442 minus strand acetyl-CoA biotin carboxyl carrier [*Coccomyxa subellipsoidea* C-169]	0.68	0.014	3.58E-05
1905	CL3198_Contig1_All	1 987 ATP/ADP translocator [*Chlorella variabilis*]	0.83	0.047	0.04409
1911	Unigene33065_All	1 203 LEN=203; translated	0.67	0.013	2.59E-05
1917	CL2794_Contig3_All	250 1272 DUF1350-domain-containing protein [*Coccomyxa subellipsoidea* C-169]	0.76	0.089	0.0391
1919	CL4568_Contig1_All	glutathione S-transferase [*Chlamydomonas reinhardtii*]	0.75	0.038	0.005224
1940	Unigene1416_All	114 1103 minus strand ornithine carbamoyltransferase [*Coccomyxa subellipsoidea* C-169]	0.73	0.001	5.21E-09
1946	CL4430_Contig6_All	plastid ribosomal protein S6 [*Chlamydomonas reinhardtii*]	0.69	0.015	6.84E-05
1962	CL3843_Contig1_All	89 934 chloroplast CGE2 [*Physcomitrella patens*]	0.54	0.014	2.77E-06
1993	CL4259_Contig1_All	122 550 minus strand TPA_inf: chloroplast light-harvesting complex I protein precursor Lhca9 [*Acetabularia acetabulu*	0.72	0.01	3.15E-05
2003	CL6601_Contig2_All	1 298 LEN=298; minus strand; translated	0.8	0.021	0.003089
2004	Unigene3217_All	predicted protein [*Hordeum vulgare* subsp_ vulgare]	0.55	0.068	0.000854
2007	CL1902_Contig1_All	Os08g0282400 [*Oryza sativa* Japonica Group]	0.75	0.015	0.0002665
2018	gi|158276881|gb|EDP02651	predicted protein [*Chlamydomonas reinhardtii*]	0.77	0.008	5.03E-05
2027	CL6386_Contig2_All	223 798 uncharacterized protein LOC100794560 [*Glycine max*]	0.58	0.002	2.83E-09
2029	Unigene23953_All	178 459 mitochondrial import inner membrane translocase subunit Tim9 [*Coccomyxa subellipsoidea* C-169]	0.55	0.013	2.23E-06
2059	Unigene20237_All	flagellar associated protein [*Chlamydomonas reinhardtii*]	0.83	0.008	0.0003411
2068	gi|807046157|gb|AKC3521	magnesium-chelatase subunit ChlI (chloroplast) [*Ulva* sp.]	0.75	0.002	5.47E-08
2076	CL1608_Contig1_All	26S proteasome regulatory subunit [*Chlamydomonas reinhardtii*]	0.81	0.003	2.63E-06
2089	Unigene4025_All	202 837 minus strand ras-related protein Rab2BV [*Coccomyxa subellipsoidea* C-169]	0.82	0.008	0.0003048
2098	Unigene41410_All	tubulin beta-7 chain isoform 2 [*Zea mays*]	0.33	0.023	1.10E-06
2100	CL7825_Contig6_All	3 308 beta-tubulin [*Amphidinium carterae*]	0.74	0.044	0.005245
2124	CL1257_Contig3_All	56 547 minus strand n/a [*Ectocarpus siliculosus*]	0.46	0.094	0.0007071
2131	CL6232_Contig6_All	1 364 LEN=364; minus strand; translated	0.79	0.018	0.001325

2148	Unigene18265_All	NADH:ubiquinone oxidoreductase 22 kDa subunit [*Volvox carteri* f. *nagariensis*]	0.78	0.011	0.0002243
2149	Unigene20071_All	139 426 mitochondrial chaperonin hsp10, precursor [*Cyanidioschyzon merolae* strain 10D]	0.65	0.027	0.0001946
2190	gi|807046133|gb|AKC35191	photosystem I assembly protein (chloroplast) [*Ulva fasciata*]	0.73	0.004	1.43E-06
2199	Unigene45256_All	1 412 LEN=412; minus strand; translated	0.62	0.094	0.005802
2213	Unigene37689_All	90 521 small subunit ribosomal protein S14e, cytoplasmic [*Guillardia theta* CCMP2712]	0.74	0.019	0.0004609
2237	Unigene42486_All	cystathionine beta-synthase [*Phytophthora infestans* T30-4]	0.73	0.034	0.002144
2250	Unigene10040_All	predicted protein [*Chlamydomonas reinhardtii*]	0.6	0.011	3.89E-06
2251	gi|145567604|gb|ABP82159	ribulose-1,5-bisphosphate carboxylase/oxygenase large subunit, partial (chloroplast) [*Ulva* sp.]	0.81	0.04	0.02164
2256	CL1858_Contig1_All	rieske iron-sulfur subunit of the cytochrome b6f complex, chloroplast precursor [*Chlamydomonas reinhardtii*]	0.67	0.003	8.39E-08
2272	CL5014_Contig1_All	170 580 CR084 protein [*Chlamydomonas reinhardtii*]	0.66	0.064	0.003717
2287	Unigene18235_All	predicted protein [*Thalassiosira pseudonana* CCMP1335]	0.78	0.032	0.006232
2292	Unigene20667_All	Qc-SNARE protein, Tlg1/Syntaxin 6-family [*Chlamydomonas reinhardtii*]	0.7	0.02	0.0001864
2297	Unigene27061_All	5 505 minus strand aldolase [*Coccomyxa subellipsoidea* C-169]	0.81	0.016	0.00242
2299	gi|135404|sp|P11481_1|TBA	alpha-2 tubulin [*Volvox carteri*]	0.66	0.095	0.00993
2303	Unigene19599_All	predicted protein [*Physcomitrella patens* subsp_ patens]	0.7	0.01	2.20E-05
2310	Unigene2391_All	320 1267 periplasmic binding protein-like II, partial [*Coccomyxa subellipsoidea* C-169]	0.75	0.019	0.0006526
2327	CL3468_Contig2_All	1 295 LEN=295; minus strand; translated	0.81	0.012	0.0007291
2341	CL801_Contig2_All	6 275 PREDICTED: uncharacterized protein LOC100794023 [*Glycine max*]	0.83	0.005	0.0001032
2350	CL110_Contig1_All	predicted protein [*Chlamydomonas reinhardtii*]	0.81	0.018	0.002936
2358	CL4544_Contig1_All	1 207 LEN=207; minus strand; translated	0.78	0.065	0.0321
2379	CL4998_Contig1_All	aspartate--ammonia ligase [*Phaeodactylum tricornutum* CCAP 1055/1]	0.81	0.013	0.001119
2393	CL6567_Contig1_All	173 1033 chloroplast ascorbate peroxidase [*Chlamydomonas* sp.]	0.71	0.004	1.10E-06
2429	Unigene16018_All	NADH:ubiquinone oxidoreductase 11 kDa subunit [*Chlamydomonas reinhardtii*]	0.61	0.037	0.0003178
2481	Unigene18544_All	141 1841 mitochondriatargeted chaperonin putative [*Albugo laibachii* Nc14]	0.78	0.018	0.001246
2494	CL3922_Contig1_All	prenylated rab acceptor family protein [*Chlamydomonas reinhardtii*]	0.77	0.025	0.002142
2497	CL4595_Contig1_All	predicted protein [*Micromonas pusilla* CCMP1545]	0.74	0.009	4.09E-05
2531	Unigene43997_All	1 292 LEN=292; translated	0.76	0.043	0.007978
2544	Unigene1391_All	487 867 minus strand PREDICTED: thioredoxin-like 4, chloroplastic-like [*Glycine max*]	0.76	0.006	1.35E-05
2579	CL7451_Contig1_All	histone H2A variant 1 [*Phytophthora infestans* T30-4]	0.67	0.077	0.006858
2580	CL2770_Contig2_All	39 329 calvin cycle protein CP12 [*Volvox carteri* f. *nagariensis*]	0.78	0.012	0.0003749
2657	Unigene21104_All	1 411 LEN=411; minus strand; translated	0.81	0.021	0.00421
2671	CL433_Contig2_All	143 541 minus strand expressed unknown protein [*Ectocarpus siliculosus*]	0.76	0.009	5.19E-05
2681	CL6691_Contig2_All	MnSOD [*Ulva prolifera*]	0.62	0.001	1.70E-09

2685	gi|807046132|gb|AKC3519(	30S ribosomal protein S7 (chloroplast) [*Ulva* sp.]	0.83	0.005	0.0001217
2694	gi|158279133|gb|EDP04895	phosphate acetyltransferase [*Chlamydomonas reinhardtii*]	0.6	0.204	0.02899
2695	CL264_Contig2_All	20S proteasome alpha subunit C [*Chlamydomonas reinhardtii*]	0.72	0.017	0.0002169
2696	CL2934_Contig2_All	532 1638 cytochrome P450 [*Coccomyxa subellipsoidea* C-169]	0.83	0.005	8.32E-05
2697	gi|300256840|gb|EFJ41098_	L-ascorbate peroxidase [*Volvox carteri* f.*nagariensis*]	0.83	0.005	9.63E-05
2728	Unigene24964_All	1 365 LEN=365; translated	0.46	0.025	7.32E-06
2735	Unigene38471_All	1 791 LEN=791; minus strand; translated	0.8	0.017	0.001565
2743	Unigene3678_All	predicted protein [*Chlamydomonas reinhardtii*]	0.77	0.008	5.75E-05
2744	Unigene5984_All	thioredoxin x (ISS) [*Ostreococcus tauri*]	0.72	0.04	0.003303
2746	Unigene39581_All	predicted protein [*Phaeodactylum tricornutum* CCAP 1055/1]	0.67	0.003	1.14E-07
2775	Unigene7495_All	structural molecule, putative [*Ricinus communis*]	0.83	0.012	0.001454
2782	gi|158270666|gb|EDO96504	predicted protein [*Chlamydomonas reinhardtii*]	0.7	0.018	0.0001515
2799	gi|158282427|gb|EDP08179	light-harvesting chlorophyll-a/b protein of photosystem I [*Chlamydomonas reinhardtii*]	0.67	0.02	0.0001118
2823	Unigene12037_All	234 620 SMAD/FHA domain-containing protein [*Coccomyxa subellipsoidea* C-169]	0.67	0.011	1.24E-05
2840	CL778_Contig2_All	predicted protein [*Micromonas* sp.]	0.73	0.01	4.15E-05
2845	Unigene15945_All	2 310 unnamed protein product [*Albugo laibachii* Nc14]	0.63	0.016	2.60E-05
2851	CL3552_Contig1_All	142 750 minus strand isopropylmalate dehydratase [*Gonium pectorale*]	0.83	0.003	7.49E-06
2867	Unigene15903_All	predicted protein [*Chlamydomonas reinhardtii*]	0.81	0.004	1.27E-05
2881	CL25_Contig3_All	162 605 EF-hand [*Coccomyxa subellipsoidea* C-169]	0.51	0.081	0.0008694
2896	Unigene6735_All	1 348 LEN=348; translated	0.74	0.015	0.0002572
2898	CL4015_Contig1_All	component of oligomeric golgi complex 2 [*Volvox carteri* f. *nagariensis*]	0.81	0.015	0.001435
2899	Unigene43215_All	1 67 LEN=242; translated	0.5	0.036	5.07E-05
2902	CL2323_Contig1_All	178 1284 putative 12-oxophytodienoic acid reductase [*Coccomyxa subellipsoidea* C-169]	0.74	0.005	4.62E-06
2918	CL7338_Contig1_All	292 975 minus strand putative esterase [*Coccomyxa subellipsoidea* C-169]	0.8	0.005	2.53E-05
2927	CL4701_Contig1_All	147 1652 trigger factor [*Coccomyxa subellipsoidea* C-169]	0.81	0.004	2.64E-05
2930	gi|158271444|gb|EDO97263	predicted protein [*Chlamydomonas reinhardtii*]	0.62	0.043	0.0005609
2954	CL6020_Contig1_All	plastid-specific ribosomal protein 3 [*Chlamydomonas reinhardtii*]	0.82	0.014	0.001698
2965	CL5639_Contig2_All	81 752 minus strand chloroplast ATP synthase subunit delta precursor [*Coccomyxa subellipsoidea* C-169]	0.64	0.015	1.97E-05
2992	CL419_Contig1_All	unknown [*Picea sitchensis*]	0.61	0.004	6.46E-08
3016	Unigene46135_All	1 256 LEN=256; minus strand; translated	0.42	0.008	3.99E-08
3021	gi|158282407|gb|EDP08159	triacylglycerol lipase-like protein [*Chlamydomonas reinhardtii*]	0.66	0.021	0.0001271
3025	CL5370_Contig1_All	424 1125 adenylate kinase [*Coccomyxa subellipsoidea* C-169]	0.75	0.008	2.98E-05
3028	gi|300259803|gb|EFJ44027_	malate dehydrogenase [*Volvox carteri* f. *nagariensis*]	0.62	0.043	0.0005646

3040	Unigene24589_All	1 660 LEN=1170; minus strand; translated	0.6	0.008	8.62E-07
3067	CL738_Contig1_All	202 1266 3-isopropylmalate dehydrogenase [*Coccomyxa subellipsoidea* C-169]	0.78	0.005	1.31E-05
3069	CL1723_Contig1_All	S-like RNase [*Volvox carteri* f. *nagariensis*]	0.59	0.012	3.60E-06
3086	Unigene9808_All	13 249 TPA: 40S ribosomal protein S28, partial [*Zea mays*]	0.68	0.011	1.76E-05
3108	gi|158269490|gb|EDO95888	histone H2B variant, partial [*Chlamydomonas reinhardtii*]	0.65	0.013	1.66E-05
3116	Unigene16154_All	184 1173 aldo/keto reductase [*Coccomyxa subellipsoidea* C-169]	0.82	0.012	0.0009533
3118	Unigene14048_All	197 1099 Mov34-domain-containing protein [*Coccomyxa subellipsoidea* C-169]	0.79	0.001	4.30E-09
3150	Unigene7958_All	1 567 LEN=656; translated	0.67	0.023	0.0001997
3153	Unigene10622_All	1 1041 PREDICTED: DEAD-box ATP-dependent RNA helicase 56-like [*Fragaria vesca* subsp_ vesca]	0.7	0.012	3.33E-05
3161	CL6873_Contig1_All	229 579 minus strand unknown [*Lotus japonicus*]	0.71	0.012	5.01E-05
3168	CL4502_Contig1_All	136 798 glutathione S-transferase [*Coccomyxa* sp.]	0.39	0.026	3.56E-06
3174	Unigene38926_All	1 250 LEN=250; translated	0.74	0.007	1.58E-05
3187	CL3345_Contig1_All	20S proteasome alpha subunit D [*Chlamydomonas reinhardtii*]	0.74	0.005	4.42E-06
3207	CL666_Contig8_All	R-SNARE, Sec22-family [*Volvox carteri* f. *nagariensis*]	0.73	0.006	8.97E-06
3218	Unigene42563_All	1 871 LEN=871; minus strand; translated	0.71	0.053	0.005377
3282	CL2017_Contig1_All	222 668 minus strand GPX1b [*Chlorella* sp.]	0.75	0.008	3.70E-05
3312	CL7671_Contig2_All	gamma-glutamyl hydrolase [*Chlamydomonas reinhardtii*]	0.78	0.009	0.0001051
3323	Unigene45040_All	1 174 similar to cytochrome c [*Cyanidioschyzon merolae* strain 10D]	0.61	0.001	1.47E-10
3331	CL6946_Contig1_All	predicted protein [*Micromonas pusilla* CCMP1545]	0.54	0.005	6.22E-08
3346	Unigene15016_All	2 511 predicted protein [*Populus trichocarpa*]	0.77	0.004	3.35E-06
3356	Unigene3887_All	flagellar associated protein [*Chlamydomonas reinhardtii*]	0.67	0.006	1.29E-06
3370	Unigene27742_All	91 525 thioredoxin [*Ulva fasciata*]	0.66	0.007	1.72E-06
3371	CL1185_Contig1_All	vacuolar ATP synthase subunit E [*Volvox carteri* f. *nagariensis*]	0.72	0.001	1.69E-08
3399	gi|158277042|gb|EDP02812	predicted protein [*Chlamydomonas reinhardtii*]	0.72	0.049	0.005576
3404	CL1618_Contig1_All	predicted protein [*Populus trichocarpa*]	0.75	0.026	0.001773
3416	Unigene20191_All	307 755 LEN=755; translated	0.71	0.036	0.00165
3420	CL1432_Contig3_All	7 360 minus strand aspartate aminotransferase [*Coccomyxa subellipsoidea* C-169]	0.78	0.018	0.0009495
3425	Unigene12312_All	isomerase [*Chlamydomonas reinhardtii*]	0.77	0.002	1.72E-07
3442	CL1117_Contig1_All	predicted protein [*Chlamydomonas reinhardtii*]	0.8	0.003	4.12E-06
3465	Unigene26415_All	1 334 LEN=1036; translated	0.59	0.048	0.000499
3470	gi|158279131|gb|EDP04893	predicted protein, partial [*Chlamydomonas reinhardtii*]	0.73	0.036	0.002924
3479	Unigene28337_All	1 733 LEN=1253; minus strand; translated	0.82	0.012	0.001006
3510	CL2124_Contig2_All	Casein kinase II subunit beta-4 [Medicago truncatula]	0.59	0.013	4.77E-06

3525	gi|158277974|gb|EDP03740	predicted protein [*Chlamydomonas reinhardtii*]	0.78	0.031	0.006003
3570	CL4799_Contig1_All	63 980 predicted protein [*Hordeum vulgare* subsp_ vulgare]	0.82	0.043	0.02998
3595	Unigene5695_All	predicted protein [*Micromonas* sp.]	0.8	0.026	0.005464
3602	CL5691_Contig1_All	359 1093 isopentenyl pyrophosphate:dimethylallyl pyrophosphate isomerase [*Haematococcus pluvialis*]	0.71	0.002	4.50E-08
3609	Unigene6357_All	78 848 minus strand TPA_inf: chloroplast light-harvesting complex II protein precursor Lhcbm13 [*Acetabularia acetab*	0.7	0.037	0.001754
3636	CL1802_Contig1_All	1 409 LEN=734; translated	0.74	0.012	9.38E-05
3637	Unigene12101_All	198 632 minus strand protein mago nashi 2 [*Coccomyxa subellipsoidea* C-169]	0.82	0.002	9.92E-07
3638	CL697_Contig3_All	1994 2353 LEN=2522; minus strand; translated	0.67	0.011	1.16E-05
3642	CL1626_Contig2_All	4 501 minus strand Alpha-glucan water dikinase 1, chloroplastic OS=Arabidopsis thaliana GN=GWD1 PE=1 SV=2	0.58	0.016	8.51E-06
3659	CL3965_Contig1_All	2 253 minus strand histone H4 [*Zea mays*]	0.76	0.014	0.0003115
3660	CL649_Contig6_All	2 205 Rubisco activase [*Coccomyxa subellipsoidea* C-169]	0.76	0.025	0.001959
3668	gi|807046101|gb|AKC3515	ribosomal protein L19 (chloroplast) [*Ulva* sp.]	0.74	0.007	1.20E-05
3693	gi|848130047|pdb|2N0S|A	Chain A, Haddock Model Of Ferredoxin And [fefe] Hydrogenase Complex	0.6	0.069	0.001741
3694	Unigene15411_All	6 284 minus strand predicted protein [*Hordeum vulgare* subsp_ vulgare]	0.7	0.11	0.02664
3704	CL3970_Contig1_All	aci-reductone dioxygenase [*Volvox carteri* f. *nagariensis*]	0.62	0.015	1.44E-05
3710	CL5721_Contig1_All	predicted protein [*Micromonas* sp. RCC299]	0.83	0.019	0.00525
3734	CL1800_Contig3_All	MAP kinase phosphatase 6 [*Chlamydomonas reinhardtii*]	0.72	0.011	3.85E-05
3738	gi|158276759|gb|EDP02530	predicted protein [*Chlamydomonas reinhardtii*]	0.1	0	1.99E-05
3758	CL4357_Contig2_All	159 590 Prefoldin-domain-containing protein [*Coccomyxa subellipsoidea* C-169]	0.77	0.015	0.0004801
3781	CL3016_Contig1_All	83 844 minus strand 14-3-3-like protein-related protein [*Chlamydomonas incerta*]	0.77	0.017	0.000749
3829	Unigene3896_All	peptidyl-prolyl cis-trans isomerase, cyclophilin-type [*Chlamydomonas reinhardtii*]	0.7	0.003	1.38E-07
3871	Unigene16172_All	pyridine nucleotide binding protein [*Chlamydomonas reinhardtii*]	0.77	0.003	3.00E-06
3972	CL4365_Contig2_All	predicted protein [*Chlamydomonas reinhardtii*]	0.65	0.076	0.005442
4015	CL2952_Contig1_All	94 1143 ATP synthase gamma-subunit [*Coccomyxa subellipsoidea* C-169]	0.83	0.002	2.89E-06
4016	Unigene26995_All	predicted protein [*Phaeodactylum tricornutum* CCAP 1055/1]	0.52	0.055	0.0002895
4018	CL2067_Contig2_All	translin-like protein [*Chlamydomonas reinhardtii*]	0.73	0.006	6.31E-06
4066	Unigene11569_All	Nudix hydrolase [*Medicago truncatula*]	0.76	0.012	0.0001938
4074	Unigene22718_All	1 385 LEN=385; translated	0.67	0.101	0.01338
4075	Unigene40886_All	1 207 LEN=207; minus strand; translated	0.55	0.033	7.77E-05
4087	CL4448_Contig1_All	259 1458 minus strand ornithine transaminase [*Coccomyxa subellipsoidea* C-169]	0.83	0.004	3.84E-05
4089	CL865_Contig2_All	298 771 expressed protein [*Chlorella variabilis*]	0.7	0.005	9.73E-07
4102	CL6733_Contig1_All	90 299 minus strand n/a [*Ectocarpus siliculosus*]	0.71	0.052	0.004934
4134	CL2243_Contig1_All	119 841 minus strand Glutathione S-transferase [*Ectocarpus siliculosus*]	0.69	0.013	3.60E-05

4135	CL4653_Contig2_All	233 919 peptidase C12, ubiquitin carboxyl-terminal hydrolase 1 [*Coccomyxa subellipsoidea* C-169]	0.78	0.015	0.0006803
4147	CL756_Contig2_All	predicted protein [*Chlamydomonas reinhardtii*]	0.83	0.007	0.0002322
4151	Unigene5462_All	214 882 translation initiation factor eIF3 subunit [*Coccomyxa subellipsoidea* C-169]	0.82	0.002	9.17E-07
4158	Unigene46679_All	1 274 LEN=274; translated	0.54	0.008	3.61E-07
4208	Unigene11816_All	mitochondrial ribosomal protein L23 [*Chlamydomonas reinhardtii*]	0.73	0.014	0.000141
4225	gi|158284273|gb|EDP10023	predicted protein [*Chlamydomonas reinhardtii*]	0.36	0.02	0.01586
4231	CL925_Contig1_All	371 1426 P-loop containing nucleoside triphosphate hydrolase protein [*Coccomyxa subellipsoidea* C-169]	0.73	0.006	4.86E-06
4242	CL527_Contig8_All	48 971 minus strand tubulin alpha chain [*Coccomyxa subellipsoidea* C-169]	0.74	0.031	0.002124
4246	CL6080_Contig1_All	200 694 minus strand EF-hand [*Coccomyxa subellipsoidea* C-169]	0.8	0.003	6.24E-06
4250	CL22_Contig1_All	859 1218 S-adenosyl-L-methionine-dependent methyltransferase [*Coccomyxa subellipsoidea* C-169]	0.81	0.007	0.0001313
4255	Unigene42635_All	23 670 minus strand unnamed protein product [*Blastocystis hominis*]	0.81	0.006	6.97E-05
4259	CL6485_Contig1_All	metacaspase type II [*Chlamydomonas reinhardtii*]	0.67	0.009	7.66E-06
4276	CL4913_Contig1_All	121 720 eukaryotic translation initiation factor 4E [*Coccomyxa subellipsoidea* C-169]	0.77	0.008	4.55E-05
4283	Unigene3579_All	313 2025 RNI-like protein [*Coccomyxa subellipsoidea* C-169]	0.81	0.005	3.12E-05
4291	Unigene5701_All	predicted protein [*Micromonas* sp. RCC299]	0.7	0.015	6.41E-05
4344	Unigene11809_All	predicted protein [*Populus trichocarpa*]	0.71	0.008	9.50E-06
4363	Unigene31256_All	predicted protein [*Phaeodactylum tricornutum* CCAP 1055/1]	0.74	0.005	3.50E-06
4383	gi|807046151|gb|AKC3520	50S ribosomal protein L12 (chloroplast) [*Ulva fasciata*]	0.57	0.047	0.0003503
4385	Unigene7520_All	predicted protein [*Populus trichocarpa*]	0.73	0.021	0.0005173
4395	CL348_Contig1_All	predicted protein [*Micromonas* sp. RCC299]	0.57	0.006	1.83E-07
4408	Unigene3531_All	predicted protein [*Ostreococcus lucimarinus* CCE9901]	0.69	0.005	7.42E-07
4415	CL3087_Contig4_All	molecular chaperone [*Volvox carteri* f. *nagariensis*]	0.8	0.048	0.02535
