# Supplementary material for: Integration of deep transcriptome and proteome analyses of salicylic acid regulation high temperature stress in Ulva prolifera
Source: Sci Rep. 2017 Sep 8;7:11052. doi: 10.1038/s41598-017-11449-w (PMC5591278; doi:10.1038/s41598-017-11449-w)
Supplement: Supplementary file 3 — Supplementary File 2 [file 41598_2017_11449_MOESM3_ESM.pdf]

UpSHT-VS-UpHT_Cor_DEPs

NO.	Protein ID	Protein Description	Protein Ratio(U	DEPs	Correlation gene ID	Gene Ratio(UpSH	Gene Sig	DEGs
88	Unigene43358_All	1 472 LEN=472; minus strand; translated	0.78	-	Unigene43358_All	11.59030634	9.00E-08	+
188	CL5484_Contig2_All	ribosomal protein S6 [*Chlamydomonas reinhardtii*]	1.21	+	CL5484.Contig2_All	-14.39309545	5.11E-24	-
234	Unigene20469_All	hypothetical protein VOLCADRAFT_33421 [*Volvox carteri* f. *nagariensis*]	1.22	+	Unigene20469_All	-12.09691277	5.11E-18	-
259	Unigene29891_All	243 452 predicted protein [*Bathycoccus prasinos*]	1.48	+	Unigene29891_All	-11.05819471	4.09E-05	-
376	Unigene20819_All	86 700 minus strand F1F0 ATP synthase gamma subunit [*Coccomyxa subellipsoidea* C-169]	0.78	-	Unigene20819_All	-13.13894371	1.73E-33	-
386	CL4608_Contig2_All	ribosomal protein [*Micromonas pusilla* CCMP1545]	0.83	-	CL4608.Contig2_All	-15.35761729	3.32E-48	-
388	CL4038_Contig3_All	2 259 PREDICTED: cytochrome c1-1, heme protein, mitochondrial-like [*Glycine max*]	0.75	-	CL4038.Contig3_All	-12.39526635	2.68E-07	-
397	CL206_Contig2_All	1 477 PREDICTED: elongation factor 2-like [*Solanum lycopersicum*]	1.81	+	CL206.Contig2_All	-13.6903909	1.73E-33	-
695	Unigene6725_All	53 280 minus strand polyubiquitin [*Aureococcus anophagefferens*]	0.83	-	Unigene6725_All	-1.247754376	2.69E-14	-
749	Unigene23985_All	1 153 hypothetical protein COCSUDRAFT_21435 [*Coccomyxa subellipsoidea* C-169]	0.64	-	Unigene23985_All	-12.90906802	4.63E-12	-
762	Unigene27002_All	71 409 DNA-directed RNA polymerase II subunit RPB11-like isoform 2 [*Fragaria vesca* subsp_ vesca]	0.71	-	Unigene27002_All	-12.57530288	1.78E-31	-
848	Unigene1814_All	3 152 17_8 kDa class I heat shock protein OS=*Arabidopsis thaliana*	0.73	-	Unigene1814_All	1.67169868	3.35E-33	+
853	Unigene28145_All	predicted protein [*Chlamydomonas reinhardtii*]	0.78	-	Unigene28145_All	-11.87486611	1.32E-14	-
978	Unigene163_All	438 1154 minus strand glutathione S-transferase [*Coccomyxa subellipsoidea* C-169]	0.64	-	Unigene163_All	-1.026349519	0	-
1061	CL3256_Contig5_All	component of TRAPP complex [*Chlamydomonas reinhardtii*]	0.71	-	CL3256.Contig5_All	-12.14996935	2.67E-07	-
1064	Unigene39765_All	predicted protein [*Thalassiosira pseudonana* CCMP1335]	0.67	-	Unigene39765_All	3.308920564	0.000474899	+
1084	Unigene41911_All	4 243 minus strand hypothetical protein AURANDRAFT_60228 [*Aureococcus anophagefferens*]	0.61	-	Unigene41911_All	12.2340989	2.67E-06	+
1196	Unigene18937_All	1 300 LEN=300; translated	0.72	-	Unigene18937_All	-1.445020259	1.30E-09	-
1452	CL1910_Contig1_All	38 1480 predicted protein [*Hordeum vulgare* subsp_ vulgare]	0.83	-	CL1910.Contig1_All	-1.153852371	4.82E-09	-
1482	CL1639_Contig3_All	5 1300 minus strand predicted protein [*Hordeum vulgare* subsp_ vulgare]	0.7	-	CL1639.Contig3_All	-1.120569797	5.04E-10	-
1535	CL4098_Contig4_All	17 373 minus strand GRIM-19 [*Coccomyxa subellipsoidea* C-169]	0.83	-	CL4098.Contig4_All	-11.41653366	2.21E-05	-
1595	Unigene20506_All	predicted protein [*Chlamydomonas reinhardtii*]	1.3	+	Unigene20506_All	-11.61503184	2.26E-10	-
1604	CL6191_Contig2_All	6 485 hypothetical protein CHLNCDRAFT_31039 [*Chlorella variabilis*]	1.36	+	CL6191.Contig2_All	-10.58233019	0.000471522	-
1645	Unigene42861_All	458 1384 minus strand hypothetical protein CHLNCDRAFT_54582 [Chlorella variabilis]	1.86	+	Unigene42861_All	5.361527146	5.53E-52	+
1657	CL4555_Contig2_All	1 578 LEN=578; minus strand; translated	0.75	-	CL4555.Contig2_All	-11.12166302	9.53E-07	-
1710	Unigene24798_All	predicted protein [*Chlamydomonas reinhardtii*]	0.69	-	Unigene24798_All	-11.11608399	7.63E-05	-
1746	CL3520_Contig2_All	cytochrome b6-f complex subunit V [*Volvox carteri* f. *nagariensis*]	0.7	-	CL3520.Contig2_All	-12.73963296	5.17E-22	-
1747	Unigene28169_All	126 341 minus strand hypothetical protein COCSUDRAFT_53064 [*Coccomyxa subellipsoidea* C-16	0.63	-	Unigene28169_All	-11.72715467	8.19E-10	-
1857	CL1385_Contig1_All	25 1437 minus strand translation elongation factor-like protein [*Karlodinium veneficum*]	1.31	+	CL1385.Contig1_All	4.744745412	9.42E-64	+
1876	CL2806_Contig2_All	5 442 minus strand acetyl-CoA biotin carboxyl carrier [*Coccomyxa subellipsoidea* C-169]	0.68	-	CL2806.Contig2_All	-12.5609776	7.20E-19	-
1903	Unigene41717_All	13 261 hypothetical protein GUITHDRAFT_166560 [*Guillardia theta* CCMP2712]	0.73	-	Unigene41717_All	11.99251354	1.01E-05	+

1905	CL3198_Contig1_All	1 987 ATP/ADP translocator [*Chlorella variabilis*]	0.83	-	CL3198.Contig1_All	-14.56001631	2.93E-160	-
1925	CL5846_Contig3_All	5 568 hypothetical protein COCSUDRAFT_52084 [*Coccomyxa subellipsoidea* C-169]	0.82	-	CL5846.Contig3_All	-11.43447186	4.33E-10	-
2004	Unigene3217_All	predicted protein [*Hordeum vulgare* subsp_ vulgare]	0.55	-	Unigene3217_All	-1.52404299	1.41E-05	-
2029	Unigene23953_All	178 459 mitochondrial import inner membrane translocase subunit Tim9 [*Coccomyxa subellipsoidea* (	0.55	-	Unigene23953_All	-12.2423405	6.88E-15	-
2050	Unigene24492_All	2 367 hypothetical protein CHLNCDRAFT_138900 [*Chlorella variabilis*]	0.83	-	Unigene24492_All	-11.59399795	6.20E-11	-
2098	Unigene41410_All	tubulin beta-7 chain isoform 2 [*Zea mays*]	0.33	-	Unigene41410_All	4.34632624	2.33E-09	+
2100	CL7825_Contig6_All	3 308 beta-tubulin [*Amphidinium carterae*]	0.74	-	CL7825.Contig6_All	-1.398543637	0.000119928	-
2124	CL1257_Contig3_All	56 547 minus strand n/a [*Ectocarpus siliculosus*]	0.46	-	CL1257.Contig3_All	-1.145592798	0.000115953	-
2213	Unigene37689_All	90 521 small subunit ribosomal protein S14e, cytoplasmic [*Guillardia theta* CCMP2712]	0.74	-	Unigene37689_All	6.409848674	6.28E-46	+
2237	Unigene42486_All	cystathionine beta-synthase [*Phytophthora infestans* T30-4]	0.73	-	Unigene42486_All	5.346913748	2.32E-10	+
2240	CL3741_Contig1_All	hypothetical protein VOLCADRAFT_104059 [*Volvox carteri* f. *nagariensis*]	0.37	-	CL3741.Contig1_All	-11.92280737	9.74E-16	-
2292	Unigene20667_All	Qc-SNARE protein, Tlg1/Syntaxin 6-family [*Chlamydomonas reinhardtii*]	0.7	-	Unigene20667_All	-11.33656235	9.54E-07	-
2297	Unigene27061_All	5 505 minus strand aldolase [*Coccomyxa subellipsoidea* C-169]	0.81	-	Unigene27061_All	-12.31424335	6.98E-27	-
2307	Unigene12347_All	60S ribosomal protein L3 [*Phytophthora infestans* T30-4]	0.78	-	Unigene12347_All	-2.244914624	3.01E-21	-
2336	CL15_Contig5_All	hypothetical protein VOLCADRAFT_108821 [*Volvox carteri* f. *nagariensis*]	0.83	-	CL15.Contig5_All	-11.86015583	1.80E-06	-
2439	Unigene15923_All	hypothetical protein [*Volvox carteri f. nagariensis*]	0.76	-	Unigene15923_All	1.134970297	8.31E-116	+
2481	Unigene18544_All	141 1841 mitochondriatargeted chaperonin putative [*Albugo laibachii* Nc14]	0.78	-	Unigene18544_All	-2.11409961	4.97E-47	-
2497	CL4595_Contig1_All	predicted protein [*Micromonas pusilla* CCMP1545]	0.74	-	CL4595.Contig1_All	-12.88195516	1.85E-15	-
2538	CL7534_Contig2_All	71 907 minus strand TPA_inf: chloroplast light-harvesting protein CP29 precursor Lhcb4 [*Acetabular*	2.51	+	CL7534.Contig2_All	-16.0369098	0	-
2579	CL7451_Contig1_All	histone H2A variant 1 [*Thalassiosira oceanica*]	0.67	-	CL7451.Contig1_All	6.544426265	9.23E-51	+
2583	Unigene42867_All	15 665 minus strand hypothetical protein THAOC_09311 [*Thalassiosira oceanica*]	0.75	-	Unigene42867_All	12.5245662	4.84E-24	+
2641	Unigene42677_All	aspartate aminotransferase [*Volvox carteri* f. *nagariensis*]	1.21	+	Unigene42677_All	6.318410755	6.55E-43	+
2657	Unigene21104_All	1 411 LEN=411; minus strand; translated	0.81	-	Unigene21104_All	-11.83597143	7.43E-08	-
2701	Unigene5720_All	40S ribosomal protein SA [*Phytophthora infestans* T30-4]	0.7	-	Unigene5720_All	-1.379689202	2.99E-06	-
2728	Unigene24964_All	1 365 LEN=365; translated	0.46	-	Unigene24964_All	-4.260642039	0.00011003	-
2735	Unigene38471_All	1 791 LEN=791; minus strand; translated	0.8	-	Unigene38471_All	3.47609184	1.12E-06	+
2800	Unigene25141_All	244 420 hypothetical protein COCSUDRAFT_60467 [*Coccomyxa subellipsoidea* C-169]	0.8	-	Unigene25141_All	-11.45681641	3.40E-06	-
2891	CL4698_Contig2_All	4 393 minus strand oxoglutarate:malate antiporter, partial [*Coccomyxa subellipsoidea* C-169]	1.25	+	CL4698.Contig2_All	-11.17336483	7.52E-05	-
2899	Unigene43215_All	1 67 LEN=242; translated	0.5	-	Unigene43215_All	11.43858396	0.000950994	+
2943	Unigene27603_All	152 478 minus strand hypothetical protein COCSUDRAFT_52340 [*Coccomyxa subellipsoidea* C-16	0.77	-	Unigene27603_All	-13.26971112	4.71E-32	-
3014	Unigene20747_All	hypothetical protein VOLCADRAFT_75893 [*Volvox carteri* f. *nagariensis*]	1.72	+	Unigene20747_All	-14.86731736	2.30E-125	-
3040	Unigene24589_All	1 660 LEN=1170; minus strand; translated	0.6	-	Unigene24589_All	-11.40199944	1.36E-16	-
3153	Unigene10622_All	1 1041 PREDICTED: DEAD-box ATP-dependent RNA helicase 56-like [*Fragaria vesca* subsp_ vesca	0.7	-	Unigene10622_All	-1.429435098	0.00023927	-

3207	CL666_Contig8_All	R-SNARE, Sec22-family [*Volvox carteri* f. *nagariensis*]	0.73	-	CL666.Contig8_All	-11.4425807	5.07E-07	-
3218	Unigene42563_All	1 871 LEN=871; minus strand; translated	0.71	-	Unigene42563_All	6.816007011	7.44E-31	+
3370	Unigene27742_All	91 525 thioredoxin [*Ulva fasciata*]	0.66	-	Unigene27742_All	-12.39903754	1.90E-17	-
3420	CL1432_Contig3_All	7 360 minus strand aspartate aminotransferase [*Coccomyxa subellipsoidea* C-169]	0.78	-	CL1432.Contig3_All	-12.53371907	3.23E-11	-
3436	Unigene14533_All	1 1915 LEN=1915; translated	1.23	+	Unigene14533_All	-2.354140557	3.27E-24	-
3465	Unigene26415_All	1 334 LEN=1036; translated	0.59	-	Unigene26415_All	-11.96159439	1.00E-21	-
3479	Unigene28337_All	1 733 LEN=1253; minus strand; translated	0.82	-	Unigene28337_All	-11.57018777	5.17E-20	-
3550	CL705_Contig3_All	1406 2563 minus strand Six-hairpin glycosidase [*Coccomyxa subellipsoidea* C-169]	1.29	+	CL705.Contig3_All	1.823885217	1.71E-21	+
3579	CL1055_Contig7_All	178 1131 minus strand PREDICTED: glucose-6-phosphate 1-dehydrogenase 2, chloroplastic-like isof	0.83	-	CL1055.Contig7_All	-12.33642287	6.71E-31	-
3642	CL1626_Contig2_All	4 501 minus strand Alpha-glucan water dikinase 1, chloroplastic OS=Arabidopsis thaliana GN=GWD	0.58	-	CL1626.Contig2_All	-11.52840527	2.07E-08	-
3659	CL3965_Contig1_All	2 253 minus strand histone H4 [*Zea mays*]	0.76	-	CL3965.Contig1_All	11.45059268	0.000503646	+
3660	CL649_Contig6_All	2 205 Rubisco activase [*Coccomyxa subellipsoidea* C-169]	0.76	-	CL649.Contig6_All	-11.83249448	0.000474827	-
3749	CL930_Contig2_All	glutamate-1-semialdehyde aminotransferase [*Chlamydomonas reinhardtii*]	1.65	+	CL930.Contig2_All	-13.70768267	1.31E-78	-
3808	CL1090_Contig3_All	74 325 minus strand hypothetical protein CHLNCDRAFT_36210 [*Chlorella variabilis*]	0.79	-	CL1090.Contig3_All	-11.96076219	1.38E-22	-
3882	CL7030_Contig6_All	predicted protein [*Hordeum vulgare* subsp_ vulgare]	1.34	+	CL7030.Contig6_All	6.263759622	3.07E-124	+
3894	CL5036_Contig2_All	hypothetical protein VOLCADRAFT_107252 [*Volvox carteri* f. *nagariensis*]	0.79	-	CL5036.Contig2_All	-12.22941969	3.25E-11	-
3970	Unigene27569_All	51 314 hypothetical protein CHLNCDRAFT_144518 [*Chlorella variabilis*]	0.7	-	Unigene27569_All	-14.0386104	1.07E-40	-
4016	Unigene26995_All	predicted protein [*Phaeodactylum tricornutum* CCAP 1055/1]	0.52	-	Unigene26995_All	-12.43478458	5.16E-22	-
4105	Unigene20624_All	hypothetical protein VOLCADRAFT_105550 [*Volvox carteri* f. *nagariensis*]	0.83	-	Unigene20624_All	-11.40178617	9.54E-07	-
4139	Unigene42972_All	1 734 LEN=734; translated	1.42	+	Unigene42972_All	5.646025508	8.19E-39	+
4210	CL1055_Contig8_All	2 682 minus strand hypothetical protein CHLNCDRAFT_134643 [*Chlorella variabilis*]	0.8	-	CL1055.Contig8_All	-13.00629101	1.11E-38	-
4255	Unigene42635_All	23 670 minus strand unnamed protein product [*Blastocystis hominis*]	0.81	-	Unigene42635_All	12.18753766	9.28E-17	+
4301	Unigene2464_All	78 1415 minus strand eukaryotic translation elongation factor 1 alpha [*Boodlea coacta*]	1.51	+	Unigene2464_All	2.255374484	2.16E-70	+
